# *Krueppel*-like factor 15 regulates Wnt/β-catenin transcription and controls cardiac progenitor cell fate in the postnatal heart

**DOI:** 10.1002/emmm.201101043

**Published:** 2012-07-05

**Authors:** Claudia Noack, Maria-Patapia Zafiriou, Hans-Joerg Schaeffer, Anke Renger, Elena Pavlova, Rainer Dietz, Wolfram H Zimmermann, Martin W Bergmann, Laura C Zelarayán

**Affiliations:** 1 Heart Research Center Göttingen (HRCG), University Medical Center Göttingen, Georg-August-University GöttingenGermany; 2 Max-Delbrück-Center for Molecular MedicineBerlin-Buch, Germany; 3 Max Planck Institute of BiochemistryMartinsried, Germany; 4 Department of Cardiology, Asklepios Klinik St. GeorgHamburg, Germany

**Keywords:** cardiac progenitor cell fate, endothelial cells, heart remodelling, KLF15, Wnt signalling

## Abstract

Wnt/β-catenin signalling controls adult heart remodelling in part via regulation of cardiac progenitor cell (CPC) differentiation. An enhanced understanding of mechanisms controlling CPC biology might facilitate the development of new therapeutic strategies in heart failure. We identified and characterized a novel cardiac interaction between *Krueppel*-like factor 15 and components of the Wnt/β-catenin pathway leading to inhibition of transcription. *In vitro* mutation, reporter assays and co-localization analyses revealed that KLF15 requires both the C-terminus, necessary for nuclear localization, and a minimal N-terminal regulatory region to inhibit transcription. In line with this, functional *Klf15* knock-out mice exhibited cardiac β-catenin transcriptional activation along with functional cardiac deterioration in normal homeostasis and upon hypertrophy. We further provide *in vivo* and *in vitro* evidences for preferential endothelial lineage differentiation of CPCs upon KLF15 deletion. Via inhibition of β-catenin transcription, KLF15 controls CPC homeostasis in the adult heart similar to embryonic cardiogenesis. This knowledge may provide a tool for reactivation of this apparently dormant CPC population in the adult heart and thus be an attractive approach to enhance endogenous cardiac repair.

## INTRODUCTION

A couple of studies have shown the capacity of adult mammalian heart cells to proliferate especially after injury (Beltrami et al, 2001; Bergmann et al, 2009; Hsieh et al, 2007; Kajstura et al, 2004). Specific groups of putative cardiac progenitor cell (CPC) populations have been identified and characterized including c-kit (Beltrami et al, 2003), Islet-1 (Laugwitz et al, 2005; Moretti et al, 2006; Wang et al, 2006), Sca1 (Oh et al, 2003), side population cells (Pfister et al, 2005, 2010) and cardiac neural crest-derived cells (Tomita et al, 2005). However, some studies showed insignificant rate of cardiac proliferation (Rubart & Field, 2006). Nonetheless, the extent to which these cells contribute to tissue homeostasis and/or repair as well as the signalling pathways controlling their proliferation and differentiation remains unclear. This scenario leads to a lack of robust endogenous cardiac regeneration to replace the infarcted myocardium. Therefore, understanding the mechanisms that control progenitor cells during normal cardiac homeostasis and repair is the basis for developing new therapeutic strategies to activate these cells for effective regeneration.

The notion that genes involved in early cardiogenesis may be re-employed to protect or regenerate heart muscle has motivated efforts to understand early developmental pathways (Epstein & Parmacek, 2005; Oka et al, 2007). One important factor regulating progenitor cell biology during early cardiogenesis is the canonical Wnt/β-catenin pathway. Active Wnt/β-catenin signalling induces the stabilization of cytosolic β-catenin, which then associates with lymphoid enhancer factor and T-cell factor, such as transcription factor 7-like 2 (TCF7L2 or TCF4) to form a transcription complex that activates Wnt target genes. This cascade is subject to multiple levels of negative control where, among others, the Nemo-like kinase (NLK) play an important role by interacting with and phosphorylating TCF to repress β-catenin/TCF-mediated transcription (Ishitani et al, 2003). Wnt/β-catenin signalling controls maintenance, proliferation and differentiation of embryonic cardiovascular progenitors in a multi-phasic fashion. Initially, Wnt/β-catenin pathway has a positive effect on embryonic mesodermal cell activation and proliferation. Subsequent inactivation is required for proper differentiation into functional cardiomyocytes while default activation leads the cells to a haemangioblast lineage (Gessert & Kuhl, 2010; Naito et al, 2006; Ueno, 2007). Interestingly, gene expression profiling analysis indicated a participation of Wnt signalling also in pathological myocardial remodelling (LaFramboise et al, 2005; Toischer et al, 2010). We previously demonstrated that cardiac β-catenin downregulation is required for adaptive hypertrophy and enhances endogenous CPC cardiomyogenic differentiation after infarction (Baurand et al, 2007; Zelarayan et al, 2008). Collectively, the available data argues strongly for a pivotal role of Wnt/β-catenin signalling in embryogenic and postnatal heart.

*Krueppel*-like factors (KLF) are a large family of zinc finger-containing transcription factors involved in diverse arrays of cellular processes including regulation of cell differentiation, cardiac remodelling, haematopoiesis, angiogenesis and stem cell-fate determination by interacting with co-activators and co-repressors (Kaczynski et al, 2003). Recent studies revealed the important role of KLF15 as a central regulator of stress response and repressor of pathological cardiac hypertrophy and fibrosis by controlling factors such as GATA binding protein 4 (GATA4), myocyte enhancer factor-2 (MEF2), myocardin (Fisch et al, 2007; Leenders et al, 2010) and the transforming growth factor β (TGFβ) (Wang et al, 2008), respectively. However, the role of KLF15 in stem cell biology of the adult heart remains unexplored.

Here, we report a novel interaction between KLF15 and components of the Wnt pathway resulting in an inhibition of β-catenin/TCF-transcriptional activity in cardiac cells. The biological role of this interaction was investigated *in vitro* and *in vivo* in the postnatal hearts of mice with a global KLF15 functional deletion. These mice exhibited a cardiac β-catenin/TCF-transcriptional de-repression and cardiac dysfunction. Deletion of KLF15 results in a constitutive β-catenin transcriptional activation that directs the CPCs to an endothelial phenotype. Collectively, our data underscore the relevance of KLF15 and the Wnt/β-catenin pathway for cardiac cellular homeostasis.

## RESULTS

### KLF15 interacts with β-catenin, NLK and TCF4 in cardiac cells

**Figure 1:**
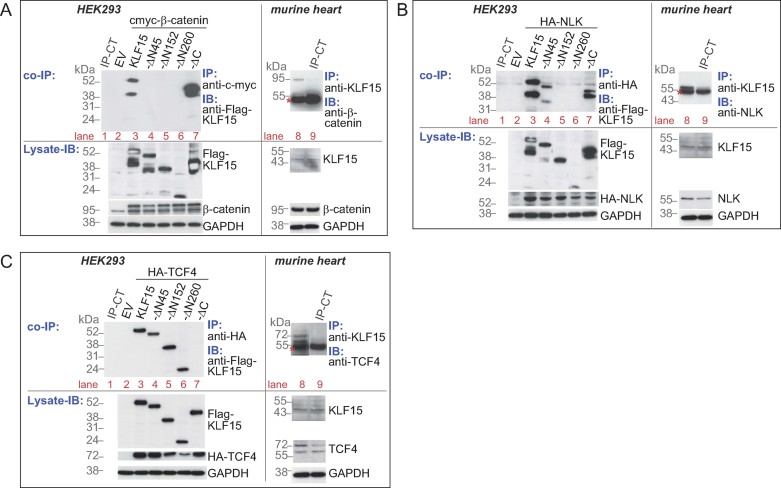
KLF15 interacts with β-catenin, NLK and TCF4 The transfected constructs in **A**, **B** and **C** were detected in the protein lysates. IgG isotype (IP-CT) (lane 1) and empty vector (EV) (lane 2) were used as controls; GAPDH served as loading control. (**A**, **B** and **C**) Endogenous validation of the identified interactions between KLF15 and β-catenin, NLK as well as TCF4 in adult cardiac tissue are depicted (lane 8). Lane 9 shows the IgG isotype control for IP. Endogenous detection of KLF15, β-catenin, NLK as well as TCF4 in the different lysates is shown. (*) IgG heavy chain (50 kDa). Co-immunoprecipitation (IP) analysis showing interaction of KLF15 and β-catenin in HEK293 cells overexpressing cmyc-β-catenin and Flag-KLF15-full-length (lane 3). Co-transfection of truncated (ΔN45, ΔN152, ΔN260)-KLF15 (lane 4–6) or a ΔC-KLF15 (lane 7) and cmyc-β-catenin plasmids in HEK293 cells showing the requirement of the first 45 amino acids of KLF15 for binding β-catenin.Interaction of NLK with KLF15-full-length, -ΔN45 (lane 3 and 4) and -ΔC (lane 7) were shown by co-IP assays in HEK293.TCF4 interacted with KLF15-full-length and all N-terminal mutants (lane 3–6), but not with the -ΔC construct (lane 7) in HEK293.

Based on previous observations concerning β-catenin and its beneficial role in cardiac remodelling, we sought to identify new β-catenin interaction partners by means of a yeast-two-hybrid screen. We identified and characterized a specific interaction between β-catenin and a member of the *Krueppel*-like transcription factor family, KLF15. To validate this interaction, we initially co-transfected the human embryonic cells HEK293 with cmyc-β-catenin and Flag-KLF15-full-length plasmids. β-catenin was immunoprecipitated with an anti-c-myc antibody and KLF15 detection was performed with an anti-Flag antibody. A clear band in the KLF15 expected size was detected, thus, confirming the interaction between KLF15 and β-catenin (Fig 1A, lane 3). Moreover, we verified the endogenous interaction of these proteins using antibodies against β-catenin and KLF15 in adult mouse cardiac tissue (Fig 1A, lane 8). To identify the binding domain, HEK293 were co-transfected with cmyc-β-catenin and N-terminal mutants lacking the first 45, 152 and 260 amino acids (ΔN45, ΔN152, ΔN260) or a C-terminal truncated KLF15 (-ΔC) plasmid. Co-immunoprecipitation revealed that deletion of the 260 N-terminal amino acids in the KLF15 sequence abrogated the β-catenin binding (Fig 1A, lane 4–6), while lack of the KLF15 C-terminus did not play a role (Fig 1A, lane 7).

Next, we investigated the potential interaction of additional factors among the Wnt/β-catenin components. By co-immunoprecipitation we found that the NLK, a known inhibitor of the β-catenin-LEF/TCF complex (Ishitani et al, 1999; Yamada et al, 2006), interacted with KLF15. HEK293 cell lysates transfected with Flag-KLF15-full-length and haemagglutinin (HA)-NLK-full-length were immunoprecipitated with an anti-HA antibody and KLF15 detection was performed with an anti-Flag antibody. The endogenous interaction was further confirmed in adult mouse cardiac cells (Fig 1B, line 3 and 8). Flag-KLF15-(ΔN45, ΔN152, ΔN260 and -ΔC) and HA-NLK-full-length were transfected in HEK293 cells to identify binding domains. Co-immunoprecipitation revealed that KLF15-ΔN45 and -ΔC but not the KLF15-ΔN152 and ΔN260 interacted with NLK (Fig 1B, line 4–7).

Since NLK interacts with and phosphorylates TCF to repress β-catenin/TCF-mediated transcription (Ishitani et al, 2003), we tested KLF15 and TCF4 interaction. HEK293 cells were co-transfected with a HA-TCF4 and Flag-KLF15-full-length. TCF4 was immunoprecipitated with an anti-HA antibody and KLF15 was detected using an anti-Flag antibody. This analysis showed interaction of KLF15 with TCF4, which was further confirmed endogenously in adult mouse cardiac cells (Fig 1C, line 3 and 8). TCF4 interaction with KLF15-ΔN45, -ΔN152, -ΔN260, -ΔC mutants was tested in HEK293 cells. Co-immunoprecipitation showed interaction of TCF4 with all the N-terminal KLF15-mutants but not with the KLF15-ΔC (Fig 1C, line 4–7). As positive controls β-catenin/TCF4 and β-catenin/NLK known interactions were confirmed in HEK293 cells (Supporting Information Fig S1A). The corresponding controls for the KLF15 antibody used for endogenous detection are provided in Supporting Information Fig S1B.

Altogether these data show the association of KLF15 to the β-catenin/TCF complex, where the first 45 amino acids of KLF15 are essential for binding β-catenin, amino acids 45–152 for binding NLK and the C-terminal region for TCF4.

### KLF15 requires the C-terminal domain for nuclear localization

**Figure 2:**
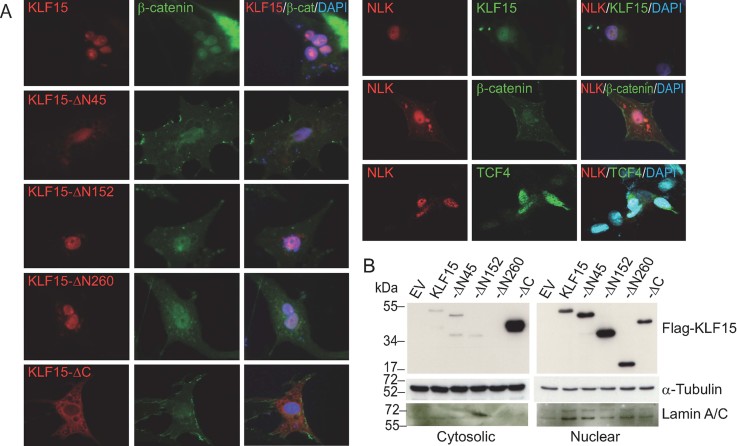
KLF15 requires the C-terminus for nuclear localization Co-localization assays in NRC show that KLF15-full-length as well as KLF15-N-terminal mutants (ΔN45, ΔN152 and ΔN260) co-localized with β-catenin in the nucleus, while KLF15-ΔC was localized in the cytosol. KLF15, β-catenin and TCF4 were detected together with NLK in the nuclear compartment.Immunoblots of enriched cell fractions confirming the subcellular localization of KLF15-ΔN forms in the nucleus and the ΔC-construct in the cytosol. Tubulin and Lamin A/C served as loading controls for cytosolic and nuclear lysates, respectively.

To localize the KLF15/β-catenin and KLF15/NLK interactions, we detected the endogenous expression of these proteins in neonatal rat cardiomyocytes (NRC) by immunofluorescence. We observed a co-localization of β-catenin and KLF15 as well as NLK and KLF15 in the nucleus (Fig 2A) suggesting the role of KLF15 as transcriptional regulator. NRC were co-transfected with both β-catenin and either Flag-KLF15-full-length or the indicated mutants. Immunofluorescence analysis showed co-localization of the KLF15-full-length as well as the N-terminal mutants of KLF15 with β-catenin in the nucleus, while the KLF15 construct lacking the C-terminal domain was exclusively found in the cytoplasm (Fig 2A). Immunoblot of enriched-cell fractions confirmed the nuclear localization of KLF15-ΔN mutants and the cytosolic localization of KLF15-ΔC (Fig 2B) suggesting that the C-terminal domain responsible for TCF binding is important for nuclear translocation.

### The N-terminal KLF15 domain mediates inhibition of β-catenin/TCF4 transcriptional activity

**Figure 3:**
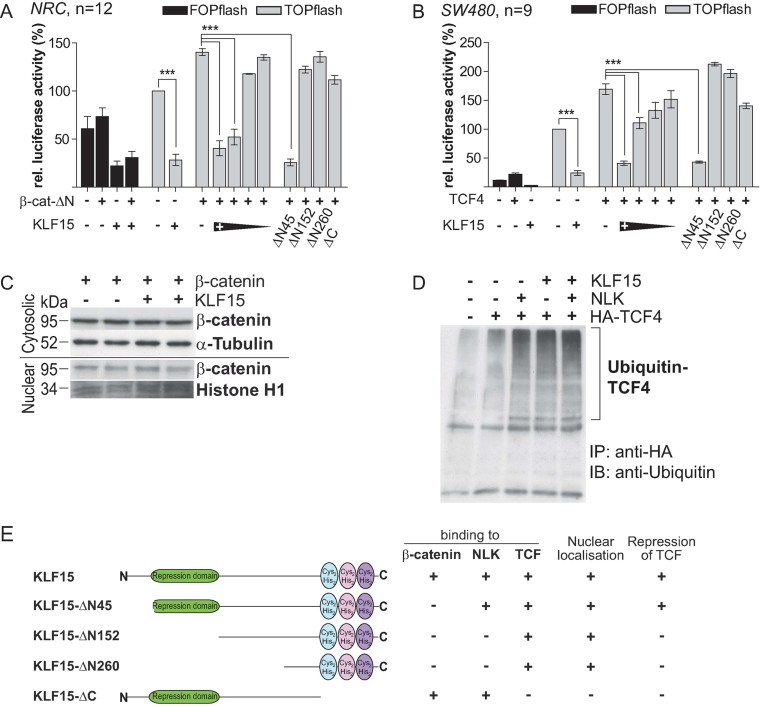
KLF15 inhibits β-catenin/TCF-transcriptional activity via its N-terminal domain and promotes degradation of TCF4 The activation of the LEF/TCF-dependent luciferase reporter (pTOPflash) upon endogenous and stabilized β-catenin (β-cat-ΔN) was significantly inhibited by KLF15 in a concentration-dependent manner (250 ng–250 pg) in NRC. Among the KLF15 N-terminal mutants (ΔN45, ΔN152 and ΔN260) only KLF15-ΔN45 suppressed the LEF/TCF reporter activation. KLF15 mutant lacking the C-terminus (-ΔC) was not able to repress LEF/TCF reporter activation.Similarly, TCF4-induced reporter activation was significantly inhibited by KLF15 full-length as well as by KLF15-ΔN45 in SW480 cells, containing constitutive active transcriptional β-catenin/TCF activity. KLF15 constructs lacking longer N-terminal regions and KLF15-ΔC did not show repression. *pFOPflash*: negative control; *Renilla* luciferase expression was used for normalization. Data are mean ± SEM; ANOVA, Bonferroni's multiple comparison test, ***p < 0.001.Immunoblot of cytosolic and nuclear fraction of β-catenin in KLF15 transfected HEK293 showing no change in β-catenin protein levels.Co-transfection of KLF15 with TCF4 resulted in enhanced TCF4 ubiquitination, which was comparable to NLK/TCF4 co-expression.Summary of the identified protein interaction, subcellular localization and transcriptional repression of the different KLF15 constructs.

We further investigated the role of these interactions in β-catenin signalling in NRC and HEK293 employing the pTOPflash reporter plasmid, which is activated when β-catenin binds to LEF/TCF elements driving firefly luciferase expression. KLF15 expression suppressed endogenous β-catenin-induced luciferase activity in NRC (*p* < 0.001, Fig 3A) and in HEK293 cells (Supporting Information Fig S1B). Furthermore, co-transfection of a stabilized form of β-catenin (β-cat-ΔN) was used to increase reporter activity. KLF15 expression was able to suppress the β-cat-ΔN-induced luciferase in NRC (*p* < 0.001, Fig 3A) and in HEK293 cells (Supporting Information Fig S1B) in a concentration-dependent manner. Next, we tested the effect of KLF15 on TCF-mediated signalling in SW480 cells, a tumour cell line that has constitutive active transcriptional β-catenin/TCF activity. Likewise, KLF15 repressed the endogenous and TCF4-induced luciferase expression in SW480 cells (*p* < 0.001, Fig 3B). Analysis of the different KLF15 mutants on β-catenin/TCF transcription showed that only KLF15-ΔN45 was able to repress β-cat/TCF-induced luciferase in contrast to mutants lacking longer N-terminal regions as well as the C-terminus in NRC, SW480 and HEK293 (Fig 3A and B and Supporting Information Fig S1C). Thus, the required domain for β-catenin/TCF-transcriptional repression seems to be localized in the N-terminal fragment excluding the first 45 amino acids of the KLF15 protein. Our observations reveal that KLF15 requires both, a minimal N-terminal domain for binding β-catenin and NLK as well as a C-terminal domain for TCF binding and nuclear translocation to achieve β-catenin/TCF transcriptional repression (summarized in Fig 3E).

KLF15 inhibition on β-catenin-LEF/TCF-transcription did not affect β-catenin localization or protein levels (Fig 3C), therefore, we hypothesized that NLK and KLF15 affects TCF stability. We tested ubiquitination of TCF4 upon KLF15 overexpression in HEK293 cells. NLK co-expression was used as a positive control since NLK was shown to target TCF4 for ubiquitination (Ishitani et al, 1999). HA-TCF4 was immunoprecipitated from cytosolic lysates and detected with an anti-ubiquitin antibody. We observed comparable increased TCF4 ubiquitination in both KLF15/TCF4 and NLK/TCF4 expressing cells. In contrast, ubiquitination in cells expressing TCF4 alone was comparable with cells transfected with the empty vector (EV; Fig 3D). These findings show that KLF15 promotes TCF4 proteasomal degradation.

### KLF15 regulates Wnt/β-catenin activation in cardiac tissue *in vivo*

**Figure 4:**
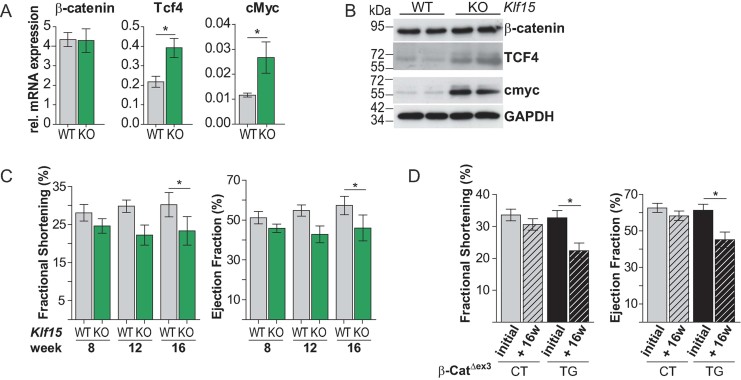
KLF15 regulates Wnt/β-catenin activity in cardiac tissue *in vivo* Data represent mean ± SEM; two-tailed Student's *t*-test, *p < 0.05. Unchanged mRNA expression of β-catenin but significantly increased expression of its target genes cMyc and Tcf4 in *Klf15* KO *versus* WT adult heart.Same observation as shown in **A** at the protein level.Fractional shortening (FS) and ejection fraction (EF) showing initial mild cardiac functional deterioration, which was aggravated with age in *Klf15* KO *versus* WT mice (*n* = 10).16 weeks of β-catenin stabilization resulted in a significant decrease in FS and EF in mice with cardiac specific β-catenin stabilization (β-cat^Δex3^).

Since we found that KLF15 regulates β-catenin-dependent transcription *in vitro*, we tested the hypothesis that this regulation takes place in the adult heart. Therefore, we analyzed a *Klf15* functional knock-out mouse model (*Klf15* KO), carrying an out-of-frame insertion of a partial lacZ cassette replacing exon 2 of the KLF15 coding region (Supporting Information Fig S2A). *Klf15* KO mice were viable and fertile and showed no apparent defects at baseline. Quantitative real time (qRT)-PCR analysis of cardiac tissue demonstrated no Klf15 mRNA expression in *Klf15* KO mice (Supporting Information Fig S2B). Histological analysis of liver, kidney, and lung up to 6 months of age showed no apparent morphological defects (Supporting Information Fig S2C). KLF15 exerted inhibition of β-catenin transcription, thus an opposite effect was expected upon KLF15 deletion. Indeed, analysis of cardiac tissue of 16-week-old mice revealed that β-catenin expression remained unchanged, but its target genes Tcf4 and cMyc were significantly increased at the mRNA (*p* < 0.05; Fig 4A) and protein level (Fig 4B) in *Klf15* KO *versus* wild-type (WT) mice. Endogenous ubiquitination of TCF4 was significantly decreased in *Klf15* KO (Supporting information Fig S3) complementing the *in vitro* finding showing increased TCF4 ubiquitination upon KLF15 overexpression. These observations show an *in vivo* cardiac de-repression of β-catenin/TCF-transcriptional activity in absence of KLF15.

Analysis of cardiac function assessed by ejection fraction (EF) and fractional shortening (FS) showed significant reduction in left ventricular (LV) function at 16 weeks of age in *Klf15* KO compared to WT at baseline (*p* < 0.05; Fig 4C). Neither wall thickness nor end-diastolic dimension of the LV (LVEDd) or cardiac mass showed significant differences between *Klf15* KO and WT mice (Supporting Information Table S1A). Since β-catenin/TCF-transcriptional activity was increased in the adult heart of *Klf15* KO mice, we were interested in the cardiac phenotype of mice exhibiting a direct cardiac β-catenin upregulation. We previously described a mouse model with cardiac inducible β-catenin stabilization (β-cat^Δex3^) showing worse cardiac function compared with controls after AngiotensinII (AngII)-induced hypertrophy (Baurand et al, 2007). Now, we analyzed cardiac function of β-cat^Δex3^ mice 16 weeks following induction of β-catenin stabilization. Similar to the functional phenotype observed in *Klf15* KO mice, β-cat^Δex3^ mice showed a significant decrease in EF and FS (Fig 4D). No differences were observed in wall thickness and/or cardiac mass between β-cat^Δex3^ and control mice (Supporting Information Table S1B). These observations suggest that KLF15-dependent regulation of β-catenin-dependent transcription is important to maintain normal cardiac homeostasis.

To discern the cause of the observed phenotype we investigated the expression of foetal genes A-type natriuretic peptide (ANP), B-type natriuretic peptide (BNP), GATA4 and connective tissue growth factor (CTGF) by qRT-PCR since it was shown that KLF15 regulates hypertrophic signalling and fibrosis (Fisch et al, 2007; Wang et al, 2008). No significant differences were observed between KO and WT mice with respect to cardiac hypertrophy, fibrosis as well as apoptosis (Supporting Information Fig S4A-D). Our data strongly suggest that regulation of hypertrophy and fibrosis via KLF15 occurs exclusively upon cardiac stress and are not the explanation for baseline cardiac deterioration.

### KLF15 controls cardiac progenitor cell fate in the adult murine heart

**Figure 5:**
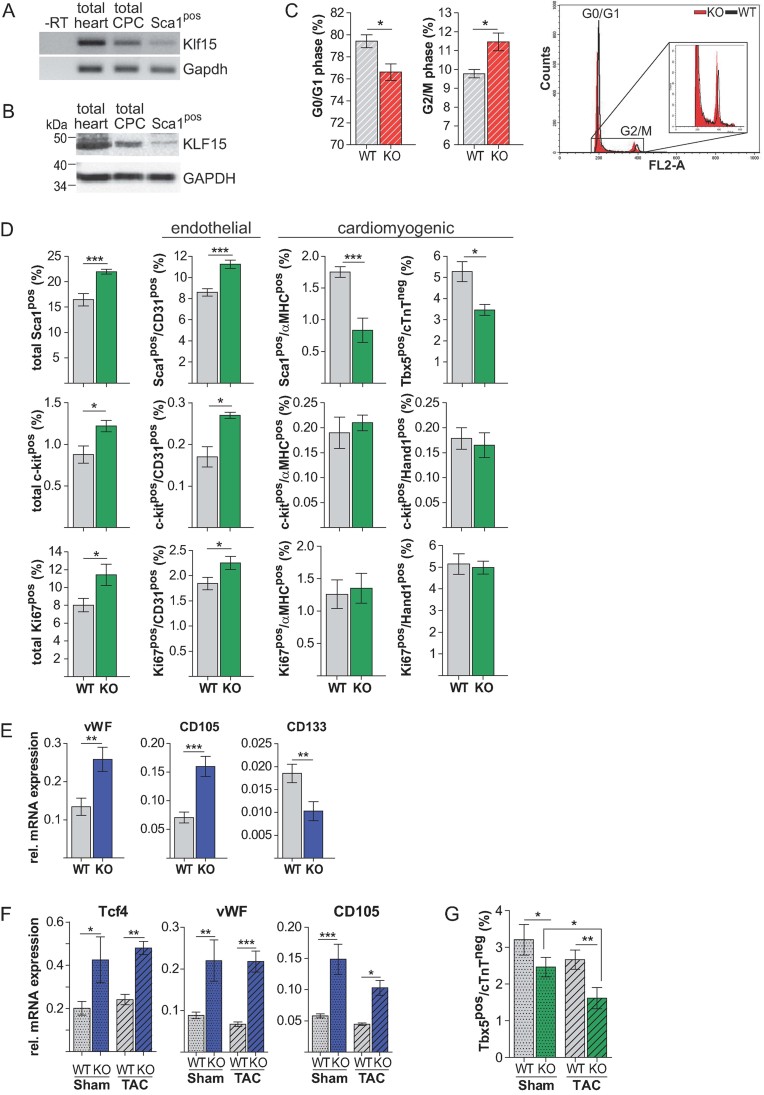
KLF15 controls cardiac progenitor cell fate in the postnatal heart Data represent mean ± SEM, (**C**) to (**E**) two-tailed Student's *t*-test; (**F**) and (**G**) ANOVA; Bonferroni's multiple comparison test, *p < 0.05, **p < 0.01, ***p < 0.001. QRT-PCR analysis.Western blot analysis showed KLF15 expression in total CPCs and more specific in Sca1^pos^ sub-population. Whole heart, containing all different cell populations, was used as control for KLF15 expression, as expected the expression is most robust in this fraction. RT was used as control for the RT-PCR.Cell cycle analysis shows significantly reduced cell number in G0/G1 but upregulation in G2/M phase in isolated CPCs of *Klf15* KO *versus* WT (*n* ≥ 8).FACS analysis of isolated adult CPCs revealed regulation of Sca1^pos^ and c-kit^pos^ population upon KLF15 depletion. Significant increase of total Sca1^pos^ and c-kit^pos^ cells was observed along with increased endothelial cell pool (Sca1^pos^/CD31^pos^, c-kit^pos^/CD31^pos^) in *Klf15* KO *versus* WT mice at baseline (*n* ≥ 9). In contrast, cells of the cardiomyogenic lineage (Sca1^pos^/αMHC^pos^, Tbx5^pos^/cTnT^neg^) were significantly reduced in *Klf15* KO *versus* WT mice. The c-kit cardiomyogenic lineage (c-kit^pos^/Hand1^pos^, c-kit^pos^/αMHC^pos^) were unchanged. Total and endothelial (Ki67^pos^/CD31^pos^) proliferating CPCs were increased while proliferation of cardiomyogenic cells was unchanged (αMHC^pos^, Hand1^pos^, co-stained with Ki67^pos^) in *Klf15* KO mice (*p* < 0.05).QRT-PCR analysis of total cardiac tissue resulted in enhanced expression of endothelial markers vWF and CD105, and reduced CD133 expression in *Klf15* KO *versus* WT mice.Two weeks after TAC upregulation of TCF4 and increased endothelial marker (vWF and CD105) expression in *Klf15* KO *versus* WT was confirmed by qRT-PCR analysis of cardiac tissue.Further decrease of the cardiomyogenic CPCs (Tbx5^pos^/cTnT^neg^) population as shown by FACS analysis in TAC-operated *Klf15* KO in comparison to sham-KO (*n* > 8).

Since we previously demonstrated that downregulation of β-catenin enhanced differentiation of Sca1-derived cardiomyogenic progenitor cells post-infarction (Zelarayan et al, 2008), we tested the hypothesis that KLF15-dependent regulation of β-catenin transcription affects CPC homeostasis. Expression of KLF15 was confirmed in the total CPCs as well as in the Sca1^pos^ sub-population as demonstrated by qRT-PCR and Western blot (Fig 5A and B). Whole heart lysate was used as control. The non-cardiomyocyte cell fraction containing CPCs isolated from 16-week-old *Klf15* KO and WT were analyzed by flow cytometry. The cell-cycle analysis showed a decreased cell number in G0/G1 resting phase and an increased cell number of cells in G2/M phase indicative of an increased number of dividing progenitors in *Klf15* KO mice (*p* < 0.05; Fig 5C). Furthermore, we investigated specific CPCs sub-populations previously described in the adult heart. The total number of Sca1^pos^ and c-kit^pos^ sub-populations were significantly increased in KO mice (*p* < 0.001 and <0.05; Fig 5D), although it is important to mention that the c-kit population was very low in both WT and *Klf15* KO mice. Next, we analyzed these CPC sub-populations in combination with endothelial and cardiomyogenic markers to identify progenitor cells in transition to different lineages (see Supporting Information Fig S5 for controls). Cells co-expressing Sca1 or c-kit and the endothelial cell marker CD31 were significantly increased in *Klf15* KO mice (*p* < 0.001 and <0.05; Fig 5D). In contrast, we observed a significant reduction of Sca1^pos^/αMHC^pos^ as well as the Tbx5^pos^/cTnT^neg^ cardiomyogenic populations in *Klf15* KO in comparison to WT mice (*p* < 0.001 and <0.05, respectively). No significant changes were detected in the double populations labelled by c-kit and the cardiomyogenic markers αMHC and Hand1 (Fig 5D).

While the total (Ki67^pos^) and endothelial proliferating cell fraction (Ki67^pos^/CD31^pos^) was significantly increased in *Klf15* KO mice (*p* < 0.05), the cardiomyogenic proliferative cells, Ki67^pos^/αMHC^pos^, and Ki67^pos^/Hand1^pos^, were unchanged (Fig 5D). Moreover, we detected increased mRNA expression of endothelial markers von Willebrand Factor (vWF) (*p* < 0.001) and Endoglin/CD105 (*p* < 0.01). Expression of CD133, which is known to be lost upon endothelial cell differentiation, was significantly reduced (*p* < 0.01) in cardiac tissue of *Klf15* KO *versus* WT mice (Fig 5E). Together these data show an increased progenitor cell fraction, which exhibits an endothelial phenotype in the *Klf15* KO mice.

Next, we were interested in the effect of KLF15 depletion in CPCs upon cardiac remodelling. Therefore, we applied a mild stimulus to induce hypertrophy by AngII and a more stringent stimulus by transverse aortic constriction (TAC) in 12-week-old *Klf15* KO and WT mice. In line with previous functional studies, 2 weeks following AngII treatment, *Klf15* KO showed accentuated cavity dilation as shown by LV diameter measurement (*p* < 0.01; Supporting Information Fig S6A and Table S2A). FACS analysis revealed that the Sca1^pos^/αMHC^pos^ as well as the Tbx5^pos^/cTnT^neg^ populations were significantly decreased in *Klf15* KO *versus* WT mice (*p* < 0.01) upon AngII infusion (Supporting Information Fig S6B). Two weeks following TAC, stronger dilation of the LV and accentuated cardiac dysfunction along with increased mortality was observed in *Klf15* KO in comparison to WT mice (*p* < 0.01; Supporting Information Fig S6C-D and Table S2B). Upregulation of TCF4 mRNA expression (*p* < 0.01; Fig 5F), reduction of the cardiomyogenic Tbx5^pos^/cTnT^neg^ population (*p* < 0.05; Fig 5G) and significant upregulation of vWF and CD105 expression (*p* < 0.001 and <0.05, respectively; Fig 5F) were confirmed in *Klf15* KO mice upon TAC. Altogether these data indicate that loss of KLF15 induces an activation of β-catenin-dependent transcription and this reduces the cardiomyogenic progenitor cell pool both in the normal and stressed adult heart.

### Endothelial identity of Sca1^pos^ CPCs in *Klf15* KO mice

**Figure 6:**
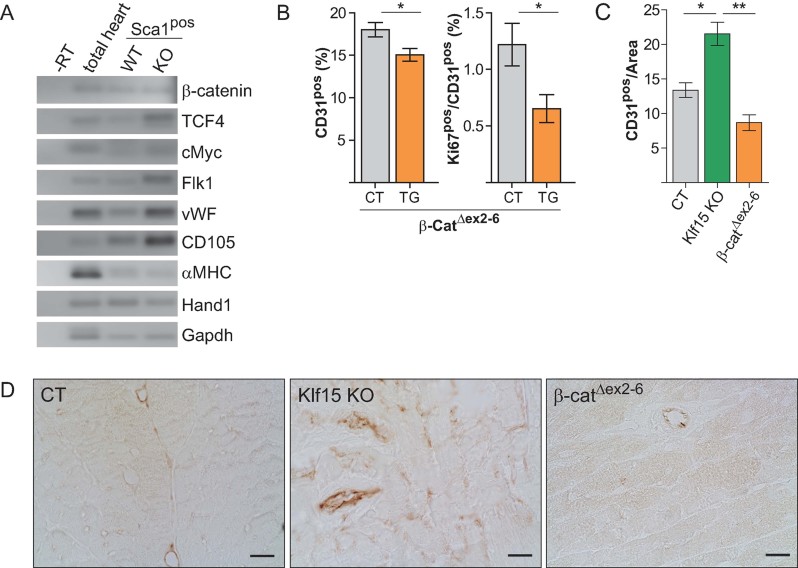
Klf15 deletion and β-catenin downregulation results in opposite regulation of endothelial CPC fate Data represent mean ± SEM; (**B**): two-tailed Student's *t*-test; (**C**): ANOVA; Bonferroni's multiple comparison test, *p < 0.05, **p < 0.01. QRT-PCR analysis of MACS-enriched Sca1^pos^ cells from adult hearts confirming unchanged β-catenin expression but upregulation of its target genes and endothelial markers in *Klf15* KO *versus* WT. cDNA from total adult heart and –RT were used as controls.FACS analysis of a mouse model with adult cardiac depleted β-catenin (β-cat^Δex2–6^) showed significantly reduced endothelial progenitor lineage (CD31^pos^), in contrast to the upregulation observed in KLF15 KO mice.Semi-quantitative immunohistochemistry analysis, confirming the observations in **B**.Representative pictures of the CD31 immunoperoxidase staining of adult cardiac tissue of control, *Klf15* KO, and β-cat^Δex2-6^ mice shown in **C**. Scale bar: 20 µm.

Since we found expression of KLF15 in Sca1^pos^ cells, we used this marker to assess gene regulation analysis in a purified cardiac progenitor population. The cardiac Sca1^pos^ cells were purified from *Klf15* KO and WT mice by magnetic cell sorting (MACS). 96.4% purity of sorted Sca1^pos^ cells was confirmed by flow cytometry. Analysis of the Wnt/β-catenin pathway by qRT-PCR showed upregulation of the β-catenin target genes cMyc and TCF4 in the purified Sca1 cell population. The early mesodermal haemangioblast marker Flk1 as well as the endothelial markers vWF, CD105 were upregulated while the cardiomyogenic markers αMHC and Hand1 were lower expressed in *Klf15* KO mice in comparison to WT Sca1^pos^ cells (Fig 6A). These data further support the role of KLF15 in CPC cell fate determination in the adult heart.

### KLF15 deletion directs CPCs to acquire an endothelial phenotype via activation of β-catenin dependent transcription

Loss of KLF15 results in upregulation of β-catenin transcription and promotes an endothelial phenotype of CPCs. In contrast, we previously described that cardiac inducible deletion of β-catenin (β-cat^Δex2–6^) enhanced cardiomyogenic CPC differentiation (Zelarayan et al, 2008). Now, we reasoned that β-catenin transcriptional inactivation would lead to the opposite regulation of endothelial fate of CPCs in comparison to KLF15. Therefore, we analyzed the CPC fraction in β-cat^Δex2–6^ by FACS and found indeed a significant reduction of CD31^pos^ as well as Ki67^pos^/CD31^pos^ CPCs in comparison to control mice (Fig 6B). This observation was confirmed by a semi-quantitative immunohistochemistry analysis, which shows enhanced CD31 positive areas in *Klf15* KO in comparison to control mice and moreover, a significant reduction of in β-cat^Δex2–6^ (Fig 6C and D).

**Figure 7:**
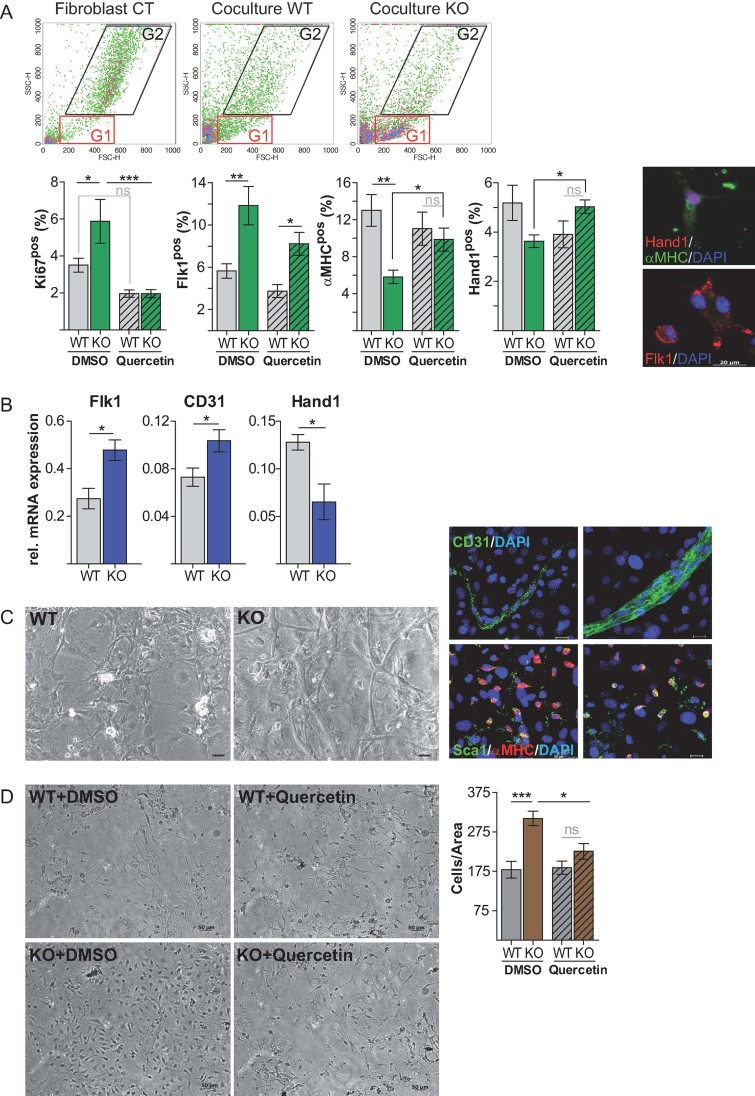
CPCs of *Klf15* KO acquire an endothelial phenotype via activation of β-catenin-dependent transcription Data represent mean ± SEM; (**B**) two-tailed Student's *t*-test; (**A**) and (**D**) ANOVA; Bonferroni's multiple comparison test, *p < 0.05, **p < 0.01, ***p < 0.001; ns: non-significant; scale bar in **A** 20 µm; in **C** and **D** 50 µm. *Klf15* KO *versus* WT cells showed an augmented cell population in the scatter plots gate G1. FACS analyses show increased Flk1^pos^ and Ki67^pos^ cells and decreased cardiac αMHC^pos^ and Hand1^pos^ populations in *Klf15* KO cultured CPCs. This was reversed by blocking β-catenin transcription with 5 µmol/L Quercetin (*n* = 6). Representative immunofluorescence pictures of co-cultures are shown.QRT-PCR analysis of CPCs 10 days in co-culture confirming the significant upregulation of Flk1 as well as downregulation of Hand1 expression. Expression of CD31 was found increased in *Klf15* KO *versus* WT cultured CPCs.Endothelial tube-like networks observed in 15-day *Klf15* KO cultured CPCs; representative immunofluorescence pictures showing expression of CD31 and Sca1/αMHC.Semi-quantitative analysis and representative pictures resulted in an increased cell number in *Klf15* KO *versus* WT mice, which was reversed upon Quercetin treatment.

To further elucidate the mechanism of KLF15 to drive CPC cell fate determination we established a co-culture system with adult cardiac fibroblasts as feeder layer for the enriched adult CPCs. The same cell number of cardiovascular progenitors from *Klf15* WT and KO mice were plated and cultured on arrested adult cardiac fibroblasts. Following 10 days co-culture an augmented cell population was detected in the *Klf15* KO cultured cells in comparison to WT cells when analyzed by flow cytometry (Gate G1; Fig 7A). Similar to our *in vivo* finding, we found a highly proliferative Ki67^pos^ cell fraction (*p* < 0.05) along with a decrease in αMHC^pos^ (*p* < 0.01) and Hand1^pos^ expressing cells. Interestingly, cells expressing Flk1 were augmented (*p* < 0.01; Fig 7A). Along with this finding, mRNA expression of Flk1 and CD31 was increased, whereas expression of Hand1 was decreased following 10 days culture in *Klf15* KO *versus* WT CPCs (*p* < 0.05; Fig 7B). An increased endothelial-like cell formation expressing CD31 was observed in *Klf15* KO cells in comparison to WT (Fig 7C).

To verify that the preferentially endothelial fate of *Klf15* KO CPCs is mediated by activation of β-catenin transcription, we performed a co-culture rescue experiment where CPCs were treated with 5 µmol/L Quercetin, a known β-catenin transcriptional inhibitor (Park et al, 2005). Quercetin was able to reverse the β-catenin transcriptional activation *in vitro* as demonstrated by luciferase gene reporter assay in HEK293 cells and attenuation of β-catenin target gene activation in co-cultured CPCs (Supporting Information Fig S7A and B). FACS analysis after 10 days co-culture showed that the significant increase of Flk1 in *Klf15* KO CPCs was partially reversed upon Quercetin treatment in comparison to the DMSO-treated *Klf15* KO CPCs. Moreover, the increase in cells expressing Ki67 as well as the reduction of αMHC^pos^ and Hand1^pos^ CPCs observed in *Klf15* KO CPCs was completely abrogated (Fig 7A and D). The significantly increased cell number in *Klf15* KO CPCs as well as the significantly decreased proliferation in Quercetin treated *Klf15* KO CPCs was confirmed via semi-quantification analysis following of 10 days culture (Fig 7D). Quercetin treatment did not affect WT CPC cell number.

Collectively, these data show that regulation of β-catenin transcriptional activity mediates regulation of cardiovascular cell fate downstream KLF15 in the postnatal heart.

## DISCUSSION

To the best of our knowledge, we provide the first evidence that KLF15 specifically interacts with components of the Wnt/β-catenin pathway and inhibits β-catenin/TCF-dependent transcriptional activation in cardiac cells. Moreover, our findings show that KLF15 controls CPCs via regulation of β-catenin transcription, which contributes to normal cardiac homeostasis.

### Cardiac interaction and regulation of the Wnt/β-catenin pathway by KLF15

In this study, we found KLF15 to specifically interact with β-catenin and TCF4 and to inhibit β-catenin/TCF-dependent transcription in cardiac cells. In line with the fact that the KLF proteins recruit co-factors to regulate transcription (Kaczynski et al, 2003) we found KLF15 to interact with NLK, an inhibitor of the β-catenin-LEF/TCF complex. We showed that a minimal KLF15 N-terminal domain is essential for both protein interaction and inhibition, which is consistent with the structure of KLF proteins containing a transcriptional activation or repression domain at the N-terminal region (Kaczynski et al, 2003). We showed that the first 45 amino acids are essential for KLF15-β-catenin interaction. Moreover, the binding domain for NLK as well as the repression domain is located between amino acids 45 and 152 of the KLF15 N-terminus indicating an important role of this interaction for transcriptional repression. Thus, different KLF15 N-terminal domains are required for binding both β-catenin and NLK suggesting that two different interaction domains are present at the N-terminus probably allowing for simultaneous binding to both β-catenin and NLK. We also demonstrated that KLF15 C-terminus is essential for binding TCF4 and for transcriptional repression, which may be explained by the requirement of the C-terminal domain for nuclear localization. This is in line with the identification of a putative nuclear localization signal for KLF15 at amino acid 369 (Gray et al, 2002). Mechanistically, we found that KLF15 targets TCF4 to ubiquitination, which was previously shown for NLK (Ishitani et al, 2003). Our data suggest that KLF15 may recruit NLK to the β-catenin/TCF-complex to prevent transcriptional activation by targeting TCF4 for proteasomal degradation.

We demonstrate that several N-terminal mutants of KLF15 were able to translocate into the nucleus but were insufficient to inhibit β-catenin/TCF4 transcription. These data show that KLF15 requires both the C-terminus for nuclear translocation and a minimal N-terminal regulatory region to exert inhibition on β-catenin/TCF transcriptional activation. Similarly, another KLF member, KLF4, was shown to inhibit β-catenin function by preventing β-catenin binding to transcriptional mediators (Zhang et al, 2006). These findings suggest specific members of the KLF family proteins to allow tissue-specific inhibition of the globally expressed Wnt/β-catenin pathway and render KLF15 as an important cardiac regulator of β-catenin/TCF transcription.

### KLF15 regulates cardiac β-catenin transcription and is important for cardiac homeostasis

Our *in vitro* finding showing KLF15-dependent β-catenin/TCF transcriptional regulation prompted us to investigate this regulation *in vivo* using a *Klf15* KO mouse. Deletion of KLF15 resulted in an initial mild cardiac functional deterioration, which was aggravated with aging. Cardiac activation of β-catenin/TCF-dependent transcription was observed in *Klf15* KO mouse. It is consistent with our *in vitro* results showing inhibition of β-catenin transcription by KLF15, indicating that no compensation for KLF15 concerning Wnt transcriptional regulation takes place in the heart. Previous studies demonstrated a role of KLF15 in regulating hypertrophic signalling and fibrosis of the adult heart (Fisch et al, 2007; Haldar et al, 2010; Leenders et al, 2010; Wang et al, 2008). Similar to these functional observations, we confirmed exacerbated reduced LV function in response to TAC along with cardiac hypertrophy and fibrosis as well as accentuated LV dilation after AngII infusion in *Klf15* KO mice. However, at baseline we did not find neither activation of hypertrophic pathways, nor increased fibrosis in *Klf15* KO mice. Our data show that the previously identified KLF15-dependent mechanisms regulating hypertrophy and fibrosis upon induced cardiac hypertrophy are not activated in absence of any stimuli, suggesting an alternative mechanism through which KLF15 controls normal cardiac homeostasis.

### KLF15 regulates CPC fate in the postnatal heart

We previously described increased cardiomyogenic differentiation of the sub-population of Sca1^pos^ cells upon β-catenin depletion in the postnatal heart (Zelarayan et al, 2008). In this study, we demonstrated expression of KLF15 not only in total CPCs but also specifically in the Sca1^pos^ sub-population. Therefore, we reasoned that KLF15-dependent regulation of β-catenin transcription affects CPC biology. CPCs from *Klf15* KO were augmented in the proliferative cell cycle phase G2 and specifically the Sca1^pos^ and c-kit^pos^ CPC sub-populations showed increased proliferation in *Klf15* KO *versus* WT. This might be explained by an enhanced β-catenin transcriptional activation as described in several studies showing that Wnt/β-catenin signalling is required for proliferation and expansion of specified cardiovascular progenitors during development (Gessert & Kuhl, 2010). Due to the lack of specific markers for different CPC populations, we combined stem cell markers with markers of early and mature lineages to identify stem cells in transition to cardiomyogenic (Hand1, Tbx5, αMHC) and mesodermal/endothelial cells (Flk1, CD31). *Klf15* KO mice showed a significant reduction of the cardiomyogenic progenitors in contrast to an increase of endothelial progenitors in the Sca1^pos^ CPC pool *in vivo* and *in vitro*. Moreover, an early upregulation of cells expressing the mesodermal/haemangioblastic marker Flk1 followed by an increase in CD31 expression was detected *in vitro*. These finding suggest an endothelial preference of *Klf15* KO CPCs further supported by an increased expression of a combination of endothelial markers including vWF and CD105, the latter commonly used to trace endothelial differentiation. In parallel we observed a reduction of CD133, which is expressed in undifferentiated progenitors of the endothelial lineages and becomes lower during its differentiation (Shmelkov et al, 2005).

Further substantiating these data, the isolated Sca1^pos^ sub-population showed upregulation of endothelial markers in *Klf15* KO mice suggesting an activation of an endothelial differentiation gene program. Recently, the Sca1^pos^/CD31^pos^ cardiac side population cells were described as progenitors of cardiac endothelial cells (Liang et al, 2011). Similar to our observations, they showed vascular tube-like network formation along with upregulation of CD133 in undifferentiated state and upregulation of vWF during differentiation.

Our analysis showed an increase in the c-kit^pos^ CPC as well as the c-kit^pos^ endothelial fraction upon KLF15 deletion, although the number of cells was very low and no changes in the cardiomyogenic pool were observed. These observations might be explained by the fact that the adult c-kit population was shown not to play a role in generating cardiomyogenic cells in the adult heart (Zaruba et al, 2010).

Imbalance of the cardiomyogenic and endothelial CPCs in *Klf15* KO mice was also observed upon mild and stringent induced stress, which indicates that KLF15-dependent control of CPCs plays also a role during stress induced remodelling. In conclusion, KLF15 *loss-of-function* favours the expansion and commitment of CPCs to endothelial cells thus regulating the compartment of CPCs in the postnatal heart, which has critical implications in the control of cardiac homeostasis. Similarly, pathways involved in early CPCs specification were shown to control homeostasis of the normal and injured heart (Boni et al, 2008).

### KLF15 regulates CPC fate via regulation of β-catenin transcription

Our study clearly shows a β-catenin/TCF activated transcription upon KLF15 deletion *in vivo*, which results in a preferentially endothelial CPC phenotype. In contrast, we previously showed that cardiac β-catenin deletion promoted CPC differentiation towards cardiomyogenic cells (Zelarayan et al, 2008). Here, we further showed that mice with cardiac β-catenin depletion exhibit the opposite phenotype as observed in *Klf15* KO, namely a reduction of endothelial-like CPCs *in vivo*. As expected, CPCs from mice with a cardiac stabilization of β-catenin showed a decreased cardiomyogenic CPC differentiation *in vitro* (Zelarayan et al, 2008) similarly to *Klf15* KO CPCs. These findings strongly indicate that KLF15 controls CPCs homeostasis in the adult heart via regulating β-catenin/TCF transcription.

To further test the hypothesis that β-catenin transcriptional activation mediates CPC regulation downstream KLF15, we blocked β-catenin transcription in CPCs using a pharmacological approach via Quercetin. Confirming our hypothesis, this treatment reversed the increase in CPC proliferation, Flk1 cells and endothelial markers. Moreover, the reduction of cardiomyogenic cells was observed specifically in Quercetin treated-*Klf15* KO *versus* DMSO-*Klf15* KO. Quercetin treatment of WT CPCs did not affect endothelial or cardiomyogenic CPCs, while the slight reduction observed in proliferation can be explained by the fact that Wnt/β-catenin transcription is also attenuated in the WT CPCs. Together these data indicate that β-catenin transcription downstream KLF15 is critical for CPC homeostasis. This is supported by the fact that similar to the cardiac phenotype observed in *Klf15* KO, mice with a long term induction of cardiac β-catenin stabilization exhibited reduced LV function. Other studies showed that stimulation of β-catenin transcription leads to cardiac impairment and accelerated myocardial remodelling (Malekar et al, 2010).

It is important to mention that during early development, two mesodermal Flk1^pos^ cell populations are defined. An early mesodermal haemangioblastic cell population expressing Flk1 but not the multi-potent cardiac marker Mesp1 develops into endothelial cells and the blood lineage. A later mesodermal Flk1 population co-expressing Mesp1 results in a progenitor pool for the all the cardiovascular lineage (Gessert & Kuhl, 2010; Kouskoff et al, 2005). Expression of Flk1 is sequentially lost in the cardiomyogenic population while it expression persists in the endothelial lineages. Active Wnt/β-catenin signalling activates these mesodermal progenitors to become haemangioblasts and inactivation of Wnt/β-catenin through Notch will allow them to become cardiomyogenic cells (Chen et al, 2008; Gessert & Kuhl, 2010; Naito et al, 2006; Ueno, 2007). Similarly, our *in vivo* and *in vitro* data showed that KLF15 deletion leads to an early upregulation of mesodermal haemangioblastic cell marker Flk1, which leads to a final upregulation in the endothelial CD31 population by regulating activation of the Wnt/β-catenin signalling. Likewise, β-catenin maintains normal homeostasis of progenitor pools in the gut (Pinto & Clevers, 2005) and nervous system (Zechner et al, 2003).

**Figure 8:**
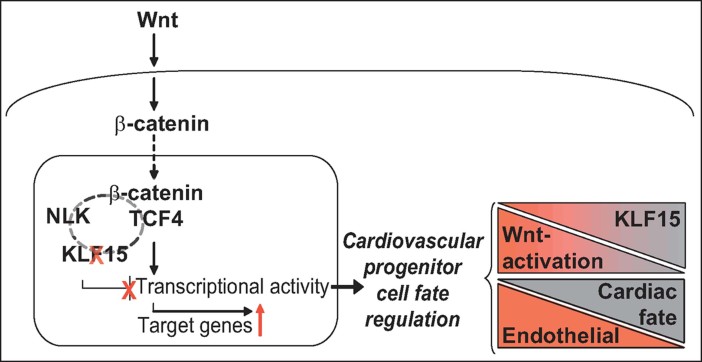
Hypothetical *in vivo* role of KLF15 and Wnt/β-catenin crosstalk in the adult heart Interactions among KLF15, NLK and the nuclear β-catenin/TCF-complex repress canonical Wnt signalling to maintain a balance of cardiovascular committed progenitor cell pool and a normal cardiac homeostasis. Loss of KLF15 leads to an upregulation of β-catenin/TCF-dependent transcription and displaces cardiovascular progenitors to acquire an endothelial rather than a cardiac identity.

We conclude that β-catenin transcriptional activation in the absence of KLF15 favours an increased endothelial cell while repressing cardiac myogenic differentiation. KLF15 is essential to maintain the balance of early differentiating CPCs, which may have critical implications in the control of heart homeostasis and its adaptation to pathologic states (Fig 8). These observations allow us to conclude that an altered CPC homeostasis might be another cellular mechanism aside cardiac hypertrophy that contributes to enhanced cardiac deterioration upon deletion of KLF15. Both scenarios are not mutually exclusive, and may contribute to heart failure development. Importantly, KLF15 is expressed in human adult cardiac tissue and similar to the finding in mouse, is known to be downregulated in the failing heart (Fisch et al, 2007; Haldar et al, 2010). Our study reveals an interesting regulation that could be exploited to activate human CPCs upon cardiac remodelling.

In summary, our present study shows a novel and important level of regulation of endogenous CPC specification, which may play a significant role in the regeneration of the injured heart. Our data provide an insight into the molecular mechanisms driving CPC biology by demonstrating the potential of KLF15 to modulate CPC cell fate via regulation of the Wnt/β-catenin signalling. It makes this pathway a suitable target for therapeutic approaches to treat pathological cardiovascular adaptation.

## MATERIALS AND METHODS

### Mouse strains

The KLF15 *loss-of-function* mouse (*Klf15* KO) was achieved by an out-of-frame insertion of a lacZ cassette in exon 2 of the KLF15 coding region. The *Klf15* KO mouse was kindly provided by Dr. HJ Schaeffer/Prof. W Birchmeier. Inducible, cardiac specific β-catenin gain-of-function (αMHC-CrePR1/β-Cat^Δex3^) was achieved by flanking exon 3 with loxP sites of the β-catenin coding region. Heart-specific expression of the Cre recombinase under control of the αMHC promoter was activated by administration of the estrogen derivate mifepristone (RU486, 30 mg/kg body weight/day; Sigma–Aldrich) i.p. for 5 days. Excision of exon 3 results in a non-degradable mutant of β-catenin (Baurand et al, 2007).

Inducible, cardiac specific β-catenin loss-of-function (αMHC-MerCreMer/β-cat^Δex2–6^) was achieved by flanking exon 2–6 with loxP sites of the β-catenin gene. Heart-specific expression of the Cre recombinase under control of the αMHC promoter was activated by administration of 90 µl Tamoxifen (10 mg/ml; Sigma–Aldrich) i.p. for 4 days. Littermates negative for Cre recombinase were used as control. The experiments were approved by the Niedersachsen (AZ: 10/0230) animal review board.

### Cell culture

HEK293 cells were grown in DMEM and SW480 in RPMI both supplemented with 10% FCS/l-glutamine/antibiotics. NRC were isolated and cultured as described previously (El Jamali et al, 2004). For isolation of cardiac fibroblast, adult mice were anesthetized and hearts were dissected, minced and digested with collagenaseII/trypsin and cultivated in DMEM/F12 supplemented with 10% FCS/l-glutamine/antibiotics/100 µM ascorbic acid. For isolation of a non-cardiomyocyte cell fraction, adult mice were anesthetized and hearts were dissected, minced and filtered through 40 and 30 µm mesh. Cells were used directly for FACS staining or seeded on MitomycinC (Sigma–Aldrich)-arrested adult cardiac fibroblasts and cultivated for 10 days at 37°C, 5% CO_2_ in DMEM/F12 supplemented with 2% FCS/l-glutamine/antibiotics/100 µM ascorbic acid. For rescue experiments co-cultures were treated with 5 µmol/L Quercetin hydrate (Acros Organics) for 10 days; control cells were treated with the solvent DMSO.

### Co-immunoprecipitation and ubiquitination assays

HEK293 cells and NRC were transfected with expression plasmids for cmyc-β-catenin, HA-TCF4, HA-NLK, Flag-KLF15 (full length, -ΔN45, -ΔN152, -ΔN260, -ΔC) and pcDNA3.1. Cells were harvested 48 h after transfection, lysed with radio-immunoprecipitation assay (RIPA) buffer with protease inhibitors, and immunoprecipitated with antibodies against cmyc-(Santa Cruz), HA-, or Flag-tag (Sigma–Aldrich). Interacting proteins were detected by immunoblotting using the appropriate anti-tag-HRP antibodies (1:2000; Sigma–Aldrich). As controls, protein lysates transfected with EV and precipitation with IgG isotype control were used. Whole cell lysates were immunoblotted with respective antibodies to detected protein expression. Endogenous co-immunoprecipitation was performed with an antibody against KLF15 (Santa Cruz). Interacting proteins were detected by immunoblotting using the appropriate anti-β-catenin, -NLK and -TCF4 antibodies (1:1000). IgG isotype control was used as a co-immunoprecipitation control.

For ubiquitination assay RIPA buffer was supplemented with 10 µmol/L N-ethylmaleimide (NEM; Sigma–Aldrich). TCF4 was immunoprecipitated with anti-HA antibody (Sigma–Aldrich). Ubiquitination of TCF4 was analyzed by immunoblotting using anti-ubiquitin antibody (1:1000; Cell Signaling) and anti-rabbit-HRP secondary antibody (1:10,000; Dako).

### Luciferase reporter assay

HEK293, SW480 cells, and NRC were co-transfected with plasmids expressing non-degradable β-catenin (β-cat-ΔN) or TCF4 and various amounts of full-length and truncated constructs of KLF15 (250 pg–250 ng) along with the pTOPflash luciferase reporter (750 ng) and *Renilla* luciferase expressing plasmid (5 ng) as a normalizing control. pFOPflash containing mutated TCF binding sites was used as negative reporter control. Luciferase activity was determined using Dual-Luciferase Reporter Assay (Promega) 48 h after transfection, according to manufacturer's instructions.

### Flow cytometry analysis

The non-cardiomyocyte population was prepared as mentioned above. Cells were fixed in 1% formaldehyde/PBS, permeabilized in FACS buffer containing 0.5% Saponin (Sigma–Aldrich) and stained with anti-CD31-FITC, -Flk1-PE, -Sca1-FITC, c-kit-APC (1:200; eBioscience) anti-αMHC, -Tbx5, -cTnT (1:500; Abcam); -Ki67-PE, and -Hand1 (1:200; Santa Cruz). The cells were subsequently stained with anti-rabbit IgG-APC or anti-mouse F(ab)_2_-FITC (1:500; Jackson ImmunoResearch). Respective isotype controls were used. For cell cycle analysis the isolated cells were fixed in cold 70% ethanol, treated with 100 µg/ml RNase and incubated with 50 µg/ml propidium iodide. Fluorescence signals were detected with a FACS-Calibur flow cytometer (Becton Dickinson).

**The paper explained**

PROBLEM:

Heart failure is characterized by an imbalanced myocardial damage and repair. Unfortunately, the therapeutic modalities to prevent heart failure are currently limited. It is now well accepted that regeneration events occur in the adult mammalian hearts during normal aging and disease. Although low levels of cardiomyocyte renewal are detectable in the adult heart, promising pharmaceutical or genetic therapy approaches could be developed to enhance this effect. Therefore, the elucidation of cardiac progenitor cell contribution to cardiac homeostasis, aging and injury along with the molecular signalling directing these events is essential to help to promote postnatal cardiac regeneration. Thus, we need to focus our efforts to explore these processes to benefit from endogenous mechanisms and to be able to exploit them therapeutically. Wnt/β-catenin pathway is essential for embryonic cardiogenesis and for normal cardiac homeostasis and remodelling. Therefore, this pathway is a good candidate to be investigated in endogenous CPC biology.

RESULTS:

The present study provides a detailed biochemical and functional analysis of a novel interaction between a member of the KLF family (KLF15) and Wnt-pathway components, which leads to repression of the Wnt transcriptional activity. We clearly show the presence of two important domains within the KLF15 protein sequence, a minimal N-terminal region and the C-terminus, which are essential for β-catenin/TCF4 transcriptional repression in cardiac cells. Moreover, using a genetic model we report the role of KLF15 in the regulation of the CPC biology in the postnatal heart *in vivo* and *in vitro*. *Klf15* knockout mice exhibit a de-repression of β-catenin/TCF4 transcription. This shifts the cell fate of cardiovascular progenitors towards an endothelial lineage antagonizing cardiogenic cell formation in the healthy and stressed heart, similar to what has been observed in embryonic development. Moreover, the endothelial cell fate shift can be reversed upon inhibition of the Wnt activation *in vitro*, which demonstrated that KLF15 controls CPCs cell fate via regulation of β-catenin transcription.

IMPACT:

This work may have identified a novel molecular switch to therapeutically modulate CPC fate and unlock the regenerative potential of the adult heart. It might help to prevent massive cardiac loss upon injury. Specifically, our observations highlight the role of KLF15 in controlling the early specification of the progenitor pool to specifically generate different cardiovascular cellular subtypes in the postnatal heart. This observation is of relevance given the fact that KLF15 is expressed in the human heart and is known to be dysregulated upon prohypertrophic stimuli. The present data help us to understand the biological processes leading to formation of cardiovascular cells from resident precursors, which may have critical implications in the control of heart homeostasis and its adaptation to pathologic states.

### Magnetic activated cell sorting

The non-cardiomyocyte population was prepared by mincing and 1 mg/ml collagenase II digestion, stained with anti-Sca1-FITC (1:10; Miltenyi Biotec) and labelled with anti-FITC-MicroBeads (1:5; Miltenyi Biotec). Sca1^pos^ cells were enriched by two rounds of MS Columns (Miltenyi Biotec) separation. Purity was checked by FACS analysis. Sca1^pos^ cells were directly applied to mRNA extraction.

### RNA isolation, reverse transcription and quantitative real-time PCR analysis

Total RNA was isolated from cardiac mouse tissue using the NucleoSpin RNA II kit (Macherey-Nagel), according to manufacturer's instruction and treated with DNase. cDNA was synthesized with Oligo(dT)_20_ primer and M-MLV reverse transcriptase (Promega). Quantitative real-time PCR analyses were performed with SYBR Green (Qiagen) on an iCycler instrument (BioRad). Copy numbers were calculated using the iCycler software with a relative standard curve obtained using the log dilutions of cDNA of gene of interest. All reactions were run in triplicates and normalized to both reference control genes Gapdh and β-actin applying the geometric mean calculation. Primers are listed in Supporting Information Table S3.

### Immunocytochemistry

HEK293 and NRC were grown on poly-l-lysin or 1% gelatine coated coverslips, respectively; fixed in 4% PFA, permeabilized in 0.1% TritonX-100 and blocked in 1% BSA for 1 h at RT. Cells were incubated with respective antibodies against β-catenin (BD Bioscience), NLK (Bethyl Laboratories), KLF15 (Abcam), HA-tag (Sigma–Aldrich) and Flag-tag (Sigma–Aldrich) at 1:200 dilutions O/N at 4°C.

Cardiac progenitor cells were grown on fibroblast on gelatine-coated coverslips. Cells were blocked in 2% FCS; surface markers were detected with anti-Flk1 (1:200; eBioscience), anti-CD31-FITC (1:200; eBioscience) for 1 h at 4°C. Next, cells were fixed in 1% PFA, permeabilized in 0.3% TritonX-100/0.2% BSA and blocked in 0.1% TritonX-100/5% BSA for 1 h at RT. Intracellular staining was performed with antibodies against Hand1 (1:100; Santa Cruz) and O/N at 4°C.

AlexaFluor488 and AlexaFluor594 conjugated secondary antibodies (1:200; Invitrogen) were used for immunofluorescence labelling. For nuclei visualization cells were incubated with Hoechst33342 (Sigma–Aldrich) for 5 min and mounted with ProlongGold (Invitrogen). Microscopic images were captured with a confocal microscope (710, Zeiss).

### Histological analysis

Heart, liver, lung and kidney were rinsed in PBS, fixed in 4% PFA, embedded in paraffin and sectioned at 3 µm. Sections were de-paraffinized and used for the different stainings. Liver, lung and kidney were stained with Weigert's haematoxylin/Eosin. For visualization of cardiac fibrosis sections were stained with Weigert's iron haematoxylin staining and Accustain-Trichrome according to manufacturer's instructions for Masson-Trichrome staining (Sigma–Aldrich), and with DirectRed80 1 h (Sigma–Aldrich) for Sirius Red staining. Detection of apoptosis was performed with indirect TUNEL ApopTag Red *in situ* Apoptosis Detection Kit (Chemicon) according to manufacturer's instruction. To assess the cross-sectional area (CSA) of cardiomyocytes sections were stained with 20 mg/ml lectin wheat germ agglutinin (WGA) FITC (Sigma–Aldrich) and mounted with ProlongGold (Invitrogen), random fields were photographed and 150 cells were counted to calculate CSA using semiautomatic AxioVision software (Zeiss). Microscopic images were captured with a digital microscope (IX70, Olympus).

Immunohistochemistry was performed in de-paraffinized heart sections. Slides were microwaved for antigen retrieval in citrate buffer and endogenous peroxides were blocked. Slides were incubated with a rabbit anti-CD31 (1:50; Abcam) O/N, then washed in PBS and incubated with a secondary goat anti-rabbit IgG (1:200; Sigma–Aldrich) 1 h at 37°C. After additional washing in PBS preparations were incubated 1 h at 37°C in a rabbit peroxidase-antiperoxidase complex (Sigma–Aldrich), subsequently washed and bound peroxidase was revealed by incubation with 3,3′-diaminobenzidine tetrahydrochloride (DAB, 0.05% in PBS). Microscopic images were captured with a digital microscope (M2, Zeiss) and the staining was semi-quantified using ImageJ software.

### Echocardiographic analysis

Mice were anesthetized by 2.4% isoflurane inhalation and ventricular measurements were done with a VisualSonics Vevo 2100 Imaging System equipped with a 45 MHz MS-550D MicroScan transducer. The observer was unaware of the genotypes and treatments.

### Statistical analysis

Differences between experimental groups were analyzed using two-tailed Student's *t*-test or ANOVA test followed by Bonferroni's multiple comparison test. Data are presented as mean ± SEM. *p* < 0.05 values were considered significant.

## Supplementary material

Detailed facts of importance to specialist readers are published as ”Supporting Information”. Such documents are peer-reviewed, but not copy-edited or typeset. They are made available as submitted by the authors.
