# Supplementary material for: Krueppel-like factor 15 regulates Wnt/β-catenin transcription and controls cardiac progenitor cell fate in the postnatal heart
Source: EMBO Mol Med. 2012 Jul 5;4(9):992–1007. doi: 10.1002/emmm.201101043 (PMC3491830; doi:10.1002/emmm.201101043)
Supplement: Supplementary file 1 [file emmm0004-0992-SD1.pdf]

EMBO
Molecular Medicine

Manuscript EMM-2011-01043

Krueppel-like factor 15 regulates Wnt/β-catenin transcription and controls cardiac progenitor cell fate in the postnatal heart

Claudia Noack, Maria-Patapia Zafiriou, Hans-Jörg Schäffer, Anke Renger, Elena Pavlova, Dietz Rainer, Wolfram H. Zimmermann, Martin W. Bergmann and Laura C. Zelarayán

Corresponding author: Laura Zelarayan, Georg-August-University Goettingen

Review timeline:

Submission date:	28 October 2011
Editorial Decision:	12 December 2011
Rebuttal:	28 December 2011
Editorial Decision:	04 January 2012
Revision received:	04 April 2012
Editorial Decision:	08 May 2012
Revision received:	21 May 2012
Accepted:	24 May 2012

Transaction Report:

(Note: With the exception of the correction of typographical or spelling errors that could be a source of ambiguity, letters and reports are not edited. The original formatting of letters and referee reports may not be reflected in this compilation.)

1st Editorial Decision 12 December 2011

Thank you for the submission of your manuscript to our editorial offices. We have now received feedback from 2 out of 3 reviewers whom we asked to evaluate your manuscript. Given that both reviewers provide very similar recommendations, we prefer to make a decision now in order to avoid further delay in the process.

As you will see from the enclosed reports, while the reviewers find some observations intriguing and potentially interesting, they also raised serious issues regarding the physiological significance. In addition they also feel that given some conceptual and technical shortcomings, your conclusions appear to not be fully supported by the data.

Therefore, in light of such significant reservations and the fact that EMBO Molecular Medicine can only afford to accept papers which receive enthusiastic support from a majority of referees, I am afraid I see little choice but to return the manuscript to you at this point with the decision that we cannot offer to publish it.

Thank you for the opportunity to consider this manuscript. I am sorry we cannot be more positive on this occasion, but we hope nevertheless that you will find our referees' comments helpful.

Yours sincerely,

Editor
EMBO Molecular Medicine

***** Reviewer's comments *****

Referee #1:

In this manuscript, Noack et al. demonstrated that Krueppel-like factor 15 (Klf 15) interacts with β-catenin, T-cell factor 4 and NEMO-like kinase, thereby inhibiting β-catenin dependent transcriptional activation. They also showed that Klf15 negatively regulates β-catenin downstream targets. They observed an increase in endothelial cell markers in the Sca1+ sorted cardiac progenitor cells from Klf15 KO mice and claimed that Klf15 controls differentiation of cardiac progenitor cells (CPCs) towards endothelial cells.

The conclusion regarding the function of Klf15 in cardiac progenitor cell fate is not well justified. Although the observation of CPC fate switch is intriguing, this conclusion is weakened by the lack of in vivo cell fate analysis and lineage tracing studies. The interactions of Klf15 with components of the Wnt/beta-catenin pathway were mainly carried out by protein overexpression in vitro, and the authors should present data for endogenous protein interactions to strengthen their conclusions. The present study contradicts published data that Klf15 KO mice show eccentric cardiac hypertrophy, and that Klf15 can inhibit GATA4 and myocyte enhancer factor 2 (MEF2) function. It is not clear how to reconcile this discrepancy. Given Klf15 KO may affect many cell types during heart development, a conditional KO strategy should be carried out to determine the cell type specific function of Klf15 in adult CPCs. These major issues need to be addressed to support their conclusion.

Specific comments:

1. The authors show that Klf15 interacts with β-catenin by overexpressing two genes in cell culture. This interaction should be confirmed endogenously in NRC without overexpressing the genes.

2. Figure 2D: By overexpressing Klf15 and Nlk, ubiquitination of TCF4 is increased. Ubiquitination of endogenous TCF4 by Klf15 should be performed in NRC.

3. In Figure 4C, cardiac function was measured by ejection fraction. The difference between WT and KLF15 is quite subtle, fraction shortening should also be measured.

4. Figure 5 shows that KLF15 controls cardiac progenitor cell fate in isolated Sca1 positive cells. Lineage tracing should be performed to examine whether Klf 15 expressing cells lead to an endothelial lineage. Since part of Klf15 coding region is replaced with the lacZ gene, β-gal positive cells should be examined in adult Klf5 KO heart. Also, use of Sca 1 as the only CPC marker in this study is not sufficient, and other cardiac progenitor markers such as c-kit and Islet 1 should be tested with the CPC cells as well.

5. Figure 7: If the Sca1 positive CPC cells are proliferating, why do the authors perform the co-culture with fibroblast cells? Immunostaining of cardiac and endothelial markers should be performed on the Sca1 positive CPC cells from the WT and KLF15 KO mice in 7C. Quantification of cell numbers should be performed in 7D.

6. Increased endothelial lineage of CPC in Klf15 KO mice needs to be confirmed in vivo by immunostaining of CD31 on heart sections from control and β-catenin ex2-6 mice.

7. Figure 2A: KLF15 represses luciferase activity on the control vector, does it mean there is a nonspecific sequence on the vector independent of the TCF binding site?

8. Figure 5: Sca1 negative cells should be included as a negative control for the RT-PCR. If there is a decrease in the Sca1pos/ MHCpos cells in 5C, why there is no change in MHCpos/Ki67pos cells in 5D?

9. Figure C, the summary of different KLF15 mutant binding to TCF needs to be aligned

Referee #3:

Noack et al. investigated a role for KLF15 in the control of regulatory interactions between beta-catenin, NLK, and TCF4. In addition, the investigators addressed a possible functional role for KLF15 in the heart and in cardiac progenitor cells (CPCs) in response to aging and cardiac stress. While prior data demonstrated a role of KLF15 as a repressor of pathological cardiac hypertrophy and fibrosis, Noack et al. provide novel evidence that KLF15 interacts with and regulates beta-catenin activity and localization through associations with NLK and TCF4. In addition to this novel interaction, the authors demonstrate a role for KLF15 in determining CPC cell outcome, where deletion of KLF15 drives CPCs toward an endothelial cell fate via increased beta-catenin transcriptional activity. In keeping with previous findings, Noack et al. further demonstrate that KLF15 KO animals are more susceptible to angiotensin II (Ang II) and transverse aortic constriction (TAC)-induced cardiac stress. However, their interpretation that KLF15 plays a role in age-related heart deterioration is weakly supported by a single figure (Fig.4C), and not supported by supplementary data (Fig.S3). Collectively, the authors provide fairly convincing evidence to support their interpretation that KLF15 regulates beta-catenin in order to maintain cardiac homeostasis. Nonetheless, in its current form, the manuscript is unsuitable for publication in EMBO Molecular Medicine.

Specific Points:

1. While the authors provide convincing evidence for KLF15 in the interaction with beta-catenin, NLK, and TCF4 in HEK293 cells and neonatal rat cardiomyocytes (NRCs), these data are generated from ectopic overexpression of the factors. Thus, we do not know if endogenous interactions also occur. Demonstration of endogenous interactions is necessary to support the interpretation that KLF15 mediates cardiac homeostasis through association with beta-catenin.

2. A co-culture model is employed to demonstrate a role for KLF15 in controlling CPC cell fate. A bioactive food component, quercetin, was used to inhibit beta-catenin in KLF15 KO cells to examine CPC phenotype. However, quercetin has many activities that have effects on cellular redox state, inflammation and proliferation, and thus it is not possible to conclude that the effects are due to suppression of beta-catenin activity. Only one figure (Fig.7E) uses beta-catenin deletion to address CPC phenotype, and this occurs in the presence of KLF15. While in vivo findings support a role for KLF15 in CPC phenotype, further in vitro, mechanistic data would have strengthened the manuscript.

3. In the Results section the authors mention that NRCs are co-transfected with c-myc-beta-catenin and FLAG-KLF15-full length or N-terminal truncated constructs, and that the data are shown in Fig.1A. While HEK293 appear to show this, the NRCs only show KLF15-beta-catenin. Several discrepancies between the text and the figures were noted throughout the manuscript.

4. The authors mention that KLF15-full length interaction with beta-catenin, NLK, and TCF4 were confirmed in NRCs and that these data could be found in Fig.S1A. However, Fig.S1A contains information regarding HEK293 cells. It is difficult to discern why the authors focus on HEK293 cells in Fig.1 and then jump to NRCs for Fig.2A, to SW480 for Fig.2B, and back to HEK293s for Fig.2C and D. A lack of consistency with cell types makes the results difficult to interpret.

5. In Fig. 3A, why does the localization pattern of KLF15-N260 and KLF15/beta-cat/DAPI merged with two cells looking distinctly different (i.e. nuclear vs. cytosolic)? In addition, Fig.3C needs correction.

6. In Fig.4C, the authors state that cardiac function declined significantly at 12 weeks of age in KLF15 KO animals, yet EF% does not show significance in the figure.

Rebuttal 28 December 2011

Thanks for your reply to our Manuscript EMM-2011-01043 submitted on 28.10.2011.

I understand your decision, which is based on the opinion of 2 different reviewers. However, I am afraid I cannot fully agree with the comments of the reviewers, which I would like to discuss point by point in the attached document.

I would really appreciate if you can take a bit of time to have a look to our detailed discussion to clarify some, what we believe, misinterpretations.

Moreover, I would also like to ask for the comments of the 3rd missing reviewer if it is possible. As you mention the reviewers find our observations intriguing and potentially interesting, I feel like most of the conceptual and technical shortcomings could be discussed and full filled in a short while since they are no major experiments and many of them ongoing. We strongly believe on the relevance of our study and that the basic molecular as wells as the physiological impact fits the best in EMBO mol med as we talked with some experts in the field. Thus, I would really appreciate if there is a possibility to re-consider the decision and let us full fill the questions/concerns remarked by the reviewers.

Please do not hesitate to contact me if you have any questions.
I strongly appreciate your time and disposition and I hope you can give us a favorable answer.

Best regards,
Dr. Laura Zelarayan

Point-by-Point-Response:

Referee#1:

"In this manuscript, Noack et al. demonstrated that Krueppel-like factor 15 (Klf 15) interacts with β-catenin, T-cell factor 4 and NEMO-like kinase, thereby inhibiting β-catenin dependent transcriptional activation. They also showed that Klf15 negatively regulates β-catenin downstream targets. They observed an increase in endothelial cell markers in the Sca1+ sorted cardiac progenitor cells from Klf15 KO mice and claimed that Klf15 controls differentiation of cardiac progenitor cells (CPCs) towards endothelial cells.

The conclusion regarding the function of Klf15 in cardiac progenitor cell fate is not well justified. Although the observation of CPC fate switch is intriguing, this conclusion is weakened by the lack of in vivo cell fate analysis and lineage tracing studies. The interactions of Klf15 with components of the Wnt/beta-catenin pathway were mainly carried out by protein overexpression in vitro, and the authors should present data for endogenous protein interactions to strengthen their conclusions".

We would like to mention that we have performed a cell fate analysis in vitro and in vivo (Fig. 5 and 7), however without lineage tracing (discussed below).

Concerning the endogenous protein interactions we can provide the data if the reviewers/editor consider it important.

"The present study contradicts published data that Klf15 KO mice show eccentric cardiac hypertrophy, and that Klf15 can inhibit GATA4 and myocyte enhancer factor 2 (MEF2) function. It is not clear how to reconcile this discrepancy".

It should be noted that our study does not really contradict previous findings. The study of Fisch et al (Fisch, Gray et al. 2007) showed evidences of KLF15 as a negative regulator of cardiac hypertrophy in part through inhibition of MEF2 and GATA4 transcriptional pathways. These conclusions were made based on the analysis of KLF15 knockout (-/-) mice followed induced hypertrophy via aortic constriction. Nevertheless, the study did not cover the analysis of cardiac

progenitor cells at baseline upon KLF15 loss-of-function in vivo, which is the main and the novel observation in our study.

Additionally, the present study covers the analysis of cardiac progenitor cells in KLF15 KO mice under chronic AngII-induced hypertrophy and hemodynamic stress induced by transverse aortic constriction (TAC). Similar to the observations made by Fisch et al. we observed reduced fractional shortening at baseline in 12- to 16-week-old KLF15 functional knockout (KO) mice, although we did not observe increased left ventricular cavity size, which may be explained by mouse strain differences. The study of Fisch et al reported no significant difference between KLF15 (+/+) and (-/-) myocyte area at baseline, which fits with our echocardiographic data indicating no difference in wall thickness between KLF15 KO and control mice. Moreover, we investigated apoptosis via TUNEL assay in 12- to 16-week-old KLF15 KO mice (Figure S3 and Table S2) and no difference was detected when compared with the control mice, which is in line with the TUNEL staining, immunohistochemistry for cleaved caspase-3 and apoptotic gene expression analysis preformed by Fisch et al. However, we do not find evidence of cardiac hypertrophy and/or fibrosis to be the main mechanism as indicated by myocyte area, echocardiographic showing no difference in wall thickness and measurement of hypertrophic and fibrosis pathway activation in KLF15 KO in comparison to control at baseline.

In summary, our study complements previous findings concerning the role of KLF15 in normal cardiac homeostasis at baseline and in the stressed heart concerning the balance of endogenous progenitor cell populations.

"Given Klf15 KO may affect many cell types during heart development, a conditional KO strategy should be carried out to determine the cell type specific function of Klf15 in adult CPCs. These major issues need to be addressed to support their conclusion."

Expression of KLF15 was previously documented after postnatal day 30 (Fisch, Gray et al. 2007). Therefore, the influence of KLF15 during heart development may not be as relevant as it is for the adult cardiovascular system.

Aiming to elucidate the cell type specific function of KLF15 we isolated the CPCs and analyzed them in vivo and vitro (as shown in Fig. 5 and 7 in Manuscript). Independent from the effect of KLF15 in cardiomyocytes and fibroblasts, this analysis clearly showed that KLF15 deletion controls CPC cell fate in a cell autonomous manner since acquisition of endothelial fate upon KLF15 deletion in vivo was also observed in vitro in isolated CPCs.

Specific comments:

1. The authors show that Klf15 interacts with β-catenin by overexpressing two genes in cell culture. This interaction should be confirmed endogenously in NRC without overexpressing the genes.

This data can be provided.

2. Figure 2D: By overexpressing Klf15 and Nlk, ubiquitination of TCF4 is increased. Ubiquitination of endogenous TCF4 by Klf15 should be performed in NRC.

This data are provided as a single experiment but a triplicate can be easily provided (attached Fig. II).

3. In Figure 4C, cardiac function was measured by ejection fraction. The difference between WT and KLF15 is quite subtle fraction shortening should also be measured.

We provide these data (attached Fig. III).

4. Figure 5 shows that KLF15 controls cardiac progenitor cell fate in isolated Sca1 positive cells. Lineage tracing should be performed to examine whether Klf 15 expressing cells lead to an endothelial lineage.

We fully agree that a lineage tracing study will further support and elucidate in detail the CPC type affected by KLF15 expression. Unfortunately, this study is currently very difficult to achieve due to the lack of a specific marker for adult CPCs. A study tracing the different CPC populations, indentified so far in the adult heart, would be an option but we honestly believe that this analysis is out of the scope of the present manuscript.

It should be noted that under the hypothesis that de-repression of b-catenin transcriptional activity via KLF15 deletion in a KLF15 KO background, CPCs are switched to an endothelial lineage. Therefore, KLF15 expressing CPCs would not be possible to trace in such a system. These cells would be expected to keep a normal cell homeostasis instead of leading to an endothelial fate in a wild-type background.

Since part of Klf15 coding region is replaced with the lacZ gene, β-gal positive cells should be examined in adult Klf5 KO heart.

Unfortunately, the inserted LacZ cassette is not functional, since it was only partly inserted to replace exon 1 and 2 of Klf15.

Also, use of Sca 1 as the only CPC marker in this study is not sufficient, and other cardiac progenitor markers such as c-kit and Islet 1 should be tested with the CPC cells as well.

The authors agree that cardiac Sca1 cells are not fully representative of CPCs in the adult heart. However, the percentage of these cells is more representative in the adult heart in comparison to the c-Kit or Islet1 populations previously described, at least in our hands. In contrast to the 16,46±1,23 % Sca1 cells that we observed in WT 12-weeks old mice, we found 1,23±1,10 % c-Kit cells and were not able to observe a clear population by analyzing the Islet1 cells by FACS analysis of the cardiomyocytes depleted fraction and by immunofluorescence analysis using different antibodies (attached Fig. IV). Moreover, Sca1 population is very well documented in several studies and described as the CPCs with the greatest potential for cardiomyogenic differentiation. They were shown to have the potential as stem cells to differentiate in vitro and may contribute to the regeneration of injured adult murine hearts (Matsuura et al, 2004; Oh et al, 2003; Pfister et al, 2005; Tateishi et al, 2007). The authors can provide the data with the c-Kit population.

5. Figure 7: If the Sca1 positive CPC cells are proliferating, why do the authors perform the co-culture with fibroblast cells?

The co-cultures are performed with adult cardiac fibroblast to mimic the endogenous cardiac environment. Fibroblasts are important cells supporting cardiac differentiation and homeostasis, which is independent from the proliferative capacity of the co-cultured cells. The use of conditional medium may add/remove components that are not known, making it difficult to control the quality and reproducibility of the medium (Li et al, 2005). It has been found that cells are maintained in tissue culture much more readily if they are supported on substrate components most closely resembling the extracellular matrix (ECM) in which they occurred in vivo (Baharvand et al, 2005). The matrix provided by fibroblasts was shown to be one of the best. Currently, several studies are switching from matrigel to extracellular ECMs from decellularized fibroblast, which is proposed to be essential for proliferation, growth and differentiation.

We also performed some analysis using matrigel instead of fibroblast as feeder layer but unfortunately cell differentiation was not optimal in that case. This is in line with the fact that matrigel inhibits the differentiation of cells due to all the different protein content and alteration of protein expression. The matrigel composition and structure was showed to inhibit TGF-b2 gene expression (LaGier et al, 2007).

Immunostaining of cardiac and endothelial markers should be performed on the Sca1 positive CPC cells from the WT and KLF15 KO mice in 7C. Quantification of cell numbers should be *performed in 7D.*

The mentioned co-immunostaining can be easily provided. We provided partially the required immunostaining (attached Fig. V). Please notice that the quantification of the representative picture in Fig. 7D is depicted for different cell types and total proliferative cells in

© EMBO

Fig. 7B using FACS analysis, which we consider a better quantification tool. A semi-quantification of Fig. 7D can be provided.

6. Increased endothelial lineage of CPC in Klf15 KO mice needs to be confirmed in vivo by immunostaining of CD31 on heart sections from control and β-cateninΔ ex2-6 mice.

7. Figure 2A: KLF15 represses luciferase activity on the control vector, does it mean there is a nonspecific sequence on the vector independent of the TCF binding site?

Although the FOPflash control vector contains 3 mutated Tcf/Lef binding elements upstream to the luciferase promoter, a minimal background expression is always detectable. Co-expression of the positive regulators β-catenin and TCF4 resulted not only in activation of the TOPflash system, but also in weak activity of its control vector in SW480, HEK293 and NRC. We used SW480 carcinoma cells since they are characterized by a constitutive active Wnt/β-catenin pathway. Co-expression of activating TCF4 with FOPflash resulted in a higher activation of the control system compared to HEK293 cells or NRC, showing that the control vector is able to respond slightly to present β-catenin/TCF. Accordingly, co-expression of KLF15 results in a weak inhibition of the control vector. Importantly, both observations were statistically not significant.

Moreover, to control for non-specific interactions we tested a Ras/MAPK-responsive luciferase reporter system. Co-transfection with the same KLF15 expressing plasmid showed no regulation (attached Fig. VII), indicating a specific inhibition of β-catenin/TCF-dependent transcription.

8. Figure 5: Sca1 negative cells should be included as a negative control for the RT-PCR. If there is a decrease in the Sca1pos/αMHCpos cells in 5C, why there is no change in αMHCpos/Ki67pos cells in 5D?

Our study demonstrated that the αMHC proliferating populations are not affected upon KLF15 deletion but the amount of Sca1/αMHC cardiac progenitor committed cells, which represent a specified cardiogenic CPC population. In another words, it indicates that the increased Sca1 cell population upon KLF15 depletion differentiates towards an endothelial fate decreasing the cardiogenic cell population (please refer to attached Fig. VIII for explanations and Fig. 5 in manuscript), which does not affect the total proliferative αMHC committed cells. It suggests a pool of uncommitted Sca1 cells, which differentiate towards a certain lineage according to the activated signaling.

9. Figure C, the summary of different KLF15 mutant binding to TCF needs to be aligned

We apologize and corrected this mistake.

Referee #3:

Noack et al. investigated a role for KLF15 in the control of regulatory interactions between beta-catenin, NLK, and TCF4. In addition, the investigators addressed a possible functional role for KLF15 in the heart and in cardiac progenitor cells (CPCs) in response to aging and cardiac stress. While prior data demonstrated a role of KLF15 as a repressor of pathological cardiac hypertrophy and fibrosis, Noack et al. provide novel evidence that KLF15 interacts with and regulates beta-catenin activity and localization through associations with NLK and TCF4. In addition to this novel interaction, the authors demonstrate a role for KLF15 in determining CPC cell outcome, where deletion of KLF15 drives CPCs toward an endothelial cell fate via increased beta-catenin transcriptional activity. In keeping with previous findings, Noack et al. further demonstrate that KLF15 KO animals are more susceptible to angiotensin II (Ang II) and transverse aortic constriction (TAC)-induced cardiac stress.
However, their interpretation that KLF15 plays a role in age-related heart deterioration is weakly supported by a single figure (Fig.4C), and not supported by supplementary data (Fig.S3).

Collectively, the authors provide fairly convincing evidence to support their interpretation that KLF15 regulates beta-catenin in order to maintain cardiac homeostasis. Nonetheless, in its current form, the manuscript is unsuitable for publication in EMBO Molecular Medicine.

Specific Points:
1. While the authors provide convincing evidence for KLF15 in the interaction with beta-catenin, NLK, and TCF4 in HEK293 cells and neonatal rat cardiomyocytes (NRCs), these data are generated from ectopic overexpression of the factors. Thus, we do not know if endogenous interactions also occur. Demonstration of endogenous interactions is necessary to support the interpretation that KLF15 mediates cardiac homeostasis through association with beta-catenin.

This data can be provided

2. A co-culture model is employed to demonstrate a role for KLF15 in controlling CPC cell fate. A bioactive food component, quercetin, was used to inhibit beta-catenin in KLF15 KO cells to examine CPC phenotype. However, quercetin has many activities that have effects on cellular redox state, inflammation and proliferation, and thus it is not possible to conclude that the effects are due to suppression of beta-catenin activity.

We tested the specificity of Quercetin in vitro to assure the conclusion of the rescue experiment. This assay demonstrated a downregulation of the β-catenin target gene c-Myc upon Quercetin treatment of co-cultured CPCs although β-catenin expression was unchanged, indicating that Quercetin specifically block β-catenin-dependent transcription as previously described (Park, Chang et al. 2005). This analysis was used to complement our in vivo analysis showing that cardiac downregulation of β-catenin in vivo leads to an opposite effect concerning the percentage of endothelial committed cells. An additional assay employing siRNA could be performed if the reviewers/editor consider it important.

Only one figure (Fig.7E) uses beta-catenin deletion to address CPC phenotype, and this occurs in the presence of KLF15. While in vivo findings support a role for KLF15 in CPC phenotype, further in vitro, mechanistic data would have strengthened the manuscript.

Analysis of a mouse model with a cardiac downregulation of β-catenin was used to test the hypothesis that β-catenin is the downstream mediator of KLF15, which finally controls CPC cell fate. This analysis confirmed the mentioned hypothesis and allows us to conclude that mechanistically KLF15 plays a role in CPC cell fate decision via regulation of β-catenin dependent transcription. The presence of KLF15 in the cardiac β-catenin loss-of-function models, which shows altered CPC cell fate regulation, indicates that KLF15 is upstream β-catenin.

3. In the Results section the authors mention that NRCs are co-transfected with c-myc-beta-catenin and FLAG-KLF15-full length or N-terminal truncated constructs, and that the data are shown in Fig.1A. While HEK293 appear to show this, the NRCs only show KLF15-beta-catenin. Several discrepancies between the text and the figures were noted throughout the manuscript.

4. The authors mention that KLF15-full length interaction with beta-catenin, NLK, and TCF4 were confirmed in NRCs and that these data could be found in Fig.S1A. However, Fig.S1A contains information regarding HEK293 cells. It is difficult to discern why the authors focus on HEK293 cells in Fig.1 and then jump to NRCs for Fig.2A, to SW480 for Fig.2B, and back to HEK293s for Fig.2C and D. A lack of consistency with cell types makes the results difficult to interpret.

3 and 4.

We apologize and corrected these discrepancies:

<u>Page 3:</u> *"For validation HEK293 cells and were co-transfected with both plasmids expressing cmyc-β-catenin and either a Flag-KLF15-full-length or an N-terminal truncated Flag-(ΔN45, ΔN152, ΔN260), lacking the indicated amino acids, as well as a C-terminal truncated KLF15-ΔC to*

identify the binding domain. KLF15-full-length interaction with β-catenin, NLK and TCF4 were confirmed in Neonatal rat cardiomyocytes (NRC)(Fig. 1).”

Page 4: *“…b-catenin/TCF4 and b-catenin/NLK known interactions were confirmed in Hek293 cells (Fig. S1A).”*

The explanation for the use of different cell types is the transfection efficiency of the different cells used in this study. While NRCs have a very low transfection efficiency, HEK cells showed better results. Therefore, we decided to show the detailed mutation analysis in a cell line and confirm all the interactions using primary NRC.

Moreover, SW480 cells were used to avoid a triple transfection, since SW480 cells are a tumor cell line that expresses a stabilized form of β-catenin.

In summary, HEK293 cells showed the interactions of different truncated forms of KLF15 with β-catenin, TCF4 and NLK. NRCs were used to confirm these interactions in a cardiac cell lineage and SW480 cells were only used for the reporter luciferase assay to show the effect of KLF15 on β-catenin/TCF transcriptional activity. The mutation analysis in NRCs could be provided if the reviewers/editor consider it of relevance.

5. In Fig. 3A, why does the localization pattern of KLF15-N260 and KLF15/beta-cat/DAPI merged with two cells looking distinctly different (i.e. nuclear vs. cytosolic)? In addition, Fig.3C needs correction.

The localization of KLF15-ΔN260 (red) and b-catenin (green) in Fig. 3A is nuclear. The immunofluorescence picture shows 2 cells: one of them is binucleated, which shows a more prominent expression of KLF15 and b-catenin in the nucleus and co-localized with the DAPI staining. The nuclear expression in the cell located in the left side of the picture is weaker. This may be due to endogenous expression in contrast to overexpression detected in the binucleated cell. Clear cytosolic expression is only observed in Fig. 3A bottom (KLF15-DC) as confirmed via Western blot in Fig. 3B. We can provide another immunofluorescence picture if it is confusing.

We apologize and corrected the mistake in Fig. 3C.

6. In Fig.4C, the authors state that cardiac function declined significantly at 12 weeks of age in KLF15 KO animals, yet EF% does not show significance in the figure.

We apologize and corrected the mistake.

Literature:

Baharvand H, Azarnia M, Parivar K, Ashtiani SK (2005) The effect of extracellular matrix on embryonic stem cell-derived cardiomyocytes. J Mol Cell Cardiol 38: 495-503

LaGier AJ, Yoo SH, Alfonso EC, Meiners S, Fini ME (2007) Inhibition of human corneal epithelial production of fibrotic mediator TGF-beta2 by basement membrane-like extracellular matrix. Invest Ophthalmol Vis Sci 48: 1061-1071

Li Y, Powell S, Brunette E, Lebkowski J, Mandalam R (2005) Expansion of human embryonic stem cells in defined serum-free medium devoid of animal-derived products. Biotechnol Bioeng 91: 688-698

Matsuura K, Nagai T, Nishigaki N, Oyama T, Nishi J, Wada H, Sano M, Toko H, Akazawa H, Sato T, Nakaya H, Kasanuki H, Komuro I (2004) Adult cardiac Sca-1-positive cells differentiate into beating cardiomyocytes. J Biol Chem 279: 11384-11391

Oh H, Bradfute SB, Gallardo TD, Nakamura T, Gaussin V, Mishina Y, Pocius J, Michael LH, Behringer RR, Garry DJ, Entman ML, Schneider MD (2003) Cardiac progenitor cells from adult

myocardium: Homing, differentiation, and fusion after infarction. Proc Natl Acad Sci U S A 100: 5834-5839

Pfister O, Mouquet F, Jain M, Summer R, Helmes M, Fine A, Colucci WS, Liao R (2005) CD31- but Not CD31+ Cardiac Side Population Cells Exhibit Functional Cardiomyogenic Differentiation. Circ Res: 01.RES.0000173297.0000153793.fa

Tateishi K, Ashihara E, Takehara N, Nomura T, Honsho S, Nakagami T, Morikawa S, Takahashi T, Ueyama T, Matsubara H, Oh H (2007) Clonally amplified cardiac stem cells are regulated by Sca-1 signaling for efficient cardiovascular regeneration. J Cell Sci 120: 1791-1800

Figure II

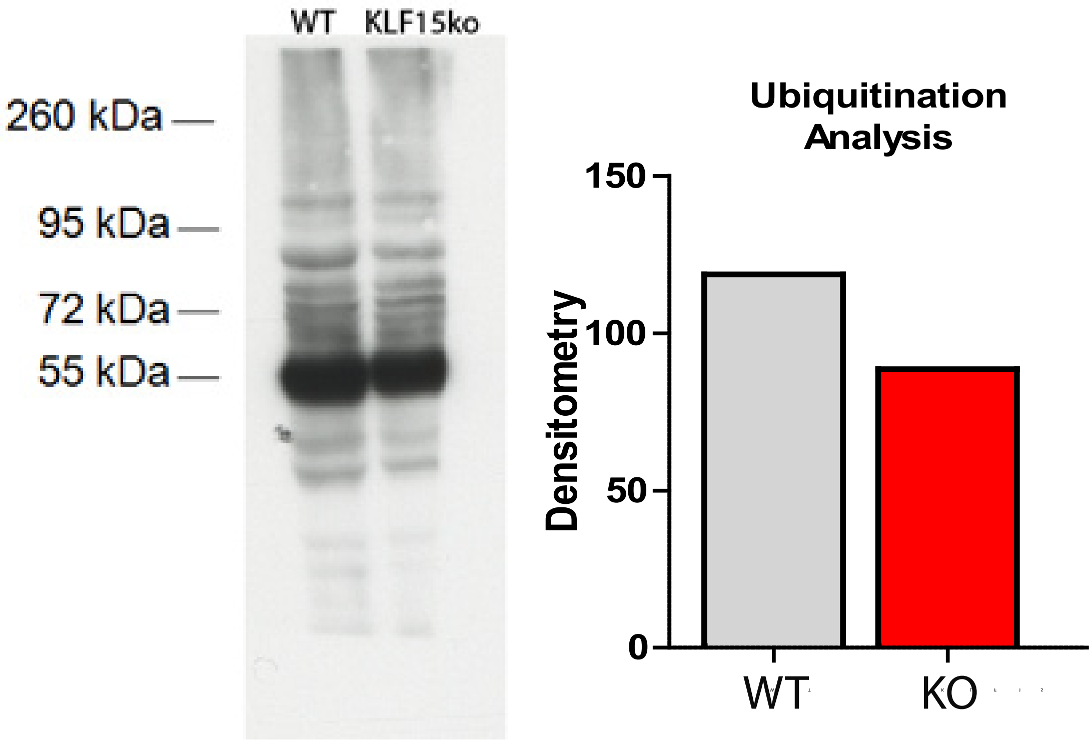

Ubiquitination analysis of adult cardiac tissue. A co-immunoprecipitation using a TCF4 antibody was performed and subsequently detection of ubiquitination using a ubiquitin antibody. Observed enhanced ubiquitination of TCF4 in KLF15 KO mice in comparison to WT mice.

Figure III

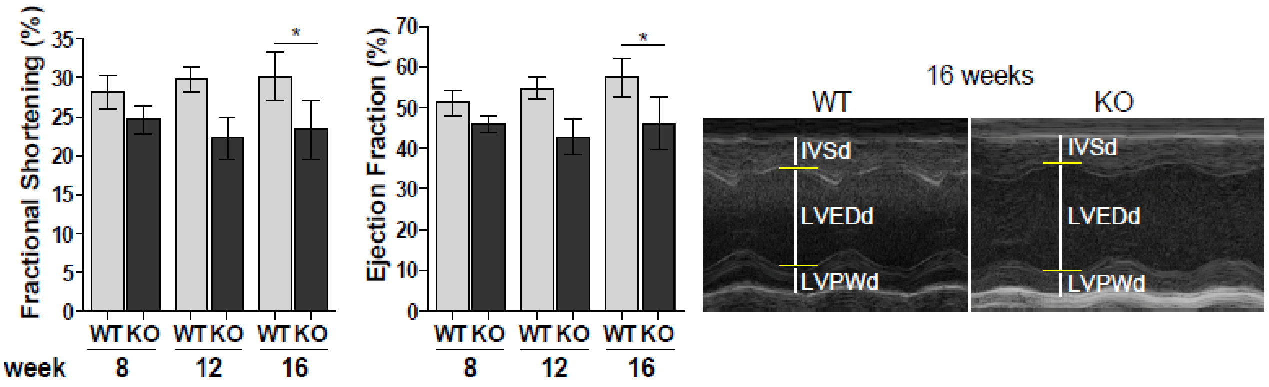

Echocardiography analysis of 8, 12, and 16-week-old Klf15 WT and KO mice revealed declined cardiac function demonstrated by ejection fraction (EF) and fractional shortening (FS), which became significant at 16 weeks in Klf15 KO vs. WT mice (n=10). Representative M-Mode pictures of Klf15 WT and KO mice at 16 weeks of age showing unaltered wall thickness. *IVS: interventricular septum, LVED: diameter of the left ventricle and LVPW: left ventricular posterior wall.*

Figure IV

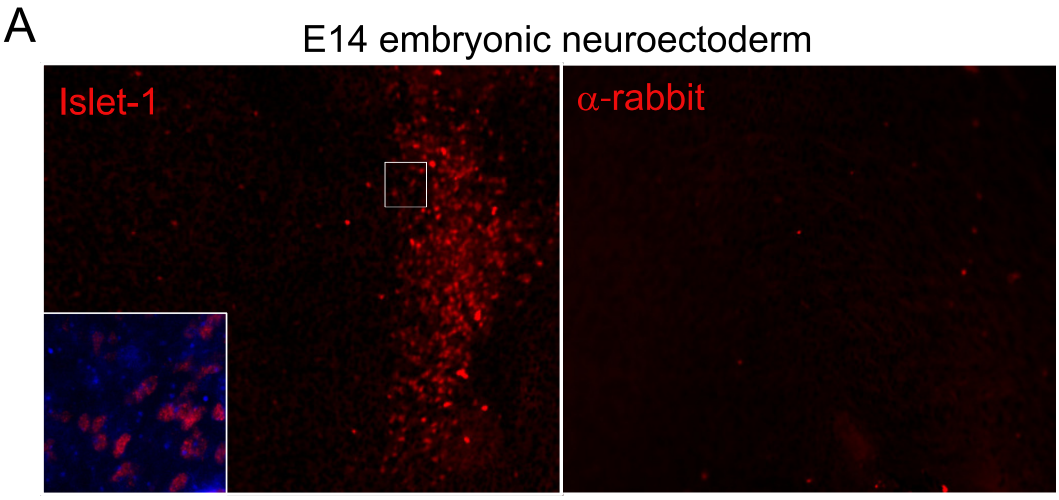

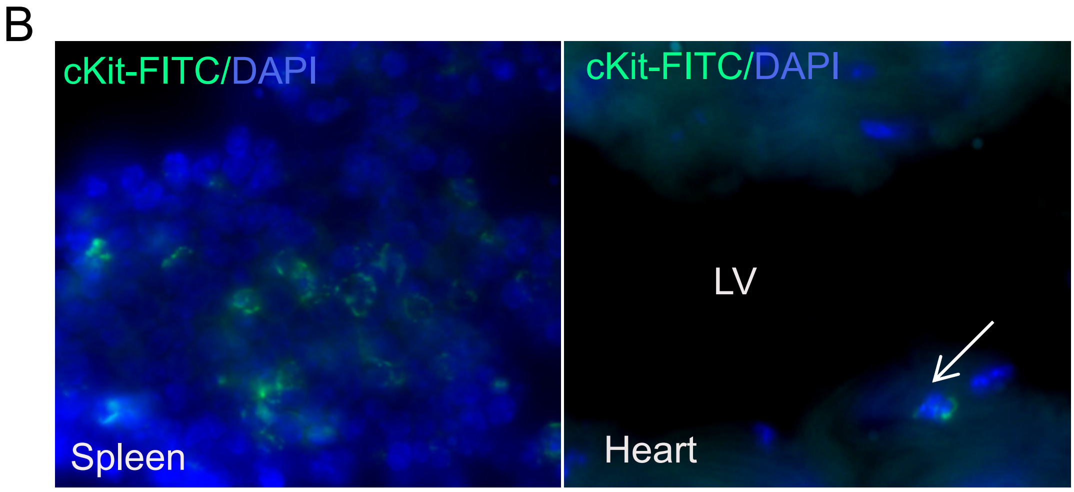

A. Detection of Islet-1 in embryonic tissue in paraffin sections. Islet-1 was detected in neuroectoderm tissue in E14 embryos used as control. The same antibody was used to detect Islet-1 in the adult heart but no staining was observed. **B**. Detection of c-Kit using a direct FITC-labelled antibody in cryosections. C-kit was observed in spleen, which was used as control. Few cells expressing c-kit were detected in the adult heart. *LV: left ventricle*

Figure V

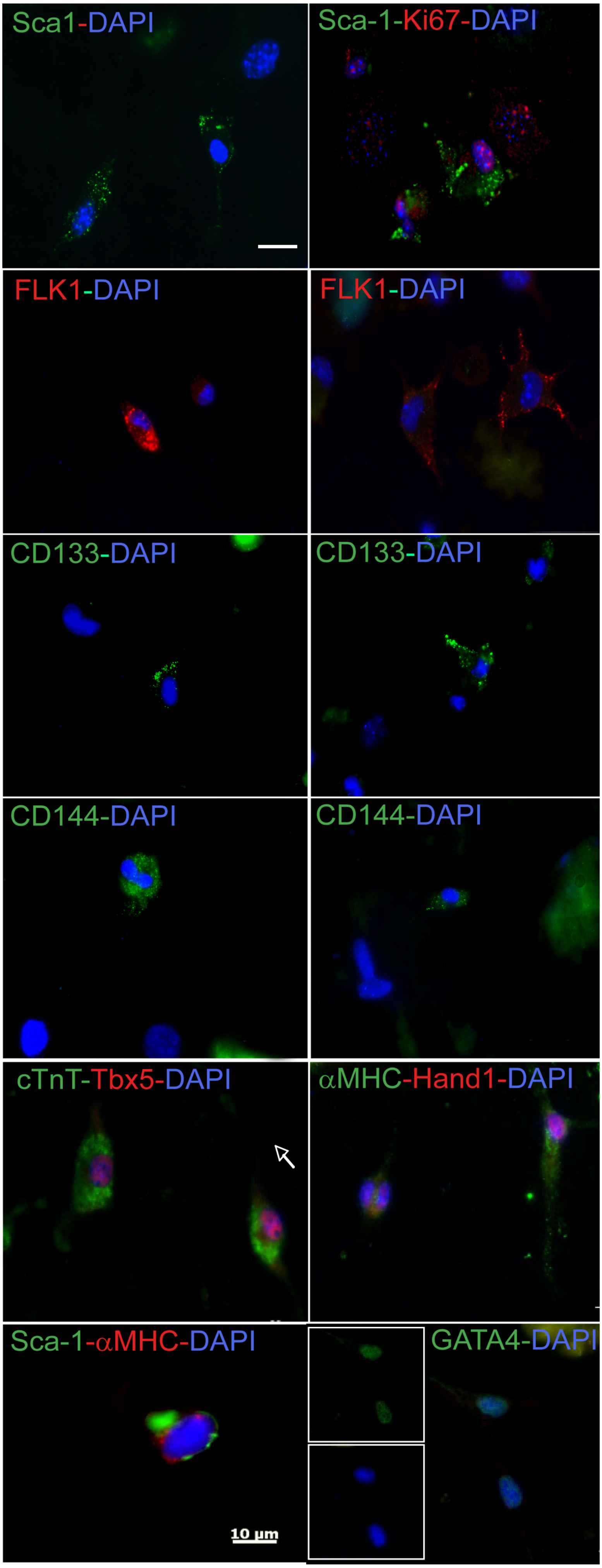

Representative immunofluorescence pictures of CPC co-cultures after 10 days of cultivation confirming the phenotype observed by light microscopy and FACS analysis. Positive stainings for endothelial marker Flk1, CD133, and CD144; and cardiac marker αMHC, Hand1, Tbx5, cTnT and GATA4 are shown. *Scale bar: 20 µm.*

Figure VII

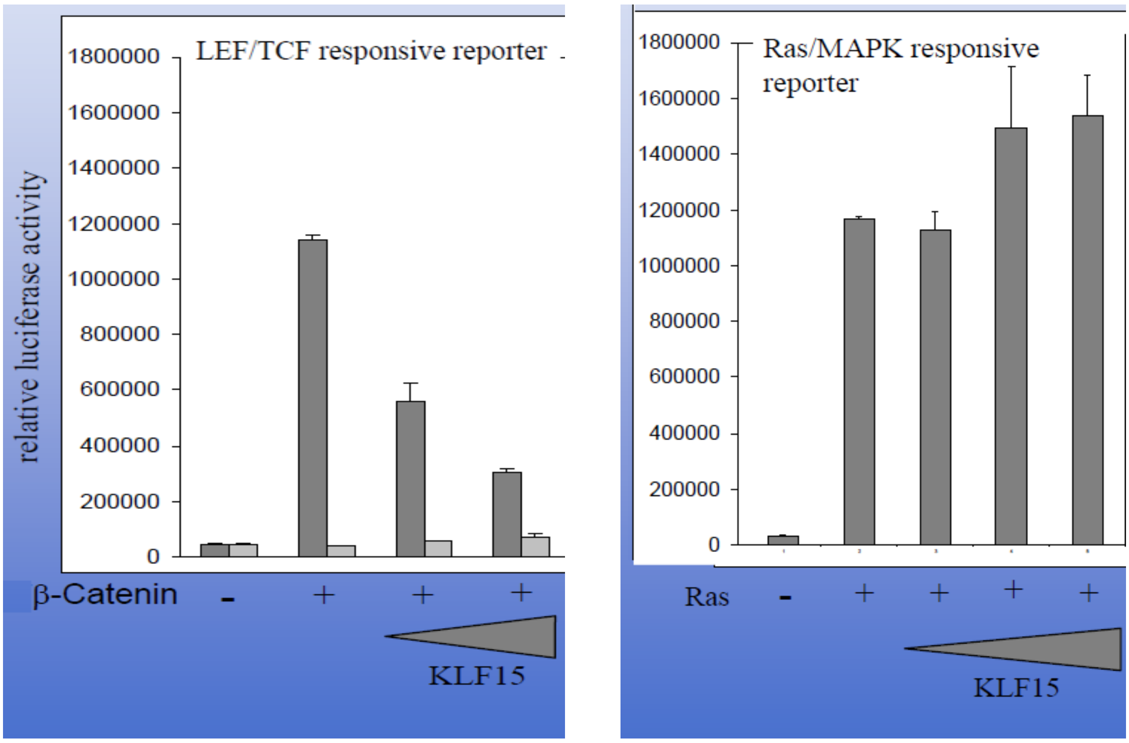

KLF15 blocks LEF/TCF mediated transcription without affecting unrelated reporter systems in HEK293 cells. KLF15 significantly inhibited β-catenin-dependent reporter activation in a concentration-dependent manner. Ras/MAPK luciferase reporter activation is not affected upon KLF15 expression. *pFOPflash served as negative control (gray bars) and Renilla luciferase for normalization.*

Figure VIII

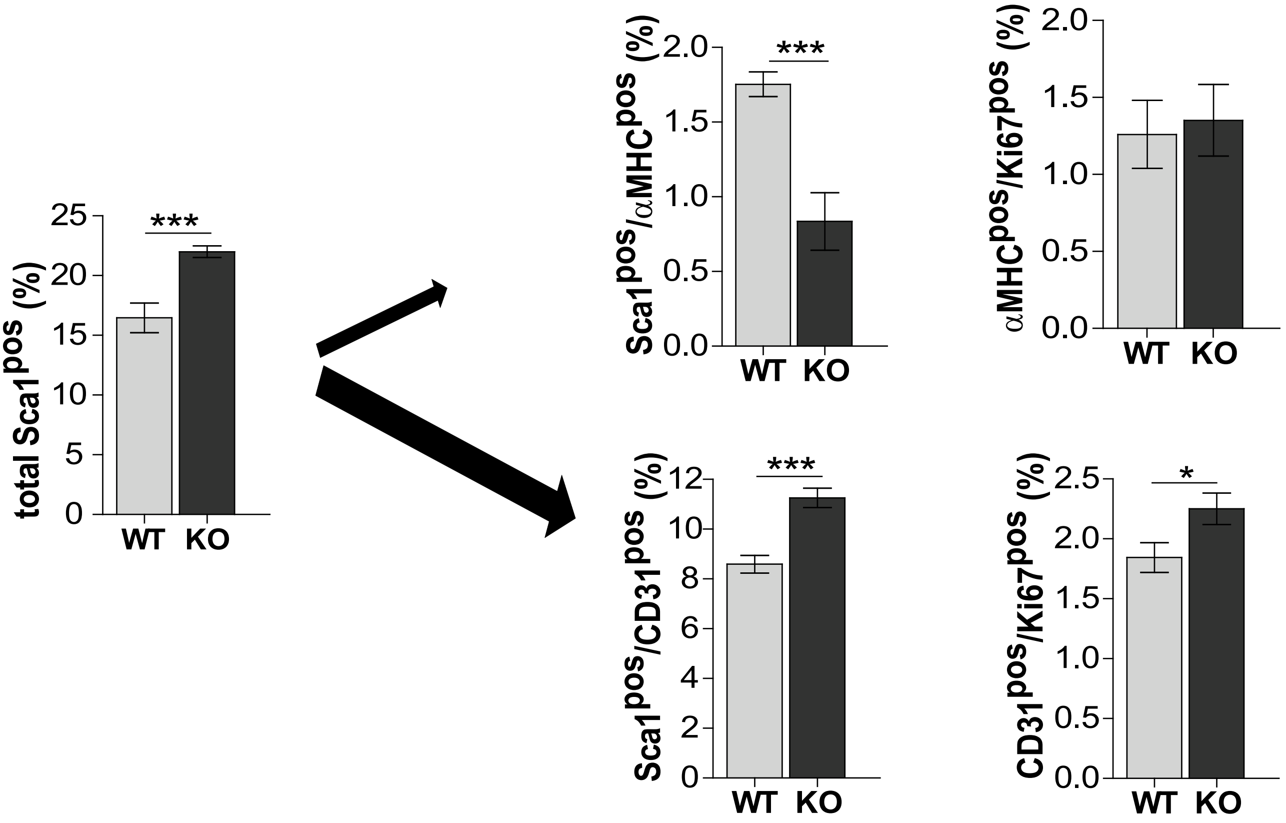

An increased Sca1 cell pool results upon KLF15 depletion, which differentiates towards an endothelial fate while decreasing the cardiogenic cell population. The absence of KLF15 displaces the balance in favour to an endothelial lineage that continuously increases. In other words our data suggest that a more uncommitted Sca1 cell pool proliferate and differentiate towards different cell lineages according to the activating signalling.

2nd Editorial Decision 04 January 2012

Thank you for your letter asking us to re-consider our decision in light of your point-by-point response to reviewers that I have now read with attention. In the meantime, we also have received the last referee's report (copied below). In light of your letter, and this last report, we would like to invite you to submit a major revision of your manuscript.

You will see that while the last referee also find your study of interest, some specific points are detailed that need to be addressed.
Concerning your point-by-point letter, I would like to strongly encourage you to add endogenous data asked for by both referees #1 and #2 otherwise, based on over-expression experiments, the study does not provide the physiological relevance important for our Journal. Also regarding Referee #1's issues, while I do not think that a conditional KO strategy is needed here, I would tend to agree that lineage tracing would considerably strengthen your manuscript by showing that KLF15 drives CPCs into endothelial lineage, which is one of the main message of the manuscript.

Specific points:
Ref.1 point 2: please provide biological triplicates
Ref.1 point 4 (last sentence): please add the c-Kit population data to the main manuscript
Ref.1 point 5 (last sentence): please provide co-immunostainings and better quantification
Ref.1 point 6: please provide a CD31 immunostaining on heart sections from control and β-cat ex2-6 mice
Ref.3 point 2: please provide a siRNA experiment
Ref.3 points 3 and 4: the mutation analysis in NRCs will not be necessary however a clearer and more detailed explanation on the cells chosen and at which step is required
Ref.3 point 5: please provide another immunofluorescence picture
Ref.2 point 1: please show KLF15 protein level in Sca1+ population

All other points should be at least discussed in detail in the point-by-point letter.

Please note that it is EMBO Molecular Medicine policy to allow only a single round of revision and that, as acceptance or rejection of the manuscript will depend on another round of review, your responses should be as complete as possible.

Revised manuscripts should be submitted within three months of a request for revision; they will otherwise be treated as new submissions, except under exceptional circumstances in which a short extension is obtained from the editor. Also, the length of the revised manuscript may not exceed 60,000 characters (including spaces) and, including figures, the paper must ultimately fit onto optimally ten pages of the journal. You may consider including any peripheral data (but not methods in their entirety) in the form of Supplementary information.

I look forward to seeing a revised form of your manuscript as soon as possible.

Yours sincerely,

Editor
EMBO Molecular Medicine

***** Reviewer's comments *****

Referee #2:

In their very interesting manuscript Noack et al. describe the identification and characterization of a novel cardiac interaction between Krueppel-like factor 15, beta-catenin, and TCF4. They found that this inhibits β-catenin-dependent transcription. They show their findings based on in vitro mutation analyses, reporter assays and co-localization of these factors. Thereby they define domains within the KLF15 molecule responsible for its nuclear localization (C-terminus) and for its transcriptional

repression (small N-terminal region). Next, they used Klf15 knock-out mice, which revealed an increased cardiac β-catenin transcriptional activation accompanied with cardiac deterioration during aging or elicited by stress. They show that these phenomena are caused by increased endothelial progenitors at the expense of cardiogenic progenitors in the Klf15 knock-out mice. Based on this they propose that the novel interaction between KLF15 and Wnt/β-catenin components is crucial for normal cardiac tissue homeostasis, which the authors find comparable to embryonic cardiogenesis with respect to cell fate. They propose that their findings might become a basis to activate endogenous cardiac progenitor cells in adult damaged hearts. While this hope is still somewhat speculative the experiments underlying manuscript were in general well and comprehensively performed.

I have the following points to be mentioned:

1) In Fig. 5A the authors show KLF15 expression in the Sca1+ cell-population merely via qRT-PCR. This should be done on the protein level as KLF15 expression in the purified cells is a crucial prerequisite for the following experiments.

2) In Fig. 7B the authors show data from Flk1-FACS. Why do they describe Flk-1 as the "earliest endothelial progenitor marker"? To my knowledge Flk-1 as a very early lateral plate mesoderm marker is also expressed in cardiopoietic cells. Why not use CD31 instead?

3) The authors finding that "Interestingly, the reduction of the cardiogenic Tbx5pos/cTnTneg population was more accentuated in TAC-operated Klf15 KO mice, compared to TAC-operated WT mice" (Fig. 6B) seems somewhat contradictory to their conclusion of increased endothelial progenitors at the expense of cardiogenic progenitors in the Klf15 knock-out mice (see above). This needs at lest to be discussed in detail.

4) The gel figures should be better described in the main text as well as the figure legends. These figures are quite complex and it is sometimes hard for the reader to follow the information of the different lanes. E.g. the authors could write in the main text: "Fig. X, lane Y shows that..." etc.

5) In a number of places statements are given without citations. E.g.:

"TCF4 and NLK co-expression was used as a positive control since NLK was shown to target TCF4 for ubiquitination."

"...CPCs were treated with 5 mol/L Quercetin, a known β-catenin transcriptional inhibitor."

1st Revision - Authors' Response 04 April 2012

With this point-by-point discussion we would like to address the questions/concerns emerged by our manuscripts.

We would like to thank all referees for their comments, which helped us experimentally and conceptually to further strengthen our study.

We really appreciate their evaluation.

Referee#1

"In this manuscript, Noack et al. demonstrated that Krueppel-like factor 15 (Klf 15) interacts with β-catenin, T-cell factor 4 and NEMO-like kinase, thereby inhibiting β-catenin dependent transcriptional activation. They also showed that Klf15 negatively regulates β-catenin downstream targets. They observed an increase in endothelial cell markers in the Sca1+ sorted cardiac progenitor cells from Klf15 KO mice and claimed that Klf15 controls differentiation of cardiac progenitor cells (CPCs) towards endothelial cells.

The conclusion regarding the function of Klf15 in cardiac progenitor cell fate is not well justified. Although the observation of CPC fate switch is intriguing, this conclusion is weakened by the lack of in vivo cell fate analysis and lineage tracing studies. The interactions of Klf15 with components of the Wnt/beta-catenin pathway were mainly carried out by protein overexpression in vitro, and the authors should present data for endogenous protein interactions to strengthen their conclusions".

"The present study contradicts published data that Klf15 KO mice show eccentric cardiac hypertrophy, and that Klf15 can inhibit GATA4 and myocyte enhancer factor 2 (MEF2) function. It is not clear how to reconcile this discrepancy".

It should be noted that our study does not really contradict previous findings. The study of Fisch et al (Fisch et al, 2007) showed evidences of KLF15 as a negative regulator of cardiac hypertrophy in part through inhibition of MEF2 and GATA4 transcriptional pathways. These conclusions were made based on the analysis of KLF15 knockout (-/-) mice following induced hypertrophy via aortic constriction. Nevertheless, the study did not cover the analysis of cardiac progenitor cells at baseline upon KLF15 loss-of-function in vivo, which is the main and the novel observation in our study.

Additionally, our present study covers the analysis of cardiac progenitor cells in KLF15 KO mice under chronic AngII-induced hypertrophy and hemodynamic stress induced by transverse aortic constriction (TAC). Similar to the observations made by Fisch et al. we observed reduced fractional shortening at baseline in 12- to 16-week-old KLF15 functional knockout (KO) mice, although we did not observe increased left ventricular cavity size, which may be explained by mouse strain differences. The study of Fisch et al reported no significant difference between KLF15 (+/+) and (-/-) myocyte area at baseline, which fits with our echocardiographic data indicating no difference in wall thickness between KLF15 KO and control mice. Moreover, we investigated apoptosis via TUNEL assay in 12- to 16-week-old KLF15 KO mice (Figure S3 and Table S2) and no difference was detected when compared with the control mice, which is in line with the TUNEL staining, immunohistochemistry for cleaved caspase-3 and apoptotic gene expression analysis performed by Fisch et al. However, we do not find evidence of cardiac hypertrophy and/or fibrosis to be the main mechanism as indicated by myocyte area, echocardiographic showing no difference in wall thickness and measurement of hypertrophic and fibrosis pathway activation in KLF15 KO in comparison to control at baseline.

In summary, our study complements previous findings concerning the role of KLF15 in normal cardiac homeostasis at baseline and in the stressed heart. Altered CPC homeostasis might be another cellular mechanism aside cardiac hypertrophy that contributes to enhanced cardiac deterioration upon deletion of KLF15. As mentioned in the discussion both scenarios are not mutually exclusive, and may contribute to heart failure development.

"Given Klf15 KO may affect many cell types during heart development, a conditional KO strategy should be carried out to determine the cell type specific function of Klf15 in adult CPCs. These major issues need to be addressed to support their conclusion."

Expression of KLF15 was previously documented after postnatal day 30 (Gray et al, 2007; Zhou et al, 2011). Therefore, the influence of KLF15 during heart development may not be as relevant as it is for the adult cardiovascular system.

Aiming to elucidate the cell type specific function of KLF15 we isolated the CPCs and analyzed them in vivo and in vitro (as shown in Fig. 5 and 7 in Manuscript). We also would like to emphasize that independent from the effect of KLF15 in cardiomyocytes and fibroblasts, this analysis clearly showed that KLF15 deletion controls CPC cell fate in a cell autonomous manner since acquisition of endothelial fate upon KLF15 deletion in vivo was also observed in vitro in isolated CPCs.

Specific comments:
1. The authors show that Klf15 interacts with β-catenin by overexpressing two genes in cell culture. This interaction should be confirmed endogenously in NRC without overexpressing the genes.

We fully agree with this point and now provide evidences of KLF15 interaction with b-catenin, NLK and TCF4 in vivo employing adult heart tissue. We would like to mention that the limitation for this experiment is the quality of the current available antibodies for KLF15 and NLK (added to new Figure 1A-C, lane 8 of the manuscript).

2. Figure 2D: By overexpressing Klf15 and Nlk, ubiquitination of TCF4 is increased. Ubiquitination of endogenous TCF4 by Klf15 should be performed in NRC.

We provide evidence of decreased endogenous TCF4 ubiquitination in adult mouse heart cells of KLF15 KO mice (new Supplemental Figure S3 of the manuscript). This assay showed a slight but significant reduction in TCF4 ubiquitination in KLF15 KO in comparison to control mice (Supplemental Fig. S3A, $P<0.05$), in agreement to our in vitro data showing a significant increase in TCF4 ubiquitination upon KLF15 overexpression.

3. In Figure 4C, cardiac function was measured by ejection fraction. The difference between WT and KLF15 is quite subtle, fraction shortening should also be measured.

We provide these data (new Figure 4C and D of the manuscript).

4. Figure 5 shows that KLF15 controls cardiac progenitor cell fate in isolated Sca1 positive cells. Lineage tracing should be performed to examine whether Klf 15 expressing cells lead to an endothelial lineage.

It should be noted that we provide evidences of KLF15 loss-of-function favoring the expansion and commitment of CPCs to endothelial cells. We analyzed the effect of KLF15 depletion in Sca1 and c-kit cells (new Fig. 5D). We further extended our analysis on Sca1 cells since they showed expression of KLF15 and so far they are described as the putative CPCs with the greatest potential for cardiomyogenic differentiation (Matsuura et al, 2004; Oh et al, 2003b; Pfister et al, 2005; Tateishi et al, 2007). Here, we would like to emphasize that we showed clearly that CPCs containing Sca1 cells acquire a preferentially endothelial phenotype upon β-catenin/TCF upregulation in the absence of KLF15. In contrast, WT CPCs (which indicates a normal expression of KLF15) showed a different balance of CPC. These small cells in transition cells are also able to express CD31 as demonstrated by FACS analysis of the Sca1/CD31 positive population and by the expression of CD31 in WT differentiated CPCs in co-culture. Our data does not exclude that KLF15 expressing CPCs may become in endothelial cells as well. Thus, Sca1 cells, expressing KLF15, are able to differentiate towards endothelial cells as demonstrated by FACS and immunofluorescence analysis as well as qRT-PCR (new Fig. 5 and 7).

We provide now an additional sub-figure documenting the expression of CD31 in the tube-like formation of KLF15 KO and WT differentiated CPCs in co-culture. Co-staining of Sca1 in these structures is rather unusual to find, since very few cells "in transition" are found in those structures. Undifferentiated cells normally undergo stepwise differentiation, which would lead to the detection of small committed cell in transition but eventually the mature cells lose the expression of undifferentiated markers as Sca1 as described for other markers (Qyang et al, 2007). KLF15 maintains a balance of early differentiating CPCs and this deregulation results in an imbalance of the cardiomyogenic and endothelial differentiating CPCs in comparison to the normal homeostasis, which does not exclude the ability of KLF15 expressing Sca1 cells to give rise to endothelial cells.

Although, we were aware of the difficulties of an in vitro tracing assay employing adult CPCs, we planned an in vitro lineage tracing doable in the available time. We employed a lentiviral vector encoding GFP to genetically manipulate CPCs. The LentiORF vector (Open Biosystem) was used to label overexpression of KLF15 in CPCs. This vector expresses the KLF15 ORF along with the fluorescent marker (GFP) and the selection marker from one promoter, providing a visual marker of KLF15 overexpressing transfected CPCs. The ability of the vectors to overexpress KLF15 was tested in L-Wnt-3A cells (Fig. IIA). CPCs were isolated, purified by MACS for Sca1+/Lin- lineage and subsequently transduced with either the KLF15 lentiviral vector or a GFP control

vector. After selection and proliferation, the cells were sorted for GFP and co-cultured on adult cardiac fibroblasts for 10 days (Fig. IIB). Unfortunately, many cells looked senescent and unhealthy especially upon KLF15 overexpression most of the cells were morphologically abnormal and died. Therefore, a reliable analysis of the KLF15 overexpressing cells was not possible. Only very few GFP positive small cells were detected to express endothelial markers (Fig. IIC) but these cell did not survive longer in culture. We assume the overexpression of KLF15 (as shown by the qRT-PCR and Western blot analysis), exceeded by far the physiological expression and thus resulting in the impairment of CPC differentiation.

We agree that a lineage tracing analysis would further support our study. An ideal tracing would be performed in mice carrying a reporter protein fused to KLF15 expression but we honestly believe that this analysis is out of the scope of the present manuscript.

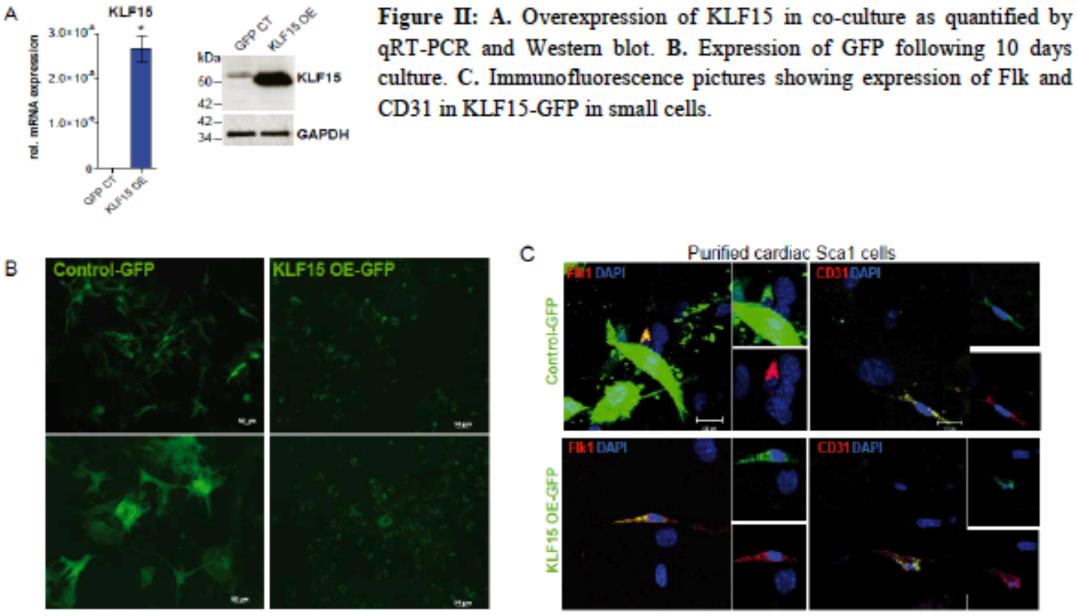

Figure II: A. Overexpression of KLF15 in co-culture as quantified by qRT-PCR and Western blot. **B.** Expression of GFP following 10 days culture. **C.** Immunofluorescence pictures showing expression of Flk and CD31 in KLF15-GFP in small cells.

Since part of Klf15 coding region is replaced with the lacZ gene, β-gal positive cells should be examined in adult Klf5 KO heart.

Unfortunately, the inserted LacZ cassette is not functional, since it was only partly inserted to replace exon 1 and 2 of Klf15.

Also, use of Sca 1 as the only CPC marker in this study is not sufficient, and other cardiac progenitor markers such as c-kit and Islet 1 should be tested with the CPC cells as well.

Specific groups of CPC populations have been identified and characterized including c-Kit (Beltrami et al, 2003), Islet-1 (Wang et al, 2006), Sca1 (Oh et al, 2003a), side population cells (Pfister et al, 2010) and cardiac neural crest-derived cells (Tomita et al, 2005). However, evidences concerning the exact nature, propensity to differentiate into cardiovascular cells and identification of these cells in the adult myocardium are currently diverse.

The authors agree that cardiac Sca1 cells are not fully representative of CPCs in the adult heart. However, the percentage of these cells is more prominent in the adult heart in comparison to other populations previously described. We included now in our study the analysis of the c-kit population (new Fig. 5D). In contrast to the total 16,46±1,23 % Sca1 cells that we observed in WT 12-weeks old mice, we found 0.88±0.10% c-Kit cells in the cardiomyocyte-depleted fraction, which is similar to previous finding by Zaruba et al. in which less than 1% total c-kit cells in both neonatal and adult heart was reported (Zaruba et al, 2010) (this is now discussed in the revised manuscript).

Moreover, we were not able to observe a clear Islet1 positive population by analyzing the cardiomyocyte-depleted fraction by FACS and immunofluorescence. Our finding are in agreement with recently observations showing the absence of Islet1 cells from the working myocardium (Weinberger et al, 2012), and the dispute role of this cell population as a second heart filed progenitor of the adult heart (Engleka et al, 2012).

We would like to mention that the Sca1 population is very well documented in several studies and described as the CPCs with the greatest potential for cardiomyogenic differentiation. They were shown to have the potential as stem cells to differentiate in vitro and may contribute to the regeneration of injured adult murine hearts (Matsuura et al, 2004; Oh et al, 2003b; Pfister et al, 2005; Tateishi et al, 2007), which make these cells suitable to test the regulation of signalling pathways, which may affect CPCs of the adult heart.

5. Figure 7: If the Sca1 positive CPC cells are proliferating, why do the authors perform the co-culture with fibroblast cells?

The co-cultures are performed with adult cardiac fibroblasts to mimic the endogenous cardiac environment. Fibroblasts are important cells supporting cardiac differentiation and homeostasis, which is independent from the proliferative capacity of the co-cultured cells. The use of conditional medium may add/remove components that are not known, making it difficult to control the quality and reproducibility of the medium (Li et al, 2005). It has been found that cells are maintained in tissue culture much more readily if they are supported on substrate components most closely resembling the extracellular matrix (ECM) in which they occur in vivo (Baharvand et al, 2005). The matrix provided by fibroblasts was shown to be one of the best. Currently, several studies are switching from matrigel to extracellular ECMs from decellularized fibroblasts, which is proposed to be essential for proliferation, growth and differentiation, which may be an alternative to fibroblast feeder layer.

We also performed some analysis using matrigel instead of fibroblasts as feeder layer but unfortunately cell differentiation was not optimal in that case. This is in line with the fact that matrigel inhibits the differentiation of cells due to all the different protein content and alteration of protein expression. The matrigel composition and structure was shown to inhibit TGF-b2 gene expression (LaGier et al, 2007).

Immunostaining of cardiac and endothelial markers should be performed on the Sca1 positive CPC cells from the WT and KLF15 KO mice in 7C.

We provide these data (new Figure 7C of the manuscript). Please be aware that no visual differences are easily observed. The representative microscopical pictures of the CPCs co-cultured for 15 days in 7C are photographed using a 10x objective while the immunofluorescence picture are normally taken with 40x.

Quantification of cell numbers should be *performed in 7D.*

We would like to emphasize that the quantification of the representative picture in Fig. 7D is depicted for different cell types and total proliferative cells in Fig. 7A using FACS analysis. Now, we provide an additional semi-quantification of Fig. 7D (added to new Figure 7D of the manuscript).

6. Increased endothelial lineage of CPC in Klf15 KO mice needs to be confirmed in vivo by immunostaining of CD31 on heart sections from control and β-cateninΔex2-6 mice.

We analyzed KLF15 KO as well as b-cat$^{Dex2\text{-}6}$ mice via immunoperoxidase staining due to the higher sensitivity of this approach. Paraffin-embedded sections of heart samples were positively and specifically stained by CD31 using the peroxidase anti-peroxidase (PAP) soluble complex (Sigma) for amplification of the signal. A semi-quantification analysis showed a significant relative upregulation of CD31 positive area in KLF15 KO mice in comparison to WT controls in contrast to a downregulation observed in b-cat$^{Dex2\text{-}6}$ animals ($n\geq3$, new Figure 6C and D of the manuscript). These observations further support our previous observations by FACS analysis in vivo.

7. Figure 2A: KLF15 represses luciferase activity on the control vector, does it mean there is a nonspecific sequence on the vector independent of the TCF binding site?

Although the FOPflash control vector contains 3 mutated Tcf/Lef binding elements upstream to the luciferase promoter, a minimal background expression is always detectable. Co-expression of the positive regulators β-catenin and TCF4 resulted not only in activation of the TOPflash system, but also in weak activity of its control vector in SW480, HEK293 and NRC. We used SW480 carcinoma cells since they are characterized by a constitutive active Wnt/β-catenin pathway. Co-expression of activating TCF4 with FOPflash resulted in a higher activation of the control system in SW480 compared to HEK293 cells or NRC, showing that the control vector slightly reacts to present β-catenin/TCF. Accordingly, co-expression of KLF15 results in a weak inhibition of the control vector. Importantly, both observations were statistically not significant.

Moreover, to control for non-specific interactions we tested a Ras/MAPK-responsive luciferase reporter system. Co-transfection with the same KLF15 expressing plasmid showed no regulation (Fig. III), indicating a specific inhibition of β-catenin/TCF-dependent transcription.

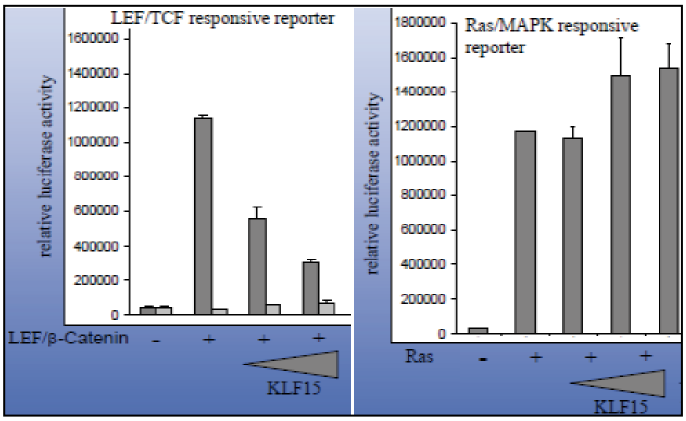

Figure III: KLF15 specifically inhibited β-catenin/TCF-dependent reporter activation in a concentration-dependent manner as demonstrated by LEF/TCF luciferase reporter activation in HEK293 cells. In contrast, KLF15 has no effect on Ras/MAPK activation as demonstrated by a luciferase reporter activation. *pFOPflash served as negative control (gray bars) and Renilla luciferase for normalization.*

8. Figure 5: Sca1 negative cells should be included as a negative control for the RT-PCR.

MACS technique was employed to isolate the Sca1 population from adult heart. We isolated a cardiomyocyte-depleted cell fraction containing CPCs. From this population we were able to detect 16,46±1,23 % Sca1 cells, which were enriched to 96,4 % purity after 2-3 rounds of purification. The collected flow through is not completely depleted from Sca1 cells, since some positive cells remain in this fraction, in our hands the percentage was around 47,6% after the 2-3 rounds of magnetic separation (attached Fig. IV). For that reason we decided to compare our population with the total heart lysate and -RT.

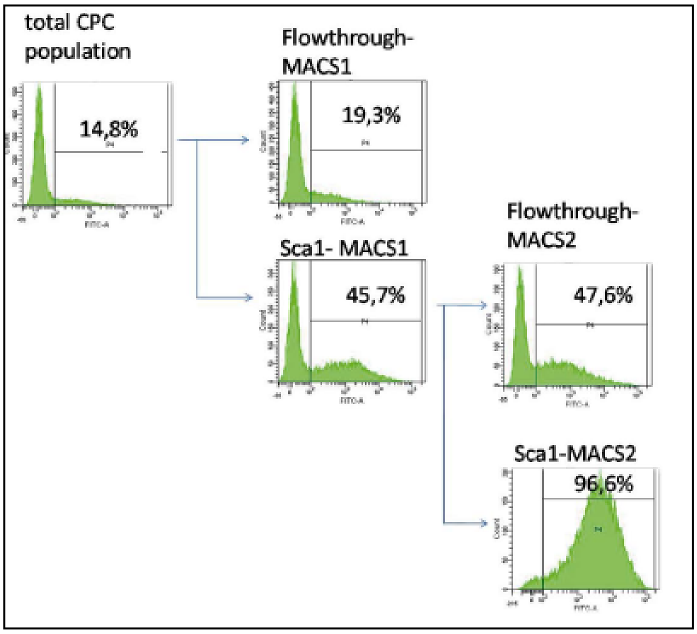

Figure IV: Sca1 MACS purification

8. If there is a decrease in the Sca1pos/αMHCpos cells in 5C, why there is no change in α MHCpos/Ki67pos cells in 5D?

Collectively our data show that KLF15 deletion leads to a decreased amount of Sca1/αMHC cells, which represent a specified proliferative cardiogenic committed population, rather than affecting the more differentiated αMHC proliferating population. Our observation allow us to interpret that a KLF15-dependent Wnt/b-catenin transcriptional regulation affects a more undifferentiated cell population. Deletion of KLF15 results activation of Wnt/b-catenin signaling and directs undifferentiated CPCs towards an endothelial cell fate, which subsequently expands upon Wnt activation. Several studies showing that Wnt/b-catenin signaling is required for proliferation and expansion of specified cardiovascular progenitors during development (Gessert & Kuhl, 2010). We propose a shift in cell fate determination of an undifferentiated population.

9. Figure C, the summary of different KLF15 mutant binding to TCF needs to be aligned

We apologize and corrected this mistake.

Bibliography:

Baharvand H, Azarnia M, Parivar K, Ashtiani SK (2005) The effect of extracellular matrix on embryonic stem cell-derived cardiomyocytes. J Mol Cell Cardiol 38: 495-503

Beltrami AP, Barlucchi L, Torella D, Baker M, Limana F, Chimenti S, Kasahara H, Rota M, Musso E, Urbanek K, Leri A, Kajstura J, Nadal-Ginard B, Anversa P (2003) Adult cardiac stem cells are multipotent and support myocardial regeneration. Cell 114: 763-776

Engleka KA, Manderfield LJ, Brust RD, Li L, Cohen A, Dymecki SM, Epstein JA (2012) Islet1 derivatives in the heart are of both neural crest and second heart field origin. Circ Res 110: 922-926

Fisch S, Gray S, Heymans S, Haldar SM, Wang B, Pfister O, Cui L, Kumar A, Lin Z, Sen-Banerjee S, Das H, Petersen CA, Mende U, Burleigh BA, Zhu Y, Pinto YM, Liao R, Jain MK (2007) Kruppel-like factor 15 is a regulator of cardiomyocyte hypertrophy. Proc Natl Acad Sci U S A 104: 7074-7079

Gessert S, Kuhl M (2010) The multiple phases and faces of wnt signaling during cardiac differentiation and development. Circ Res 107: 186-199

Gray S, Wang B, Orihuela Y, Hong EG, Fisch S, Haldar S, Cline GW, Kim JK, Peroni OD, Kahn BB, Jain MK (2007) Regulation of gluconeogenesis by Kruppel-like factor 15. Cell Metab 5: 305-312

LaGier AJ, Yoo SH, Alfonso EC, Meiners S, Fini ME (2007) Inhibition of human corneal epithelial production of fibrotic mediator TGF-beta2 by basement membrane-like extracellular matrix. Invest Ophthalmol Vis Sci 48: 1061-1071

Li Y, Powell S, Brunette E, Lebkowski J, Mandalam R (2005) Expansion of human embryonic stem cells in defined serum-free medium devoid of animal-derived products. Biotechnol Bioeng 91: 688-698

Matsuura K, Nagai T, Nishigaki N, Oyama T, Nishi J, Wada H, Sano M, Toko H, Akazawa H, Sato T, Nakaya H, Kasanuki H, Komuro I (2004) Adult cardiac Sca-1-positive cells differentiate into beating cardiomyocytes. J Biol Chem 279: 11384-11391

Oh H, Bradfute SB, Gallardo TD, Nakamura T, Gaussin V, Mishina Y, Pocius J, Michael LH, Behringer RR, Garry DJ, Entman ML, Schneider MD (2003a) Cardiac progenitor cells from adult myocardium: homing, differentiation, and fusion after infarction. Proc Natl Acad Sci U S A 100: 12313-12318

Oh H, Bradfute SB, Gallardo TD, Nakamura T, Gaussin V, Mishina Y, Pocius J, Michael LH, Behringer RR, Garry DJ, Entman ML, Schneider MD (2003b) Cardiac progenitor cells from adult myocardium: Homing, differentiation, and fusion after infarction. Proc Natl Acad Sci U S A 100: 5834-5839

Pfister O, Mouquet F, Jain M, Summer R, Helmes M, Fine A, Colucci WS, Liao R (2005) CD31- but Not CD31+ Cardiac Side Population Cells Exhibit Functional Cardiomyogenic Differentiation. Circ Res: 01.RES.0000173297.0000153793.fa

Pfister O, Oikonomopoulos A, Sereti KI, Liao R (2010) Isolation of resident cardiac progenitor cells by Hoechst 33342 staining. Methods Mol Biol 660: 53-63

Qyang Y, Martin-Puig S, Chiravuri M, Chen S, Xu H, Bu L, Jiang X, Lin L, Granger A, Moretti A, Caron L, Wu X, Clarke J, Taketo MM, Laugwitz KL, Moon RT, Gruber P, Evans SM, Ding S, Chien KR (2007) The renewal and differentiation of Isl1+ cardiovascular progenitors are controlled by a Wnt/beta-catenin pathway. Cell Stem Cell 1: 165-179

Tateishi K, Ashihara E, Takehara N, Nomura T, Honsho S, Nakagami T, Morikawa S, Takahashi T, Ueyama T, Matsubara H, Oh H (2007) Clonally amplified cardiac stem cells are regulated by Sca-1 signaling for efficient cardiovascular regeneration. J Cell Sci 120: 1791-1800

Tomita Y, Matsumura K, Wakamatsu Y, Matsuzaki Y, Shibuya I, Kawaguchi H, Ieda M, Kanakubo S, Shimazaki T, Ogawa S, Osumi N, Okano H, Fukuda K (2005) Cardiac neural crest cells contribute to the dormant multipotent stem cell in the mammalian heart. J Cell Biol 170: 1135-1146

Wang X, Hu Q, Nakamura Y, Lee J, Zhang G, From AH, Zhang J (2006) The role of the sca-1+/CD31- cardiac progenitor cell population in postinfarction left ventricular remodeling. Stem Cells 24: 1779-1788

Weinberger F, Mehrkens D, Friedrich FW, Stubbendorff M, Hua X, Muller JC, Schrepfer S, Evans S, Carrier L, Eschenhagen T (2012) Localization of Islet-1-Positive Cells in the Healthy and Infarcted Adult Murine Heart. Circ Res

Zaruba MM, Soonpaa M, Reuter S, Field LJ (2010) Cardiomyogenic potential of C-kit(+)-expressing cells derived from neonatal and adult mouse hearts. Circulation 121: 1992-2000

Zhou J, Tan T, Tian Y, Zheng B, Ou JH, Huang EJ, Yen TS (2011) KLF15 activates hepatitis B virus gene expression and replication. Hepatology

Referee #2

In their very interesting manuscript Noack et al. describe the identification and characterization of a novel cardiac interaction between Krueppel-like factor 15, beta-catenin, and TCF4. They found that this inhibits β-catenin-dependent transcription. They show their findings based on in vitro mutation analyses, reporter assays and co-localization of these factors. Thereby they define domains within the KLF15 molecule responsible for its nuclear localization (C-terminus) and for its transcriptional repression (small N-terminal region). Next, they used Klf15 knock-out mice, which revealed an increased cardiac β-catenin transcriptional activation accompanied with cardiac deterioration during aging or elicited by stress. They show that these phenomena are caused by increased endothelial progenitors at the expense of cardiogenic progenitors in the Klf15 knock-out mice. Based on this they propose that the novel interaction between KLF15 and Wnt/ β-catenin components is crucial for normal cardiac tissue homeostasis, which the authors find comparable to embryonic cardiogenesis with respect to cell fate. They propose that their findings might become a basis to activate endogenous cardiac progenitor cells in adult damaged hearts. While this hope is still somewhat speculative the experiments underlying manuscript were in general well and comprehensively performed.

I have the following points to be mentioned:

1) In Fig. 5A the authors show KLF15 expression in the Sca1+ cell-population merely via qRT-PCR. This should be done on the protein level as KLF15 expression in the purified cells is a crucial prerequisite for the following experiments.

We fully agree with this point and now provide evidences of KLF15 protein expression in the Sca1 purified cell fraction from the adult heart (new Figure 5B of the manuscript).

2) In Fig. 7B the authors show data from Flk1-FACS. Why do they describe Flk-1 as the "earliest endothelial progenitor marker"? To my knowledge Flk-1 as a very early lateral plate mesoderm marker is also expressed in cardiopoietic cells. Why not use CD31 instead?

The point arose by the reviewer is absolutely correct. The receptor tyrosine kinase Flk1 is an important marker of early mesoderm during development. It characterizes the common cardiac progenitor together with the expression of Mesp1. Indeed, specification of the common cardiac progenitor is a multi-step process that involves generation of mesodermal progenitor cell and subsequent intermediate cell populations (Gessert & Kuhl, 2010). Two phases of Flk1 expression have been defined in embryonic stem cell culture experiments (Kattman et al, 2006; Kouskoff et al, 2005). Those cells expressing Flk1 along with the pan-mesodermal marker Brachyury (Bry) but not Mesp1 are fated to become the hemangioblasts that subsequently develop into endothelial cells and the blood lineage. A second wave of Flk1 expression along with Bry and Mesp1 results in a progenitor cell pool for the cardiac lineage (Gessert & Kuhl, 2010). Expression of Flk1 is sequentially lost in the second population while its expression persists in the endothelial lineages. Active Wnt/b-catenin signaling activates these mesodermal progenitors to become hemangioblasts and inactivation of Wnt/b-catenin through Notch will allow them to become cardiogenic cells (Chen et al, 2008; Koyanagi et al, 2007). Interestingly, reduced number of mesodermal Flk1 positive cells impaired hemangioblast development during mouse embryogenesis (Ishitobi et al, 2011) showing the importance of this factor for the endothelial and hematopoietic population.

We observed in the present study that activation of Wnt/b-catenin-dependent transcription upon KLF15 deletion direct Sca1 cell fate towards endothelial like cells. Similarly, it was shown that Notch signaling redirects Flk1 cells fated to become hemangioblasts toward Flk1 cells of the cardiogenic lineage (Chen et al, 2008).

We interpret our data as the Sca1 cells (as a representative cardiac progenitor cell population) upon KLF15 deletion have a similarity to the hemangioblastic Bry+/Flk+/Mesp1- cells, able to become endothelial cells. These cell population show an initial upregulation of Fkl1 followed by CD31 expression, which is accentuated under a KLF15 KO background.

The reason why cells expressing CD31 protein were not measured in the 10-day co-culture experiment is that at the end point of the co-culture the cells start differentiating but they are not mature enough to measure a reasonable percentage of CD31 cells. This observation supports the above mentioned hypothesis, an early upregulation of a more mesodermal hemangioblastic cell, which leads to a final upregulation in the endothelial population (in vivo). We reasoned that mRNA expression analysis will be more sensitive to detect such a change. Therefore, we performed an additional co-culture experiment and provide the mRNA expression data, which confirms the significant upregulation of CD31 expression in the co-culture KLF15 KO in comparison to WT CPCs along with immunofluorescence data showing the expression of CD31 protein in the network formation after 15 days of culture. We also provide additional data confirming the downregulation of Hand1 expression in co-culture KLF15 KO CPCs at the mRNA level (new Figure 7B of the manuscript). We also extended our analysis and show an additional piece of evidence that further supports our observations: a semi-quantification analysis of CD31 expression in heart slides of KLF15 KO mice versus WT and b-cat^{Dex2-6} animals, which depicted a significant relative upregulation of CD31 positive area in KLF15 KO mice in comparison to WT controls (new Figure 6C and D of the manuscript).

3) The authors finding that "Interestingly, the reduction of the cardiogenic Tbx5pos/cTnTneg population was more accentuated in TAC-operated Klf15 KO mice, compared to TAC-operated WT mice" (Fig. 6B) seems somewhat contradictory to their conclusion of increased endothelial progenitors at the expense of cardiogenic progenitors in the Klf15 knock-out mice (see above). This needs at lest to be discussed in detail.

It should be noted that the TAC data are not contradictory but complementary to the regulation of CPC at baseline. Our data propose a misbalance of the cardiac and endothelial cell population under Wnt/b-catenin deregulation. An increase in cardiogenic Tbx5^{pos}/cTnT^{neg} cell loss in TAC-operated Klf15 KO could be explained by a general loss of CPCs upon heart failure in combination with the KLF15-dependent phenotype. This experiment along with the AngII induced cardiac hypertrophy upon KLF15 loss-of-function allow us to conclude that deletion of KLF15 controls the cell fate towards endothelial phenotype at baseline as well as upon cardiac stress. We improved the manuscript text for better interpretation of these data.

4) The gel figures should be better described in the main text as well as the figure legends. These figures are quite complex and it is sometimes hard for the reader to follow the information of the different lanes. E.g. the authors could write in the main text: "Fig. X, lane Y shows that..." etc.

We apologize and rewrote this paragraph for better understanding of the readers.

5) In a number of places statements are given without citations. E.g.:

"TCF4 and NLK co-expression was used as a positive control since NLK was shown to target TCF4 for ubiquitination."

"...CPCs were treated with 5 μmol/L Quercetin, a known β-catenin transcriptional inhibitor."

The citations have been carefully revised and included.

Bibliography:

Chen VC, Stull R, Joo D, Cheng X, Keller G (2008) Notch signaling respecifies the hemangioblast to a cardiac fate. Nat Biotechnol 26: 1169-1178

Gessert S, Kuhl M (2010) The multiple phases and faces of wnt signaling during cardiac differentiation and development. Circ Res 107: 186-199

Ishitobi H, Wakamatsu A, Liu F, Azami T, Hamada M, Matsumoto K, Kataoka H, Kobayashi M, Choi K, Nishikawa S, Takahashi S, Ema M (2011) Molecular basis for Flk1 expression in hemato-cardiovascular progenitors in the mouse. Development 138: 5357-5368

Kattman SJ, Huber TL, Keller GM (2006) Multipotent flk-1+ cardiovascular progenitor cells give rise to the cardiomyocyte, endothelial, and vascular smooth muscle lineages. Dev Cell 11: 723-732

Kouskoff V, Lacaud G, Schwantz S, Fehling HJ, Keller G (2005) Sequential development of hematopoietic and cardiac mesoderm during embryonic stem cell differentiation. Proc Natl Acad Sci U S A 102: 13170-13175

Koyanagi M, Bushoven P, Iwasaki M, Urbich C, Zeiher AM, Dimmeler S (2007) Notch signaling contributes to the expression of cardiac markers in human circulating progenitor cells. Circ Res 101: 1139-1145

Referee #3,

Noack et al. investigated a role for KLF15 in the control of regulatory interactions between beta-catenin, NLK, and TCF4. In addition, the investigators addressed a possible functional role for KLF15 in the heart and in cardiac progenitor cells (CPCs) in response to aging and cardiac stress. While prior data demonstrated a role of KLF15 as a repressor of pathological cardiac hypertrophy and fibrosis, Noack et al. provide novel evidence that KLF15 interacts with and regulates beta-catenin activity and localization through associations with NLK and TCF4. In addition to this novel interaction, the authors demonstrate a role for KLF15 in determining CPC cell outcome, where deletion of KLF15 drives CPCs toward an endothelial cell fate via increased beta-catenin transcriptional activity. In keeping with previous findings, Noack et al. further demonstrate that KLF15 KO animals are more susceptible to angiotensin II (Ang II) and transverse aortic constriction (TAC)-induced cardiac stress.
However, their interpretation that KLF15 plays a role in age-related heart deterioration is weakly supported by a single figure (Fig.4C), and not supported by supplementary data (Fig.S3). Collectively, the authors provide fairly convincing evidence to support their interpretation that KLF15 regulates beta-catenin in order to maintain cardiac homeostasis. Nonetheless, in its current form, the manuscript is unsuitable for publication in EMBO Molecular Medicine.

Specific Points:

1. While the authors provide convincing evidence for KLF15 in the interaction with beta-catenin, NLK, and TCF4 in HEK293 cells and neonatal rat cardiomyocytes (NRCs), these data are generated from ectopic overexpression of the factors. Thus, we do not know if endogenous interactions also occur. Demonstration of endogenous interactions is necessary to support the interpretation that KLF15 mediates cardiac homeostasis through association with beta-catenin.

We fully agree with this point and now provide evidences of KLF15 interaction with b-catenin, NLK and TCF4 in vivo employing adult heart tissue. We would like to mention that the limitation for this experiments were the quality of the current available antibodies for KLF15 and NLK (new Figure 1A-C, lane 8 of the manuscript).

2. A co-culture model is employed to demonstrate a role for KLF15 in controlling CPC cell fate. A bioactive food component, quercetin, was used to inhibit beta-catenin in KLF15 KO cells to examine CPC phenotype. However, quercetin has many activities that have effects on cellular redox state, inflammation and proliferation, and thus it is not possible to conclude that the effects are due to suppression of beta-catenin activity.

We used the Quercetin compound to test the hypothesis that blocking b-catenin dependent transcription will reverse the phenotype concerning CPC cell fate switch observed upon KLF15 deletion in vivo. Quercetin treatment resulted in downregulation of the β-catenin target gene c-Myc, although β-catenin expression was unchanged, corroborating a block of β-catenin-dependent transcription as previously described (Park, Chang et al. 2005). We are aware of the ant-ioxidant, anti-inflammatory, pro-apoptotic effects (among others) of Quercetin and for that reason we included the control group Quercetin and non-treated (DMSO) WT CPCs in our experiment setting. No significant changes were observed between groups concerning the endothelial and cardiogenic cell percentage as well as cell proliferation. In contrast, as described in the manuscript we observed

© EMBO

a partial rescue concerning the percentage of endothelial committed cells and a complete rescue concerning the percentage of cardiogenic and proliferative cells in the KLF15 KO CPCs vs. non-treated KLF15 CPCs, which was comparable to the WT CPCs (Quercetin-treated and non-treated WT cells). These observations indicate that the effect observed in the KLF15 KO CPCs upon Quercetin concerning CPC cell fate and proliferation is due to the attenuation of b-catenin-dependent transcription under the employed concentration and conditions. We cannot exclude additional effects of Quercetin concerning other cell properties but our analysis demonstrate that Quercetin has no influence "per se" on the parameters of interest, which are relevant for our study and aim to complement our in vivo evidences. Several papers showed the effect of Quercetin in vitro and in vivo. Particularly, the effect of Quercetin in vitro depends on the cell line and used concentration. Most of the in vitro studies showing decrease of cell viability or proliferation, pro-apoptotic effects as well as anti-oxidant activity of Quercetin were done employing rather a high concentration of Quercetin (50-200 mM) in contrast to the 5 mM used in our study (Alcocer et al, 2002; Martinez-Florez et al, 2005).

We tried an additional assay employing shRNA constructs against TCF4 to further confirm our pharmacological rescue. Unfortunately, the use of different constructs in co-culture did not result in downregulation of TCF4 or c-Myc expression. We tested the effect of 3 different individual constructs from the mouse GIPZ-Lentiviral shRNAmir library (OpenBiosystem) in L-Wnt-3A cells and co-culture CPCs by lipid-based and virus transfection, respectively. L-Wnt-3A cells secrete biologically active Wnt-3A protein with a constitutive Wnt transcriptional activation; therefore we found the cells suitable for testing TCF transcriptional repression by shRNAi. Cells were transfected, selected and cultured L-Wnt-3A cells for 4 days and CPC co-cultures for 10 days. Transfection efficiency was confirmed by checking GFP expression, which is contained in the GIPZ vector. Protein and mRNA expression analysis showed that none of the constructs exerted inactivation of TCF4 mRNA nor in L-Wnt cells neither in the co-cultures when compared with a non-silencing control plasmid. Since the small shRNA fragments can eventually block translation without degradation of the RNA target, we checked expression of c-Myc, which did not show any regulation (attached Fig. I). Unfortunately, due to time limitations we could no test further shRNA constructs against TCF4.

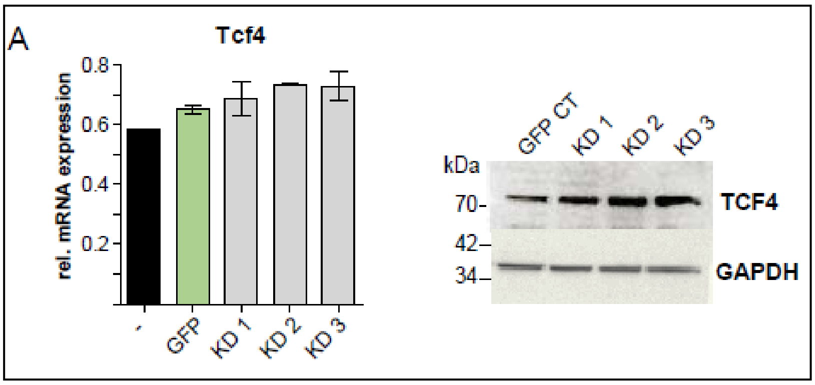

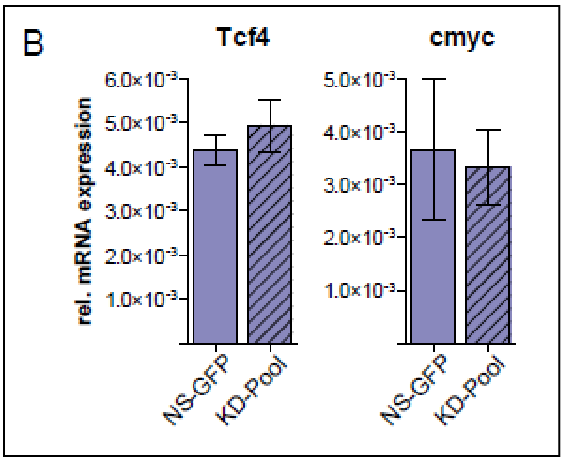

Figure I: A. mRNA and protein expression of TCF4 as quantified by qRT-PCR and Western blot, respectively in control non-silencing and shRNA-TCF4 (KD1-3) transfected L-Wnt-3A cells 4 days pos-transfection. None of the constructs exert TCF4 downregulation. B. TCF4 and c-Myc mRNA expression in co-culture CPCs showing no effect of the tested constructs.

Only one figure (Fig.7E) uses beta-catenin deletion to address CPC phenotype, and this occurs in the presence of KLF15. While in vivo findings support a role for KLF15 in CPC phenotype, further in vitro, mechanistic data would have strengthened the manuscript.

The present study showed KLF15 deletion resulting in b-catenin/TCF transcriptional activation in CPCs in vivo and in vitro exhibiting increased endothelial committed cells along with decreased cardiomyogenic committed cells. The question that arises here is whether direct up- or downregulation of β-catenin transcription, downstream KLF15, would result in similar or opposite results concerning CPC regulation. To test the hypothesis the inducible cardiac β-catenin depleted mouse (b-cat^{Dex3-6}) was used. We previously reported in vitro mechanistic data on cardiac β-catenin depleted and stabilized mice. These data confirm the above mentioned hypothesis, namely CPCs from an inducible cardiac β-catenin depleted mouse (b-cat^{Dex3-6}) showed enhanced cardiomyogenic CPC differentiation in vitro. In contrast, mice with stabilization of b-catenin, (b-cat^{Dex3}) showed a decreased cardiomyogenic CPC differentiation in vitro (Fig. II) (Zelarayan et al, 2008) as found in Klf15 KO mice in the present study. Briefly, Sca1^{pos} cells were isolated, purified by MACS, subsequently labeled with the CM-Dil cell tracer and co-cultured on neonatal cardiomyocytes. After 10 days, co-cultured cells were fixed and stained for a-sarcomeric actinin (a-sr-act), GATA4 and cardiac Troponin T (TropT) for quantification. Sca1^{pos} cells isolated from b-cat^{Dex3-6} mice showed significantly increased differentiation capacity towards a-sr-act, GATA4 and TropT expressing cells in comparison to cells isolated from controls while b-cat^{Dex3} showed an opposite phenotype. This previous finding, provide us the in vitro mechanistic evidence that the role of KLF15 in the adult heart is to regulate b-catenin/TCF transcription and thus controls CPCs homeostasis.

Moreover, in the present study we aimed to complement these finding and showed that cardiac β-catenin depletion not only leads to an increased cardiomyogenic potential of the CPCs but also results in endothelial committed progenitors as indicated by the expression of Sca1 and CD31 by FACS analysis and increased CD31 expression by immunohistochemistry analysis.

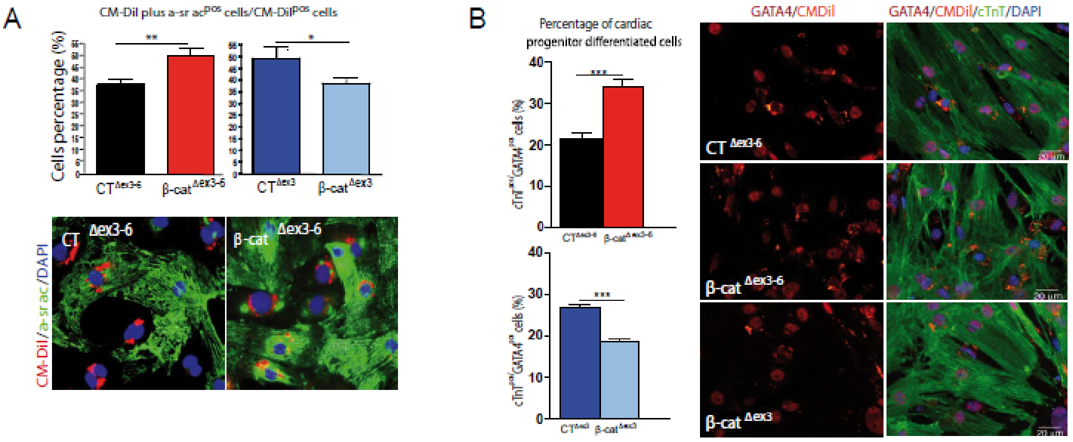

Figure II: Increased *in vitro* differentiation of CMDil labeled-isolated Sca1 cells toward a-Sarcomeric actinin (A) and GATA4^{pos}/cTnT^{pos} cells (B) upon b-catenin depletion in b-cat^{Dex3-6} mice compared to CT cells along with decreased differentiation of the same cell population is observed upon b-catenin stabilization in b-cat^{Dex3} mice compared to CT cells from (Zelarayan et al, 2008).

3. In the Results section the authors mention that NRCs are co-transfected with c-myc-beta-catenin and FLAG-KLF15-full length or N-terminal truncated constructs, and that the data are shown in Fig.1A. While HEK293 appear to show this, the NRCs only show KLF15-beta-catenin. Several discrepancies between the text and the figures were noted throughout the manuscript.

We apologize and corrected these discrepancies and would like to point out that we replaced the co-imunnoprecipitation results obtained in NRC with new data obtained from adult cardiac tissue since all our in vivo analysis were done in adult hearts.

© EMBO

4. The authors mention that KLF15-full length interaction with beta-catenin, NLK, and TCF4 were confirmed in NRCs and that these data could be found in Fig.S1A. However, Fig.S1A contains information regarding HEK293 cells. It is difficult to discern why the authors focus on HEK293 cells in Fig.1 and then jump to NRCs for Fig.2A, to SW480 for Fig.2B, and back to HEK293s for Fig.2C and D. A lack of consistency with cell types makes the results difficult to interpret.

The explanation for the use of different cell types is the transfection efficiency of the different cells used in this study. We decided to show the detailed mutation analysis in a cell line and confirm all the interactions using initially primary NRC and now provided also in adult cardiac tissue. Furthermore, we used NRCs to study the functionality of KLF15 concerning b-catenin/TCF transcription in cardiac cells, but since NRCs have a very low transfection efficiency, HEK293 cells were used to confirmed our observations.. SW480 cells were used to avoid a quadruple transfection, since SW480 cells are a tumor cell line with a constitutively activation of b-catenin/TCF transcription

In summary, HEK293 cells were used to test interactions of different truncated forms of KLF15 with β-catenin, TCF4 and NLK and to confirm the luciferase assay

NRCs were used to confirm these interactions and the KLF15 function in a cardiac cell lineage and SW480 cells were only used for the reporter luciferase assay to show the effect of KLF15 on β-catenin/TCF transcriptional activity.

We improved the text for better understanding of the results concerning the employed cell type.

5. In Fig. 3A, why does the localization pattern of KLF15-N260 and KLF15/beta-cat/DAPI merged with two cells looking distinctly different (i.e. nuclear vs. cytosolic)? In addition, Fig.3C needs correction.

The localization of KLF15-ΔN260 (red) and b-catenin (green) in the new Fig. 2A (former 3A) is nuclear. The immunofluorescence picture shows 2 cells: one of them is bi-nucleated, which shows a more prominent expression of KLF15 and b-catenin in the nucleus and co-localized with the DAPI staining. The nuclear expression in the cell located in the left side of the picture is weaker. This may be due to endogenous expression in contrast to overexpression detected in the bi-nucleated cell. Clear cytosolic expression is only observed in new Fig. 2A bottom (KLF15-DC) as confirmed via Western blot in Fig. 2B. Nonetheless, we provide another immunofluorescence picture.

We apologize and corrected the mistake in Fig. 3C (now new Fig 3E).

6. In Fig.4C, the authors state that cardiac function declined significantly at 12 weeks of age in KLF15 KO animals, yet EF% does not show significance in the figure.

We apologize and corrected the mistake.

Literature:

Alcocer F, Whitley D, Salazar-Gonzalez JF, Jordan WD, Sellers MT, Eckhoff DE, Suzuki K, Macrae C, Bland KI (2002) Quercetin inhibits human vascular smooth muscle cell proliferation and migration. Surgery 131: 198-204

Martinez-Florez S, Gutierrez-Fernandez B, Sanchez-Campos S, Gonzalez-Gallego J, Tunon MJ (2005) Quercetin attenuates nuclear factor-kappaB activation and nitric oxide production in interleukin-1beta-activated rat hepatocytes. J Nutr 135: 1359-1365

Zelarayan LC, Noack C, Sekkali B, Kmecova J, Gehrke C, Renger A, Zafiriou MP, van der Nagel R, Dietz R, de Windt LJ, Balligand JL, Bergmann MW (2008) Beta-Catenin downregulation attenuates ischemic cardiac remodeling through enhanced resident precursor cell differentiation. Proc Natl Acad Sci U S A 105: 19762-19767

3rd Editorial Decision 08 May 2012

Thank you for the submission of your revised manuscript to EMBO Molecular Medicine. We have now received the enclosed reports from the referees that were asked to re-assess it. As you will see the reviewers find the manuscript globally improved. However they still have concerns that should be addressed satisfactorily before to make a decision on your manuscript. As you can see, both referees feel that their criticisms were not fully addressed and while Ref. 1 is still positive, Ref. 2 seems more reserved. I would therefore strongly encourage you to adress these remaining issues as best as you can.

Please submit your revised manuscript within two weeks unless arranged otherwise with the editor. I look forward to seeing a revised form of your manuscript as soon as possible.

I look forward to reading a new revised version of your manuscript as soon as possible.

Yours sincerely,

Editor
EMBO Molecular Medicine

***** Reviewer's comments *****

Referee #1:

The quality of the revised manuscript by Noack et al has been significantly improved. The authors addressed most of the concerns raised by the reviewer. As suggested previously, the conclusion regarding the function of Klf15 in cardiac progenitor cell fate could be strengthened by in vivo lineage tracing studies. However, we nonetheless would accept the paper if the authors satisfactorily address the following points.

Specific comments:

1. Figure 1, the quality of the western blots of endogenous KLF15 in murine heart is sub- optimal. Protein lysate from Klf15 KO heart should be included as a negative control.

2. The size of NLK in Figure 1B lane 8 is different from that in other panels, please explain.

3. Figure S3B, decrease in ubiquitination of endogenous cardiac TCF4 in Klf15 KO is quite subtle. A loading control should be included.

4. Figure 5: Sca1 negative cells or at least cardiomyocyte depleted cells before MACS sorting should be included as a control for the RT-PCR to show enrichment of KLF15 in the purified CPC cells.

5. The expression of vWF and CD105 shown in Figure 5E and 5F seem like the same set of data, please explain.

6. The Quercetin treatment resulted in a decrease in Ki67 positive cells in both WT and KO CPC culture as shown in Figure 7A. Why did Quercetin treatment not affect WT CPC cell number in 7D?

Referee #3:

This is a revised manuscript. The authors have not adequately addressed my original concerns.

1. The co-IP experiments to address endogenous interactions are not convincing (Fig. 1). Control antibodies should be used in all cases, not just in 1A, and multiple independent IPs should be shown.

2. Alternative small molecule inhibitors of Wnt/beta-catenin exist (e.g., ICG-001 and XAV-939 - Selleck Chemicals). I understand the difficulty the authors experienced with RNAi-based approaches for disrupting the pathway. However, it is imperative that they use an agent other than quercitin to confirm the role of beta-catenin in the control of the phenotype in KLF15 KO cells.

2nd Revision - Authors' Response 21 May 2012

Referee #1

Dear Referee #1

We appreciate the time you invested for the evaluation of our revised manuscript. We include all the required controls in the manuscripts and discussed below your questions or concerns.

Specific comments:

1. Figure 1, the quality of the western blots of endogenous KLF15 in murine heart is sub- optimal. Protein lysate from Klf15 KO heart should be included as a negative control.

Unfortunately, employing the Klf15 KO heart tissue lysate as a negative control for the antibody is not possible since the antibody used in this study recognizes an epitope localized at the C-terminus of KLF15. This region is still present in the non-functional Klf15 KO protein (please note that the Klf15 KO sequence contained a partial LacZ cassette, which replaces exon 2 of the coding region, please for details see Supplemental information Figure S2A and B). Briefly, different primers flanking the native exon 2 (rtP1) and 3 (rtP2) of the Klf15 coding region were used to confirm the model. The use of the rtP1 primers binding at exon 2 allows distinguishing between WT and KO KLF15 mRNA since this region is not present in the Klf15 KO sequence. The use of the rtP2 primers binding at exon 3 of the Klf15 coding region showed no differences between the WT and Klf15 KO transcripts. Therefore, the translated non-functional KLF15 KO protein does not differ from the WT protein at the C-terminus.

We would like to mention here that while performing this study we checked different antibodies against either N- or C-terminal epitopes of the KLF15 protein and only the KLF15 antibody from Santa Cruz (sc-34826/C-14) was suitable for western blot analysis. We also tested following antibodies from Abcam: rabbit polyclonal (ab22851), mouse monoclonal (ab81604), goat polyclonal (ab2647) and an additional goat polyclonal not available anymore; from Abnova mouse polyclonal (H00028999-B01). All these antibodies showed either no band or several unspecific bands in Western blot. We tested the suitability of the selected sc-34826 antibody against KLF15 and provide this information in the new Supporting Information Fig. S1B.

2. The size of NLK in Figure 1B lane 8 is different from that in other panels, please explain.

We used different protein marker for the endogenous immunoprecipitation analysis. We adjusted the markers in the figures and text for better understanding.

3. Figure S3B, decrease in ubiquitination of endogenous cardiac TCF4 in Klf15 KO is quite subtle. A loading control should be included.

We believe the small difference in increased endogenous TCF4 ubiquitination in adult mouse heart cells of Klf15 KO mice is underestimated and is due to the very dynamic and rapid process of in vivo ubiquitination. Here, a reliable detection of endogenous protein ubiquitination is hampered by the rapid de-conjugation mediated by de-ubiquitylating enzymes along with a poor detection of an endogenous non-tagged protein. Please notice that this supplemental data are complementing the more reliable in vitro ubiquitination assay.

Technically, we found no way to predetermine the amount of target protein in an immunoprecipitated sample. We determined the amount of total protein present in the initial lysate as measured by Bradford quantitation and used the same amount in each sample (1 mg). We provide a GAPDH control of these lysates.

4. Figure 5: Sca1 negative cells or at least cardiomyocyte depleted cells before MACS sorting should be included as a control for the RT-PCR to show enrichment of KLF15 in the purified CPC cells.

We would like to mention that we did not state that KLF15 is enriched in Sca1^{pos} cells; we just made the observation that KLF15 is expressed in a sub-population of CPCs as Sca1^{pos} cells. For better understanding, we adjusted the text and provide additional RT-PCR and Western blot data on KLF15 expression using the whole heart, total CPCs, and Sca1 cell lysates as suggested by the reviewer. These data replaced the former Fig. 5A and B. The slight lesser KLF15 expression in Sca1^{pos} cells in comparison to CPCs may be due to the presence of different putative populations expressing KLF15 in the CPCs fraction.

5. The expression of vWF and CD105 shown in Figure 5E and 5F seem like the same set of data, please explain.

We apologize that we did not mention in the text. As expected we never observed differences between WT baseline and sham mice in different independent experiments including AngII treatment and TAC and therefore, pooled the animals. We now separate the groups and depicted as such in the new Fig. 5E and F.

6. The Quercetin treatment resulted in a decrease in Ki67 positive cells in both WT and KO CPC culture as shown in Figure 7A. Why did Quercetin treatment not affect WT CPC cell number in 7D?

As we mentioned previously, we performed a semi-quantification analysis in Fig. 7D, which complement the observations made by using a more precise quantification by FACS analysis in Fig. 7A. Indeed WT mice showed a slight decrease in Ki67 cells as demonstrated by FACS quantification although this difference was not significant. Important to note is that the difference between Klf15 KO-DMSO and Klf15 KO-quercetin treated cells with was more accentuated when analyzed by FACS in comparison to the semi-quantitative measurement, which indicate the lack of sensitivity of this complementary technique. We indicate the non-significant difference in figure 7A.

Referee # 3

Dear Referee #3:

We appreciate the time you invested for the evaluation of our revised manuscript. We include additional controls in the manuscripts as well as discussed your questions or concerns.

1. The co-IP experiments to address endogenous interactions are not convincing (Fig. 1). Control antibodies should be used in all cases, not just in 1A, and multiple independent IPs should be shown.

We included the immunoprecipitation IgG controls in all the sub-figures. We also provided 4 independent immunoprecipitation assays (please see below).

We are aware of the sub-optimal quality of the endogenous immunoprecipitation analysis when compared with the immunoprecipitation assay upon overexpression of a tagged-protein. We would like to mention here that while performing this study we checked different antibodies against either N- or C-terminal epitopes of the KLF15 protein and only the KLF15 antibody from Santa Cruz (sc-34826/C-14) was suitable for western blot analysis. We evaluated the suitability of the selected antibody against KLF15. These experiments are added in the new Supporting Information Fig. S1B.

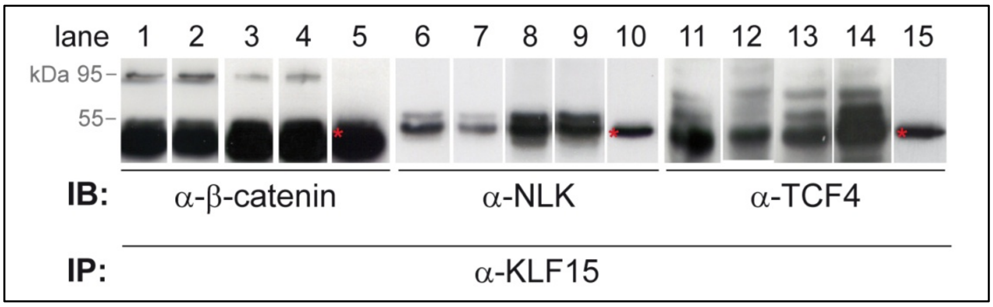

The figure provides quadruplicate IP analysis for the endogenous interactions in adult cardiac tissue shown in Fig. 1 of the manuscript. KLF15 was immunoprecipitated with an anti-KLF15 antibody. Detection of β-catenin (92 kDa, lanes 1-4); NLK (56 kDa, lanes 6-9) as well as TCF4 (approximately 66 and 72 kDa, lanes 11-14) was performed using specific antibodies against the different proteins. Lanes 5, 10 and 15 showed the corresponding controls for IPs. (*) IgG heavy chain 50 kDa.

2. Alternative small molecule inhibitors of Wnt/beta-catenin exist (e.g., ICG-001 and XAV-939 - Selleck Chemicals). I understand the difficulty the authors experienced with RNAi-based approaches for disrupting the pathway. However, it is imperative that they use an agent other than quercitin to confirm the role of beta-catenin in the control of the phenotype in KLF15 KO cells.

We would like to mention that the reason to choose Quercetin as Wnt signaling inhibitor was based on a large literature research to find an appropriate inhibitor to our specific question. As demonstrated very consistently in our manuscript the effect of KLF15 dependent Wnt transcriptional activity depend on the regulation of the TCF4 transcription factor but is independent from b-catenin regulation in vitro and in vivo. Therefore, our search was focused on factors able to block specifically TCF4 activation and Quercetin was the only compound that showed this characteristic [1]. The main source for this search was the official Wnt homepage (http://www.stanford.edu/group/nusselab/cgi-bin/wnt/), which contains all the information about the protein interactions and regulation of Wnt signaling components. Furthermore, we took into account the relevance of this compound for future interventions of this pathway in vivo. In this context, Quercetin demonstrated pharmacological activities specifically in the heart by attenuating hypertrophic changes and showing protection effect in cardiac remodeling with very low toxicity risk [2, 3]. Most importantly, Quercetin was not reported to have an effect "per se" in cardiomyogenesis, which would interfere with the expected rescue effect in our co-culture system.

Beside several limitations (listed below), we believe that repetition of the same technique, namely a compound-mediated inhibition of Wnt signaling on cardiac progenitor cell co-cultures, in which only a different (not adequate) compound is employed will provide further consistency to the core of our study.

Limitations:

- *We question the use of inhibitors unsuitable in our study, specifically XAV939 and ICG-001:*

© EMBO

- *XAV939 is a tankyrase1/2 inhibitor, which stimulates β-catenin degradation by stabilizing Axin, a component of the β-catenin degradation complex in the cytosol [4]. The first and maybe most critical point is that the effect on Wnt inhibition is at the level of the β-catenin destruction complex. Given the fact, that dysregulation of KLF15 does not show any effect on β-catenin level (neither mRNA nor protein), we see this molecule not adequate for our hypothesis. Secondly, this molecule was shown to robustly promote cardiomyogenesis in mouse embryonic stem cells at the expense of other mesodermal derived lineages including endothelial lineage [5]. This effect would interfere with the interpretation of our results, when analyzing endothelial vs. cardiomyogenic cell formation.*

- *The ICG-001 small molecule down-regulates Wnt/β-catenin signaling by specifically binding to cyclic AMP response element-binding protein (CBP) and blocking the CBP/β-catenin interaction in tumor cell lines [6]. However, the authors showed in a second publication the ability of ICG-001 to block both the CBP/γ-catenin interaction and the CBP/β-catenin interactions [7]. This inhibition finally leads to specific regulation of genes involved in apoptosis selectively in cancer cells [6], which made arguable the analysis of such an effect in cardiac progenitor cells. Moreover, as explained above, targeting β-catenin interaction to other transcriptional activator different as TCF4 arises concerns about the relevance of such an inhibitor in Klf15 KO.*

- *Finally, since manipulation of primary cardiac progenitor cells is very difficult, such an assay would require a very fine setup regarding concentration, timing and duration of treatment (as done for Quercetin), a very time consuming task, not doable in less than 3 months.*

In summary, we would like to emphasize that as a part of our study, we provide in vivo data showing that by inverting the cardiac b-catenin/TCF activation in Klf15 KO in vivo, namely a specific b-catenin/TCF signaling reduction we observed opposite phenotype concerning the CPCs endothelial cell fate. b-catenin/TCF transcriptional activation in Klf15 KO mice promotes an endothelial phenotype of CPCs whereas inactivation of this signaling showed less endothelial commitment in the adult heart. Complementing this observation, our in vitro evidence, using Quercetin in a rescue experiment, further supports and confirms the role of KLF15-dependent b-catenin/TCF transcriptional activation in CPC fate decision. We believe that for the scope of the present study these findings provide convincing evidences favoring the role of the b-catenin/TCF-mediated cell fate switch of cardiac progenitor downstream KLF15.

References:

1. *Park, C.H., et al., Quercetin, a potent inhibitor against beta-catenin/Tcf signaling in SW480 colon cancer cells. Biochem Biophys Res Commun, 2005. 328(1): p. 227-34.*
2. *Han, J.J., et al., Quercetin prevents cardiac hypertrophy induced by pressure overload in rats. J Vet Med Sci, 2009. 71(6): p. 737-43.*
3. *Bartekova, M., et al., Acute treatment with polyphenol quercetin improves postischemic recovery of isolated perfused rat hearts after global ischemia. Can J Physiol Pharmacol, 2010. 88(4): p. 465-71.*
4. *Huang, S.M., et al., Tankyrase inhibition stabilizes axin and antagonizes Wnt signalling. Nature, 2009. 461(7264): p. 614-20.*
5. *Wang, H., J. Hao, and C.C. Hong, Cardiac induction of embryonic stem cells by a small molecule inhibitor of Wnt/beta-catenin signaling. ACS Chem Biol, 2011. 6(2): p. 192-7.*
6. *Emami, K.H., et al., A small molecule inhibitor of beta-catenin/CREB-binding protein transcription [corrected]. Proc Natl Acad Sci U S A, 2004. 101(34): p. 12682-7.*
7. *Kim, Y.M., et al., The gamma catenin/CBP complex maintains survivin transcription in beta-catenin deficient/depleted cancer cells. Curr Cancer Drug Targets, 2011. 11(2): p. 213-25.*
