# Supplementary material for: Krueppel-like factor 15 regulates Wnt/β-catenin transcription and controls cardiac progenitor cell fate in the postnatal heart
Source: EMBO Mol Med. 2012 Jul 5;4(9):992–1007. doi: 10.1002/emmm.201101043 (PMC3491830; doi:10.1002/emmm.201101043)
Supplement: Supplementary file 2 [file emmm0004-0992-SD2.pdf]

Supporting Information

***Krueppel*-like factor 15 regulates Wnt/β-catenin transcription and controls cardiac progenitor cell fate in the postnatal heart**

Claudia Noack[1,2], Maria-Patapia Zafiriou[1], Hans-Jörg Schäffer[2], Anke Renger[1], Elena Pavlova[1], Dietz Rainer[2], Wolfram H. Zimmermann[1], Martin W. Bergmann[3] and Laura C. Zelarayán[1]

- 7 Supplemental Figures including Figure legends
- 3 Supplemental Tables

Supplemental Figure S1

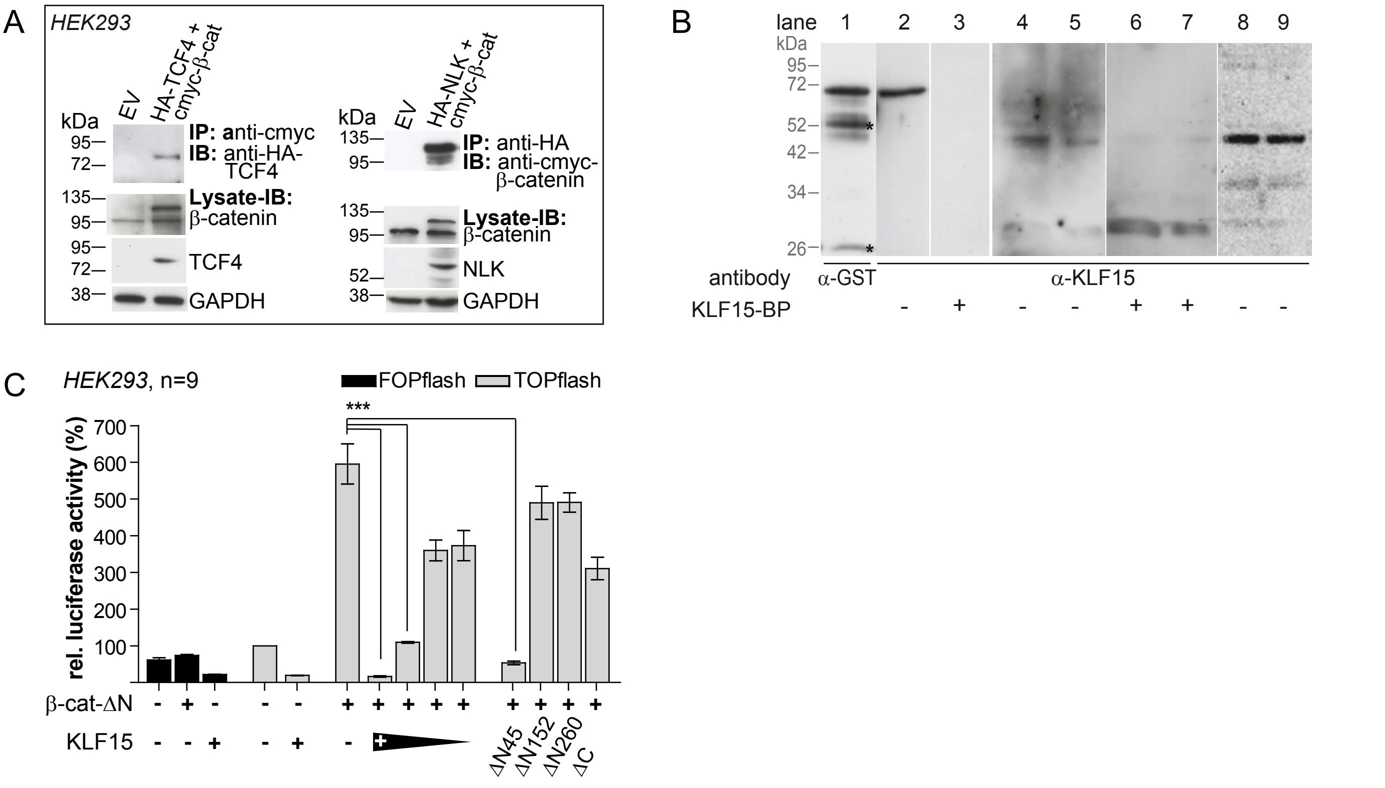

(A) Control co-IP showing known β-catenin/TCF4 and β-catenin/NLK interaction in HEK293 cells. Empty vector (EV) control; GAPDH served as loading control.

(B) Evaluation of the sc-34826 antibody against KLF15 used for endogenous detection and immunoprecipitation. A recombinant GST-KLF15 was used as positive control (lane 1-3). A competition assay employing a blocking peptide (BP) was used to check the specificity of the antibody.

Lane 1: detection of KLF15 in cells expressing GST-KLF15 using an anti-GST antibody showed a 70 kDa band in Western blot (KLF15 44 kDa + GST-tag 26 kDa, a GST dimer of 52 kDa can be detected (*) as well).

Lane 2: detection of KLF15 using the sc-34826 antibody showed the 70 kDa band for GST-KLF15.

Lane 3: competition assay using a specific BP abolished the detection of the 70 kDa band.

Lanes 4-7: endogenous detection of the 44 kDa KLF15 band was observed in whole heart cell lysate in *Klf15* WT (lane 4) and KO (lane 5) mice. This band was not detected after incubation with the BP while unspecific bands where still present in WT (lane 6) and KO (lane 7) mice.

Lanes 8 and 9: endogenous detection of KLF15 in cardiac nuclear cell fraction of WT (lane 8) and KO (lane 9) mice. This antibody recognizes an epitope localized at the C-terminus of KLF15, which is still translated in the non-functional KLF15 KO protein.

(C) HEK293 cells were transfected with the LEF/TCF-dependent firefly luciferase reporter (pTOPflash), non-degradable β-catenin (β-cat-ΔN) and full-length or truncated KLF15 forms. KLF15-full length and -ΔN45 represses β-catenin-LEF/TCF-mediated signaling. KLF15 constructs lacking longer N-terminal regions and KLF15-ΔC did not show repression. pFOPflash served as negative control and *Renilla* luciferase for normalization; data represent mean ± SEM; ANOVA, Bonferroni’s Multiple Comparison Test, *** $P<0.001$.

Supplemental Figure S2

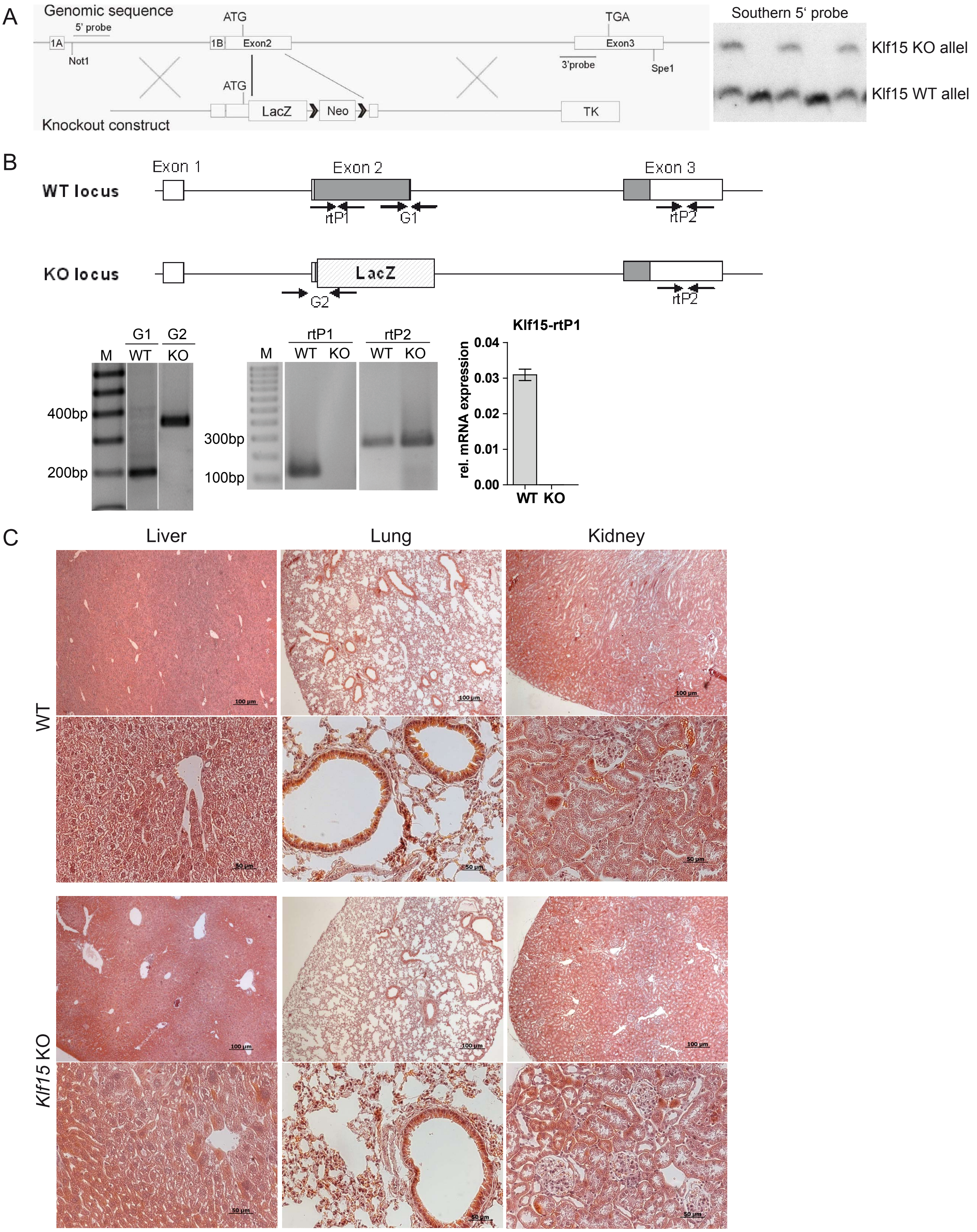

***Klf15* KO mouse model**
(A) KLF15 *loss-of-function* was achieved by replacement of exon 2 of the Klf15 coding region by a partial lacZ-Neo cassette . A representative Southern analysis using the indicated 5' probe to detect *Klf15* KO and WT alleles is shown. **(B)** Genotyping (G1 and G2) and QRT-PCR (rtP1 and rtP2) primers are indicated in the *Klf15* WT and KO gene locus. The rtP1 primers binding in exon 2 allows distinguishing between WT and KO KLF15 mRNA since this region is not present in the Klf15 KO sequence. The use of the rtP2 primers binding in exon 3 of the Klf15 coding region would not show differences between the WT and Klf15 KO transcripts. QRT-PCR analysis of murine cardiac tissue demonstrated no KLF15 RNA expression in *Klf15* KO when the rtP1 primers were employed. **(C)** Hematoxilin/Eosin staining of liver, lung, and kidney tissue of *Klf15* KO mice showed no structural defects in comparison to WT mice. Scale bar 100µm and 50µm.

Supplemental Figure S3

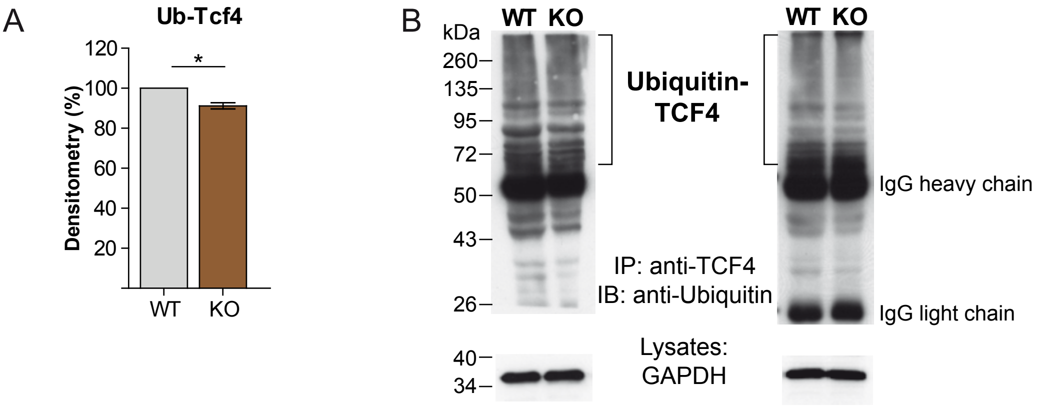

KLF15 influences cardiac TCF4 ubiquitination.
(A) Endogenous ubiquitination of cardiac TCF4 was significantly decreased in *Klf15* KO vs. WT mice. Ub-TCF4 was normalized against the respective IgG heavy chain band. Data represent mean ± SEM; two-tailed Student's *t*-test, * $P<0.05$, n=6. **(B)** Representative immunoblots of cardiac cell lysates from two different experiments showing less ubiquitinated TCF4 in *Klf15* KO hearts. GAPDH shows equal total protein content of heart lysates used for TCF4-IP.

Supplemental Figure S4

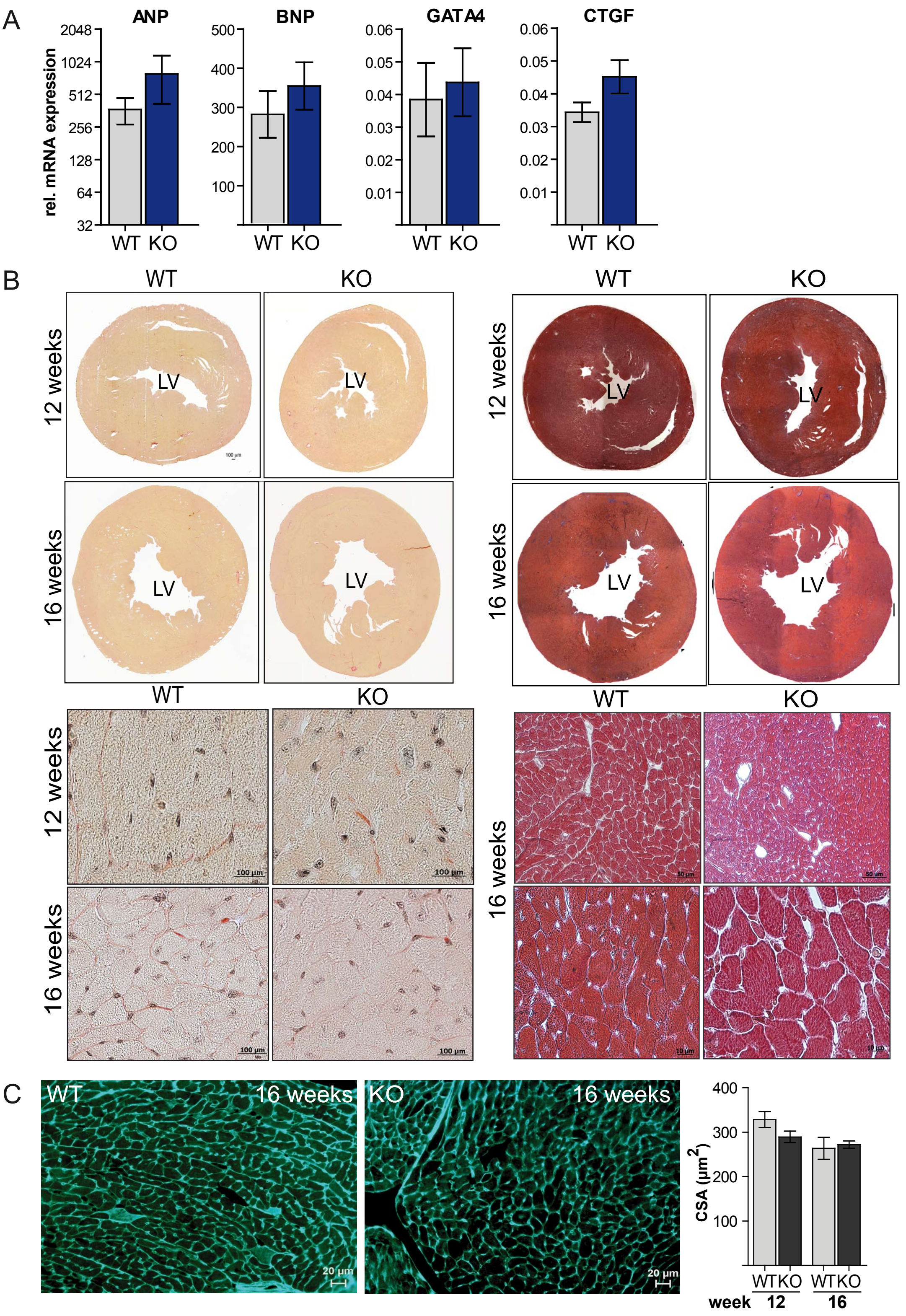

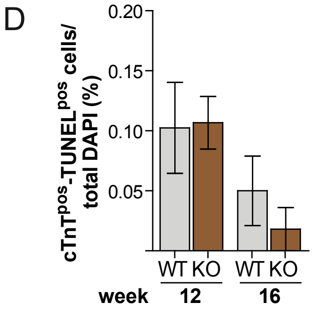

Hypertrophy, fibrosis, and apoptosis analyses of *Klf15* KO and WT mice

(A) QRT-PCR analysis of 16-week-old *Klf15* KO and WT mice (n=10) showed no significant difference of hypertrophic markers ANP and BNP; GATA4; and CTGF expression between KO and WT mice. **(B)** Histological analysis of cardiac fibrosis by Sirius-Red and Masson-Trichrome staining of *Klf15* KO and WT mice. None of the compared groups revealed exaggerated collagen deposition. Scale bar 100µm and 50µm; LV, left ventricle. **(C)** Quantification of cardiomyocyte hypertrophy as measured by cross-sectional area (CSA) revealed no differences between *Klf15* KO vs. WT mice (n=5); representative wheat germ agglutinin (WGA) FITC stainings. **(D)** Apoptosis assessed by TUNEL assay showed no significant differences between *Klf15* KO vs. WT 12- or 16-week-old mice (n=5). Data were analyzed with two-tailed Student's *t*-test.

Supplemental Figure S5

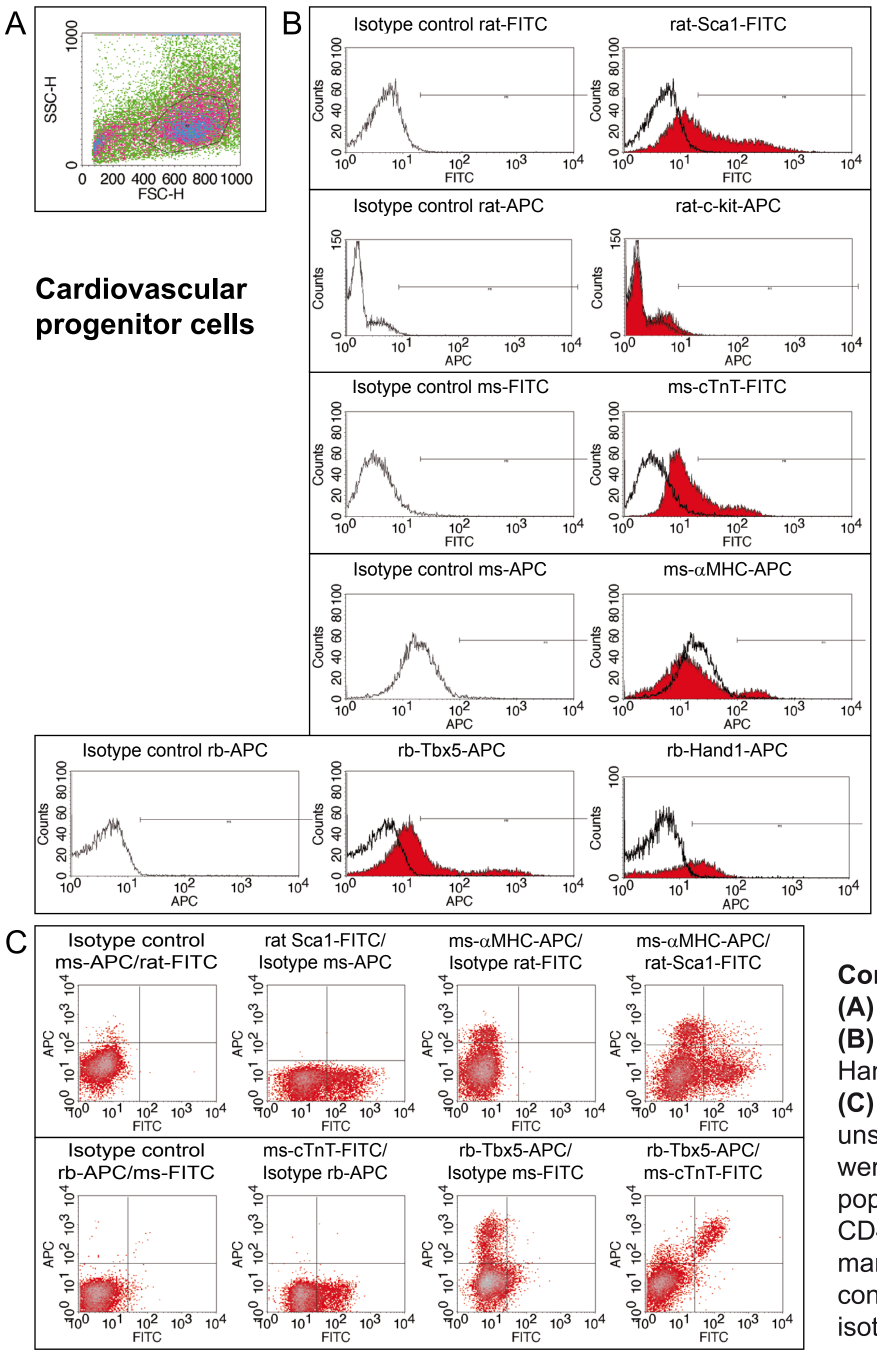

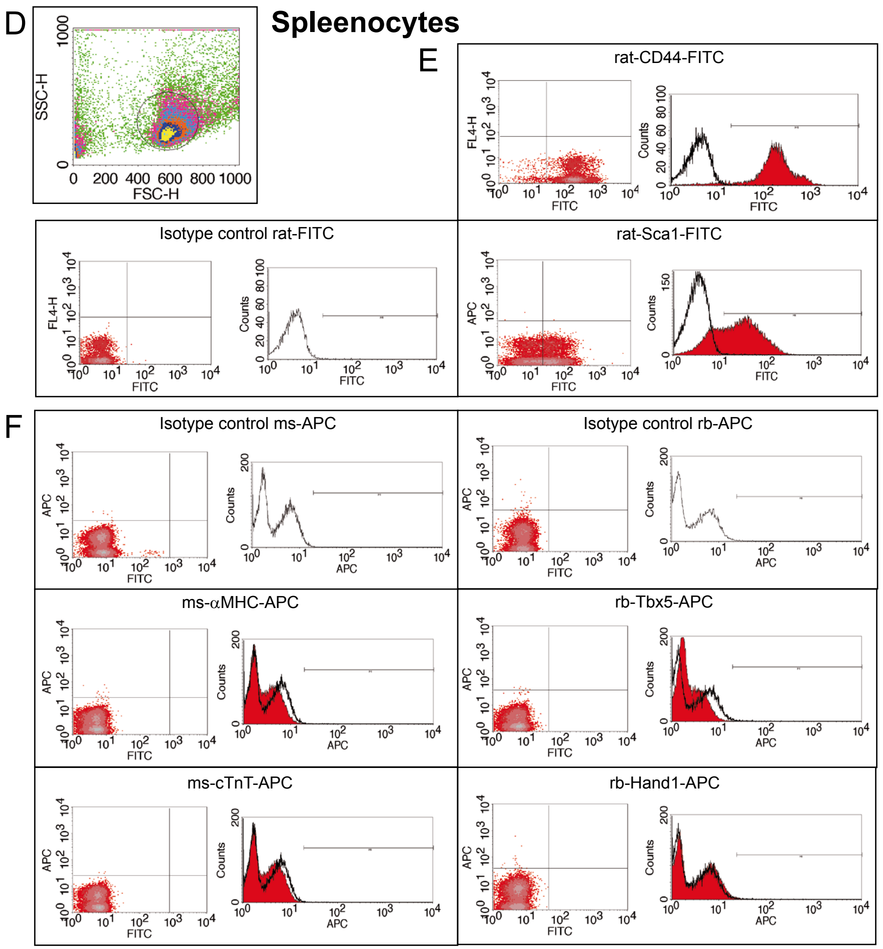

Controls used for staining in FACS analysis of CPCs obtained from adult heart

(A) Forward (FSC) and sideward scatter (SSC) plot showing the CPC population analyzed in our study. **(B)** Histogram plots of gated cells stained with antibodies against Sca1, c-kit, cTnT, αMHC, Tbx5, and Hand1 (red area) and respective isotype controls (white area) are shown as overlays in histogram plots. **(C)** For double staining as Sca1/aMHC and cTnT/Tbx5 dot plots with quadrant marks are depicted. No unspecific cell count was detected with the respective isotype controls for the antibodies. **(D)** Spleen cells were used as positive and negative control for staining in FACS analysis. FSC and SSC plot showing the population of spleen cells analyzed in FACS. **(E)** The spleen cell population was labeled with the marker CD44 and Sca1, typically expressed in those cells. **(F)** The same cell population was stained for cardiac markers cTnT, αMHC, Tbx5, and Hand1. These markers were not detected in the spleen population confirming the specificity of the antibodies used in our study. The histogram plots are showing overlays of isotype controls (white area) and stainings with cardiac antibodies (red area).

Supplemental Figure S6

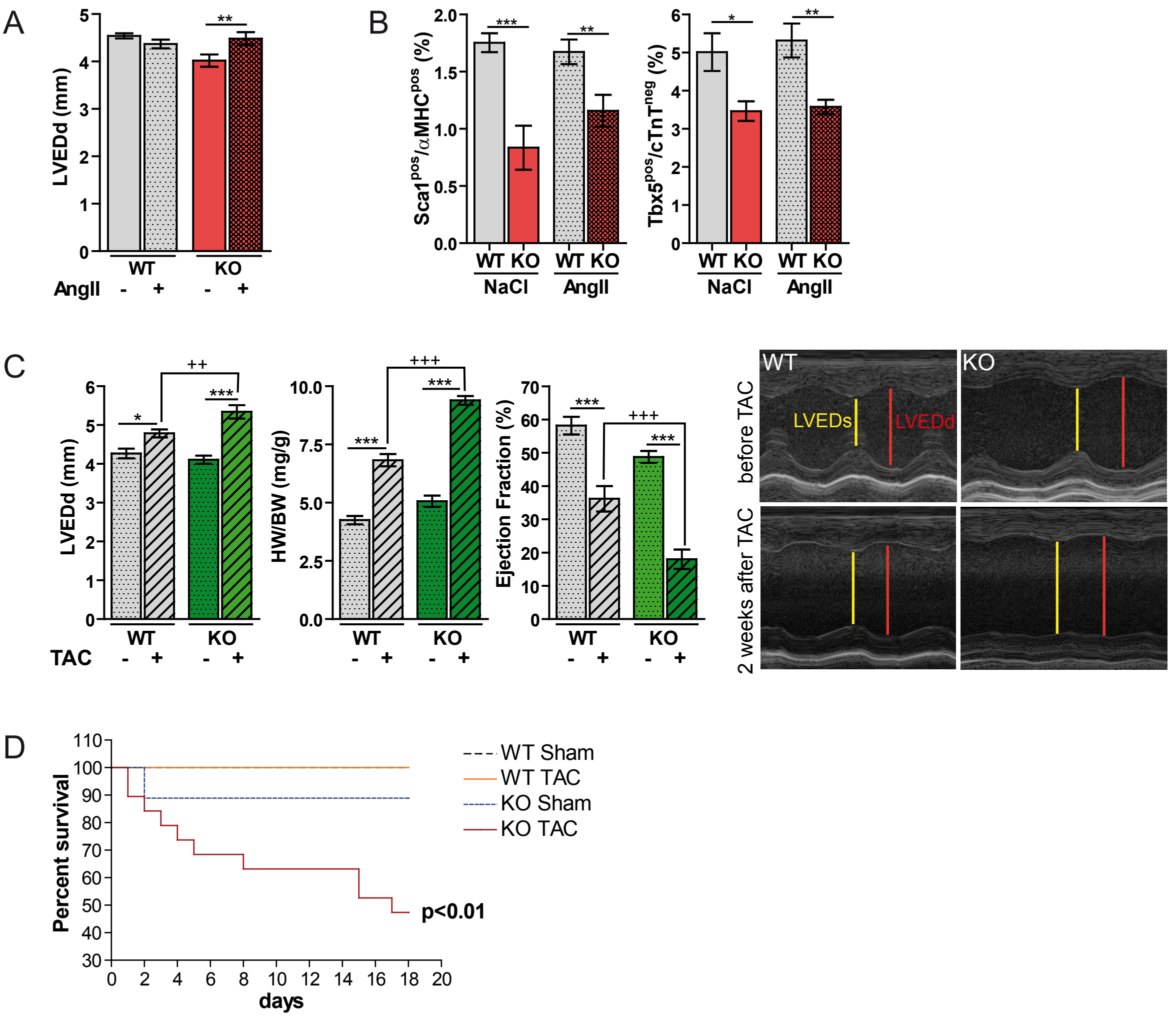

Effect of KLF15 depletion in the adult stressed heart

(A) Cardiac dilation in *Klf15* KO began prior to WT mice 2 weeks following AngII-induced cardiac stress (n≥9) as shown by left ventricular end-diastolic diameter (LVEDd) measurement. **(B)** FACS analysis of isolated CPCs showed a significantly decrease of cardiogenic Sca1^{pos}/αMHC^{pos} and Tbx5^{pos}/cTnT^{neg} populations in *Klf15* KO vs. WT mice after 2-week AngII treatment (n≥9). **(C)** Stronger left ventricular dilation (LVEDd), increased heart-to-body weight ratio (HW/BW) along with more accentuated cardiac deterioration in *Klf15* KO vs. WT mice (n≥8) 2 weeks after TAC as shown by echocardiography analysis. Data represent mean ± SEM; ANOVA, Bonferroni's Multiple Comparison Test, * P<0.05, ** and ++ P<0.01, *** and +++ P<0.001. **(D)** Kaplan-Meier survival curve showed a significantly increased mortality of *Klf15* KO vs. WT mice after TAC (n≥10; Logrank Test, P=0.0034).

Supplemental Figure S7

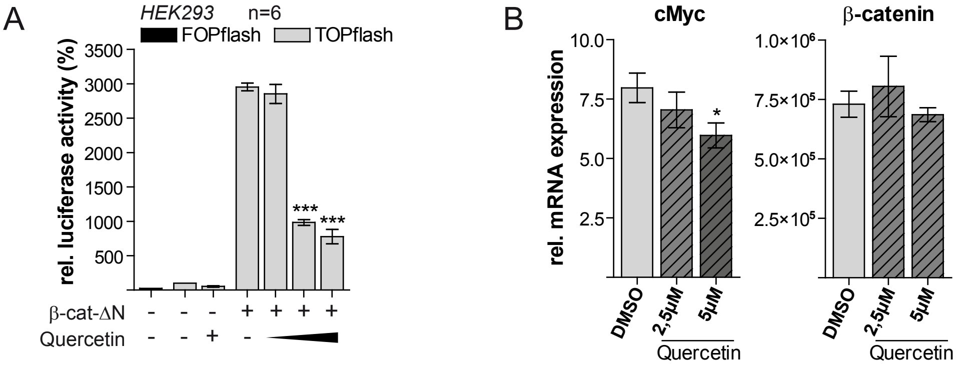

Quercetin inhibits β-catenin dependent transcription

(A) HEK293 cells were transfected with the LEF/TCF-dependent firefly luciferase reporter (pTOPflash) and non-degradable β-catenin (β-cat-ΔN). Quercetin significantly inhibited β-catenin-reporter activation in a concentration-dependent manner (n=6). pFOPflash was used as negative control, *Renilla* luciferase was used for normalization; data represent mean ± SEM; ANOVA, Bonferroni's Multiple Comparison Test, *** P<0.001 vs. TOPflash+β-cat-ΔN. **(B)** QRT-PCR analysis of the β-catenin target gene cMyc revealed a concentration-dependent reduction of its transcriptional activity in co-cultured CPCs treated with Quercetin for 10 days, although β-catenin level was unchanged. Data represent mean ± SEM; two-tailed Student's *t*-test, * P<0.05.

Table S1

A) Echocardiography analysis of *Klf15* WT and KO mice at different age

age group (weeks)	Klf15 genotype	IVSd [mm]	LVPWd [mm]	LVEDd [mm]	FS [%]	EF [%]	HW/BW [mg/g]	HW/TL [mg/mm]
8	WT	0.60 ± 0.02	0.60 ± 0.02	4.07 ± 0.09	28.1 ± 2.19	51.2 ± 3.13	n.d.	n.d.
	KO	0.58 ± 0.02	0.60 ± 0.03	3.88 ± 0.11	24.6 ± 1.88	45.8 ± 2.14	n.d.	n.d.
12	WT	0.75 ± 0.04	0.70 ± 0.03	4.21 ± 0.09	29.8 ± 1.61	54.8 ± 2.84	4.54 ± 0.16	7.71 ± 0.34
	KO	0.73 ± 0.03	0.76 ± 0.03	4.14 ± 0.14	22.2 ± 2.66	42.8 ± 4.24	5.01 ± 0.18	7.03 ± 0.94
16	WT	0.72 ± 0.02	0.72 ± 0.03	4.27 ± 0.19	30.2 ± 3.18	57.3 ± 4.64	5.11 ± 0.12	7.94 ± 0.35
	KO	0.76 ± 0.02	0.77 ± 0.03	4.52 ± 0.12	23.3 ± 3.76 *	46.0 ± 6.50 *	5.44 ± 0.18	8.60 ± 0.38

n.d.=not determined; n=10; * P<0.05 Student's *t*-test, compared to WT group of same age

B) Echocardiography analysis of βCat$^{\Delta ex3}$ CT and TG mice 16 weeks after Cre-induction

Cre induction	genotype	IVSd [mm]	LVPWd [mm]	LVEDd [mm]	FS [%]	EF [%]	HW/BW [mg/g]	HW/TL [mg/mm]
before	CT	0.72 ± 0.02	0.69 ± 0.03	4.06 ± 0.08	33.61 ± 1.82	62.63 ± 2.50	n.d.	n.d.
	TG	0.76 ± 0.01	0.75 ± 0.02	4.25 ± 0.04	30.63 ± 1.79	58.27 ± 2.66	n.d.	n.d.
16 weeks after	CT	0.68 ± 0.04	0.68 ± 0.04	4.25 ± 0.13	32.76 ± 2.23	61.31 ± 3.28	4.19 ± 0.12	7.01 ± 0.14
	TG	0.77 ± 0.03	0.71± 0.00	4.61 ± 0.29	22.41 ± 2.37 *	45.15 ± 4.21 *	4.74 ± 0.18 *	7.46 ± 0.13

n=7; * P<0.05 Student's *t*-test, compared to CT group of the same time point

Table S2

A) Echocardiography analysis of *Klf15* WT and KO mice after 2 weeks AngII treatment

Klf15 genotype	treatment	n	IVSd [mm]	LVPWd [mm]	LVEDd [mm]	FS [%]	EF [%]	HW/BW [mg/g]
WT	NaCl	15	0.69 ± 0.02	0.67 ± 0.02	4.54 ± 0.05	25.4 ± 1.51	49.5 ± 2.40	4.17 ± 0.16
WT	AngII	13	0.83 ± 0.03 ***	0.83 ± 0.03 ***	4.37 ± 0.09	22.5 ± 1.17	45.3 ± 2.03	4.94 ± 0.16 *
KO	NaCl	10	0.87 ± 0.05	0.84 ± 0.04	4.02 ± 0.13	28.0 ± 1.43	54.0 ± 4.78	4.85 ± 0.33
KO	AngII	9	0.87 ± 0.03	0.85 ± 0.02	4.48 ± 0.14 ††	23.6 ± 2.89	46.4 ± 5.00	5.37 ± 0.38

ANOVA, post test: Bonferroni's Multiple Comparison Test
* P<0.05 and *** P<0.001 compared to WT+NaCl; †† P<0.01 compared to KO+NaCl

B) Echocardiography analysis of *Klf15* WT and KO mice 2 weeks after TAC

Klf15 genotype & OP	n	desc. Gradient [mm/Hg]	IVSd [mm]	LVPWd [mm]	LVEDd [mm]	FS [%]	EF [%]	HW/BW [mg/g]	HW/TL [mg/mm]
WT-Sham	8	4.25 ± 0.43	0.74 ± 0.02	0.75 ± 0.03	4.27 ± 0.12	30.8 ± 1.80	58.2 ± 2.66	4.25 ± 0.18	6.22 ± 0.26
WT-TAC	12	42.4 ± 5.89 ***	0.93 ± 0.02 ***	0.93 ± 0.02 ***	4.79 ± 0.10 *	17.3 ± 2.04 ***	36.2 ± 3.82 ***	6.63 ± 0.27 ***	10.2 ± 0.43 ***
KO-Sham	8	2.63 ± 0.41	0.76 ± 0.03	0.73 ± 0.03	4.11 ± 0.10	24.5 ± 1.22	48.8 ± 1.76	5.06 ± 0.24	6.03 ± 0.35
KO-TAC	10	35.7 ± 3.62 ***	0.92 ± 0.04 **	0.90 ± 0.04 **	5.34 ± 0.17 ***/††	8.45 ± 1.33 ***/††	18.0 ± 2.92 ***/†††	9.39 ± 0.19 ***/†††	11.8 ± 0.23 ***/††

ANOVA, post test: Bonferroni's Multiple Comparison Test
* P<0.05, ** P<0.01, and *** P<0.001 compared to respective Sham group
†† P<0.01 and ††† P<0.001 KO-TAC vs. WT-TAC

Table S3

Primers used for mouse genotyping

Locus	Primer	Sequence (5' → 3')
Klf15 WT	s	CTC AAA ATG CAC AAA TGC AC
	as	ATA CAC TCG GGG GAG CAG
Klf15 KO	s	ATC TGG ACA TTT GGC CAC AG
	as	CTC TTC GCT ATT ACG CCA GC
αMHC-MerCreMer	s	GTC TGA CTA GGT GTC CTT CT
	as	CGT CCT CCT GCT GGT ATA G
β-Cat$^{ex3,flox/flox}$	s	AGA ATC ACG GTG ACC TGG GTT AAA
	as	CAT TCA TAA AGG ACT TGG GAG GTG T
β-cat$^{\Delta ex3}$	s	GCT GCT GTG ACA CCG CTG CGT GGA C
	as	CAC GTG TGG CAA GTT CCG CGT CAT CC
β-Cat$^{ex2-6,flox/flox}$	s	AAG GTA GAG TGA TGA AAG TTG TT
	as	CAC CAT GTC CTC TGT CTA TTC
β-cat$^{\Delta ex2-6}$	s	AAT CAC AGG GAC TTC CAT ACC AG
	as	GCC CAG CCT TAG CCC AAC T

Primers used in QRT-PCR analysis for mRNA expression

Gene	Primer	Sequence (5' → 3')
ANP	s	CAT CAC CCT GGG CTT CTT CCT
	as	TGG GCT CCA ATC CTG TCA ATC
β-Actin	s	CAC ACC CGC CAC CAG TTC
	as	CCC ATT CCC ACC ATC ACA CC
β-catenin	s	ACT GCT GGG ACT CTG
	as	TGA TGG CGT AGA ACA G
BNP	s	AAG TCC TAG CCA GTC TCC AGA GC
	as	CTT CAG TGC GTT ACA GCC CAA AC
CD105	s	ACC CAC AAC AGG TCT CGC AGA AAG
	as	TCC GGG GCC TGG AAC TTC ACG A
CD133	s	GTG GGC TGC TTC TTT TGT ATG TGC
	as	CCG AGT CCT GGT CTG CTG GTT AG
CD31	s	AAC CCG TGG AGA TGT CCA GGC CAG C
	as	ACA CCG TCT CTG TGG CTC TCG TTC CC
cMyc	s	TTC TAT CAC CAG CAA CAG
	as	ATA GGA TGG AGA GCA GAG
Ctgf	s	CCG CCA ACC GCA AGA TCG GA
	as	CGC AGC ATT TCC CAG GCA GC
Flk1	s	GGA TGT GGA CTG GGA GGA AG
	as	AGA GAT GAG GAA GGA GCA AGC
Gapdh	s	ATG TTC CAG TAT GAC TCC ACT CAC G
	as	GAA GAC ACC AGT AGA CTC CAC GAC A
GATA4	s	CTG GAG GCG AGA TGG
	as	GGT GGT GGT AGT CTG G
Klf15	s	GCC TTC TGT TCC TGC TAC
	as	GCT ATC TCC AAT ACC TCC AC
Sca1	s	ACT GTG CCT GCA ACC TTG TCT GAG A
	as	GTC CAG GTG CTG CCT CCA TT
Tcf4	s	AAC GGA ACA GAC AGT ATA ATG G
	as	CAC AGG AGT TGA AGG ATT GG
vWF	s	AGG TGC CTG CCC TCT GCT TGC A
	as	TCC TCC ACG CGG ACA CAC TCG T
